# Global diversity of policy, coverage, and demand of COVID-19 vaccines: a descriptive study

**DOI:** 10.1186/s12916-022-02333-0

**Published:** 2022-04-04

**Authors:** Zhiyuan Chen, Wen Zheng, Qianhui Wu, Xinghui Chen, Cheng Peng, Yuyang Tian, Ruijia Sun, Jiayi Dong, Minghan Wang, Xiaoyu Zhou, Zeyao Zhao, Guangjie Zhong, Xuemei Yan, Nuolan Liu, Feiran Hao, Sihong Zhao, Tingyu Zhuang, Juan Yang, Andrew S. Azman, Hongjie Yu

**Affiliations:** 1 School of Public Health, Fudan University, Key Laboratory of Public Health Safety, Ministry of Education, Shanghai, 200032 China; 2 Shanghai Institute of Infectious Disease and Biosecurity, Fudan University, Shanghai, China; 3 Department of Epidemiology, Johns Hopkins Bloomberg School of Public Health, Baltimore, MD USA; 4 Institute of Global Health, Faculty of Medicine, University of Geneva, Geneva, Switzerland; 5 Department of Infectious Diseases, Huashan Hospital, Fudan University, Shanghai, China

**Keywords:** COVID-19 vaccines, Vaccination policy, Vaccine coverage, Vaccine demand, Global diversity

## Abstract

**Background:**

Hundreds of millions of doses of coronavirus disease 2019 (COVID-19) vaccines have been administered globally, but progress on vaccination varies considerably between countries. We aimed to provide an overall picture of COVID-19 vaccination campaigns, including policy, coverage, and demand of COVID-19 vaccines.

**Methods:**

We conducted a descriptive study of vaccination policy and doses administered data obtained from multiple public sources as of 8 February 2022. We used these data to develop coverage indicators and explore associations of vaccine coverage with socioeconomic and healthcare-related factors. We estimated vaccine demand as numbers of doses required to complete vaccination of countries’ target populations according to their national immunization program policies.

**Results:**

Messenger RNA and adenovirus vectored vaccines were the most commonly used COVID-19 vaccines in high-income countries, while adenovirus vectored vaccines were the most widely used vaccines worldwide (180 countries). One hundred ninety-two countries have authorized vaccines for the general public, with 40.1% (77/192) targeting individuals over 12 years and 32.3% (62/192) targeting those ≥ 5 years. Forty-eight and 151 countries have started additional-dose and booster-dose vaccination programs, respectively. Globally, there have been 162.1 doses administered per 100 individuals in target populations, with marked inter-region and inter-country heterogeneity. Completed vaccination series coverage ranged from 0.1% to more than 95.0% of country target populations, and numbers of doses administered per 100 individuals in target populations ranged from 0.2 to 308.6. Doses administered per 100 individuals in whole populations correlated with healthcare access and quality index (*R*^2^ = 0.59), socio-demographic index (*R*^2^ = 0.52), and gross domestic product per capita (*R*^2^ = 0.61). At least 6.4 billion doses will be required to complete interim vaccination programs—3.3 billion for primary immunization and 3.1 billion for additional/booster programs. Globally, 0.53 and 0.74 doses per individual in target populations are needed for primary immunization and additional/booster dose programs, respectively.

**Conclusions:**

There is wide country-level disparity and inequity in COVID-19 vaccines rollout, suggesting large gaps in immunity, especially in low-income countries.

**Supplementary Information:**

The online version contains supplementary material available at 10.1186/s12916-022-02333-0.

## Background

The coronavirus disease 2019 (COVID-19) pandemic is still raging globally, with variants such as Omicron emerging and spreading widely, and with unprecedented impact on societies and economies [1]. Several non-pharmaceutical interventions (NPIs) have been effective to reduce virus transmission [2, 3], but it is unrealistic to continue many of these NPIs for long-term maintenance. Rapid and successful development of efficacious and effective vaccines against severe acute respiratory syndrome coronavirus-2 (SARS-CoV-2) and its variants is making it possible to manage the pandemic [4].

General public health use of COVID-19 vaccines started in December 2020 and has accelerated globally at an unprecedented rate. However, inequitable country-level access to vaccines may unbalance global immunity and will likely impede global reopening. People may not get vaccinated for a variety of reasons, including supply-and-demand challenges, country purchase capacity, not being a member of a country-specific vaccine target population, lack of perceived risk, inconvenience, and personal hesitancy to receive vaccines [5–10]. Most high-income countries have purchased vaccine directly using advanced purchase agreements with vaccine developers and manufacturers [5]. Low-income countries were less able to purchase/receive vaccines, although global efforts like COVID-19 Vaccine Global Access (COVAX) increase access [11].

Disparities in COVID-19 vaccination progress have been observed between and within countries. Several dashboards use official data sources to track vaccination progress, but there has been insufficient focus on variation in countries’ vaccination policies [1, 12]. One study explored the correlation between gross domestic product (GDP) per capita and vaccine coverage in 138 countries and showed disparities in vaccine rollout among countries with different income levels [13]. Country-level spatiotemporal disparities in vaccination were also seen [14, 15]. Descriptive analyses of vaccination campaigns across countries, especially of vaccination policy and policy-specific demand, have not been realized to the extent necessary for upcoming policy formulation.

In this study, we aimed to describe the global landscape of COVID-19 vaccination policy by authorized vaccines, primary/booster vaccination, and target population. We constructed metrics for vaccine coverage to explore the extent and variation in global and regional vaccination progress, and we estimated specific demand for vaccine.

## Methods

We constructed datasets for vaccination policy and doses administered as of 8 February 2022 that contain country-specific metrics. We used these metrics to develop three coverage indicators and estimated demand for vaccine doses. Detailed descriptions of data collection methods and data sources are provided in Additional file 1.

### Metrics

Policy metrics include authorization status of COVID-19 vaccines (approved, conditionally approved, or authorized for emergency use), vaccination schedules, indicated age groups, contraindications, whether vaccines are sold/donated by or received by the country, and whether local residents need to pay for vaccine (Additional file 1: Table S1-S4, Fig. S1). Vaccination schedules mainly include dose intervals according to regulatory authorities, and special doses, such as additional doses for immunocompromised individuals and booster doses. Contraindications are medical conditions for which individuals should not be vaccinated, either temporarily for conditions that resolve or permanently.

The dose-administered dataset included four time-varying, age-specific, platform-specific metrics: number of vaccine doses administered, number of people receiving at least one dose, number of people fully vaccinated according to country schedule, and number of people receiving additional/booster doses. People receiving at least one dose are those who have received the first dose of a multiple-dose vaccine or the dose of a one-dose vaccine. The number of people fully vaccinated indicates those who have completed their primary immunization series according to their country’s vaccination schedule. Additional doses are doses for people with medical conditions requiring doses beyond the primary series to achieve immunity (e.g., moderately to severely immunocompromised individuals); booster doses are doses given after the primary series to counter waning immunity or decreasing protection [16].

### Target populations

Target populations are eligible individuals who are within approved age groups and without contraindications according to the country’s immunization policy. When country-specific information was unavailable, we used the World Health Organization (WHO)-recommended ages (as of 8 February 2022), in which people 5 years and older are recommended to receive BNT162b2 vaccine and people 18 years and above are recommended to receive any COVID-19 vaccine [17]. Contraindications vary by country and may include pregnant women, people with certain underlying conditions (i.e., bleeding disorders and immune suppression), and/or those previously infected with SARS-CoV-2 (Additional file 1: Table S2, Table S5) [1, 18–21]. We estimated sizes of target populations for primary, additional, and booster doses according to country-specific policy (Additional file 1: Table S6). To estimate the number of individuals with bleeding disorders or immune suppression, we adapted Clark’s method to adjust for effects of clustering and multimorbidity using data from the Global Burden of Diseases [21] and two multi-morbidity studies [22, 23], considering that one person can simultaneously suffer more than one sub-category of such a disease [20]. Methods for estimating target population sizes are detailed in Additional file 1.

### Vaccine coverage

We constructed vaccine coverage indicators for both total populations and target populations. The indicators are full vaccination coverage, proportion receiving at least one dose, and doses administered per 100 people. Because not all countries report vaccine doses administered on a daily basis, we used linear extrapolation between reported data points for missing internal data points. For example, if vaccination volumes on March 1 and March 3 were reported but no volume was reported for March 2, we assumed that the vaccination volume on March 2 was midway between the March 1 and March 3 values. We estimated vaccine coverage through 31 January 2022. For countries that did not report data to 31 January 2022, we used latest data reported on or after 17 January 2022 as the 31 January value, but if no data were reported on/after 17 January, we indicate missing values for 31 January.

### Exploring factors associated with coverage

We identified a list of economic, social, health spending, health resources-related factors that are known or believed to be associated with vaccine coverage (Additional file 1: Table S7) [19, 24–28], including socio-demographic index (SDI), healthcare access and quality (HAQ) Index, GDP per capita adjusted for purchasing power parity (PPP), physician density, as well as PPP-adjusted government health spending per capita. SDI is a composite indicator developed by the Institute for Health Metrics and Evaluation, which reflects a country’s socio-demographic level and was proven to correlate highly with health outcome variables [29]. Through calculating geometric means of lag-distributed income per capita, average education level, and fertility rate under 25 years, values range from 0 to 1 and can be divided into five categories: high, high-middle, middle, low-middle, and low SDI [30]. HAQ is a country-specific index that quantifies the accessibility and quality of personal healthcare, ranging from 0 to 100 [25]; physician density mirrors the capacity of healthcare services [19, 27]. PPP-adjusted GDP per capita [26] and government health spending per capita [28] reflect a country’s overall economic and health-related economic level, respectively.

### Demand

We determined demand for vaccine as the number of doses needed to complete vaccination of countries’ target populations according to national immunization program policy. We estimated demand for primary immunization and additional/booster doses separately. We used a simplifying assumption that all COVID-19 vaccines primary series require two doses, as one-dose (Ad26.COV2.S, Ad5-nCoV) and three-dose (ZF2001, CIGB-66) vaccines are thus far a small (though unknown) proportion of total doses administered. We estimated additional and booster doses needed based on sizes of certain populations allowed by regulatory agencies; we assumed one additional/booster dose per person recommended or indicated. We calculated total current demand as the sum of doses required minus doses administered by 31 January 2022, stratified by primary and additional/booster dose demand.

### Data sources

In priority order, we obtained data from government websites, health department websites, official media, vaccine manufacturers’ websites, authoritative media, and local media. We also used data from public databases that systematically collect and cross-check such information, such as Gavi-COVAX [31], COVID-19 Vaccine Market Dashboard [32], and Our World in Data [33]. Whether countries sell/donate or receive vaccine was obtained from COVAX data [31].

We collected raw data through a combination of manual and automated means. For countries that released data on a regular basis through official sources [34, 35], we collected information manually on a weekly basis; for countries that released official data through a public online dataset (e.g., GitHub repository) [36], we accessed data with a compiled R Script. Countries were included in our doses administered datasets if they reported at least two data points. We verified data transcription accuracy with double-entry.

We derived 2020 population estimates from United Nations World Population Prospects [37]. Among 194 WHO Member States, age-specific United Nations population proportions were available for 183 countries. Age-specific population estimates for the 11 remaining countries (Andorra, Cook Islands, Dominica, Saint Kitts and Nevis, Monaco, Marshall Islands, Niue, Nauru, Palau, San Marino, and Tuvalu) were compiled from WorldPop datasets [38].

### Statistical analysis

We explored univariate associations of coverage with SDI, HAQ Index, PPP-adjusted GDP per capita, physician density, and PPP-adjusted government health spending per capita. Multicollinearity of those covariates was accessed by using the variance inflation factor (VIF) in linear model; we did not perform multivariable modeling considering that most variables were collinear (VIF > 5) (Additional file 1: Table S8). Instead, we individually investigated univariate associations of each selected variable with vaccine coverage. We used a linear or non-linear regression model to explore the relationship between vaccine coverage and a series of variables. We chose a linear regression model if a clear linear relationship was observed in scatter plots and the standard residuals were uniformly distributed around zero. Otherwise, we used non-linear regression to fit. For regressing vaccine coverage with PPP-adjusted GDP per capita and government health spending per capita, since there were clear non-linear, logarithmic relationships observed through scatter plots, we used a nonlinear self-starting regression model (logistic growth model) to determine adjusted relations. Model selection was based on scatter plots, regression coefficients, and residual plots. Statistical analyses and visualizations were done using R (version 4.0.2).

## Results

### COVID-19 vaccination policies

As of 8 February 2022, 192 countries reported the COVID-19 vaccines they used and its target population. All countries have extended vaccination to the general public (Additional file 1: Table S2).

**Fig. 1:**
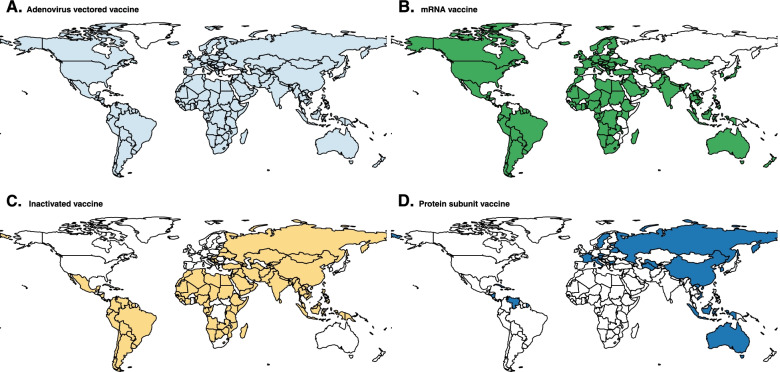
Technical platforms for vaccines being administered across countries. Geographic distribution of adenovirus vectored vaccine (**A**), mRNA vaccine (**B**), inactivated vaccine (**C**), and protein subunit vaccine (**D**) administered across the globe. Since the conjugate vaccine was only used in three countries (Cuba, Iran and Nicaragua), it is not shown in the map

Among the five technological vaccine platforms with vaccines approved in one or more countries, adenovirus vectored vaccines were the most widely used (180 countries), followed by mRNA vaccines (159 countries), inactivated vaccines (116 countries), protein subunit vaccines (10 countries), and conjugate vaccines (3 countries) (Fig. 1). From a regional perspective, high-income countries in Europe and the Americas most often used both mRNA and adenovirus vectored vaccines; other countries in the Americas also used inactivated vaccines (Additional file 1: Fig. S2). In 25 countries in Africa, vaccination was primarily with a combination of inactivated, adenovirus vectored, and mRNA vaccines (Fig. 1). Among 30 countries (18 high-income) that use vaccines made by two or more different platforms, more than half of the vaccines administered in 76.7% (23) of these countries were mRNA vaccines, mainly BNT162b2 vaccine (Additional file 1: Fig. S3-4).

**Fig. 2:**
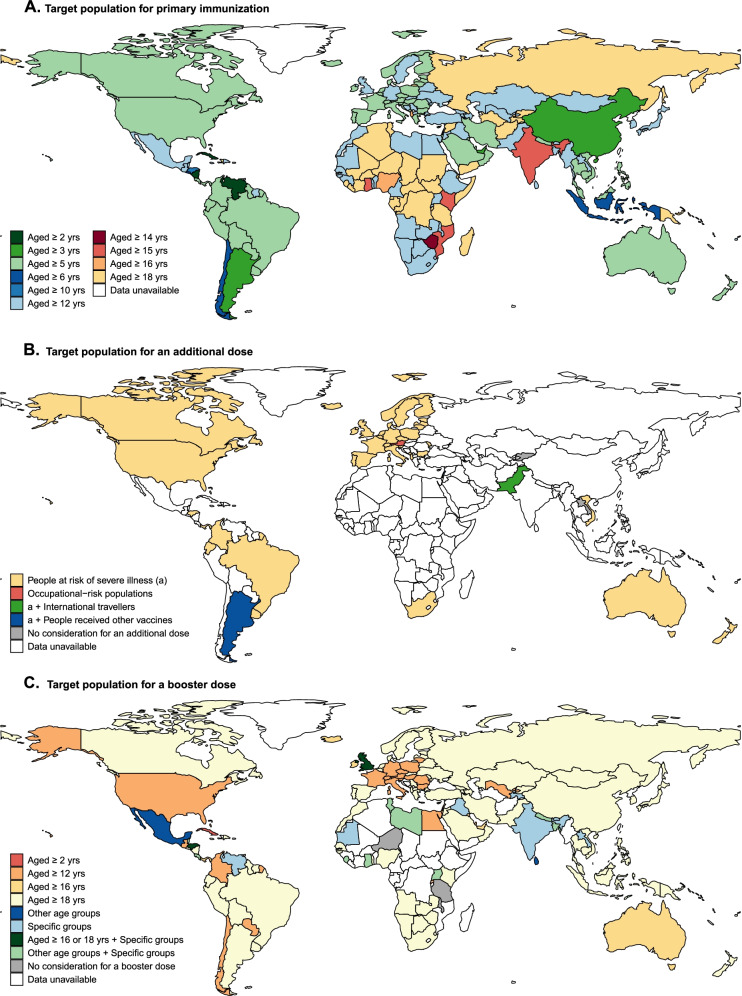
Target populations for primary immunization, additional, and booster doses. Global distribution of target populations (regulator-approved age groups) for primary vaccination (**A**), an additional dose (**B**), and for a booster dose (**C**). Here, specific groups included people at risk of severe illness, occupationally at-risk populations, and international travelers. People at risk of severe illness included the elderly, residents in health centers, people with immunodeficiency disorders, people with autoimmune diseases receiving immunosuppressive therapy, people on dialysis or after organ transplantation, patients with onco-hematological diseases receiving treatment, and people with other comorbidities. Occupational-risk populations included workers at long-term care facilities, front-line health workers, and other front-line workers. People receiving other vaccines included people who receiving the one of the following vaccines: BBIBP-CorV and CoronaVac

Ten different age groups were authorized worldwide for primary immunization. Among 192 countries, vaccines were authorized for those 12 years and over in 77 (40.1%) countries and 5 years and over in 62 (32.3%) countries; seven countries have approved vaccination for 2- or 3-year-olds and above (Fig. 2A). Additional doses were recommended in 48 countries, mainly targeting people at risk of serious illness (47 of 48 countries) (Fig. 2B). Booster doses have been approved for use in 151 countries, but with wide variation in target populations; 140 countries recommend booster doses for the general public, and previously infected individuals can receive booster doses in 18 countries (Fig. 2C). The timing of booster doses varies by country, with most (48 countries) being 6 months after primary immunization (Additional file 1: Table S3). Among 175 countries for which we could determine whether there was a charge for vaccination, all provide COVID-19 vaccines free of charge to residents, with the lone exception of Singapore (not free for BBIBP-CorV vaccine) (Additional file 1: Fig. S1).

### Vaccine coverage

**Fig. 3:**
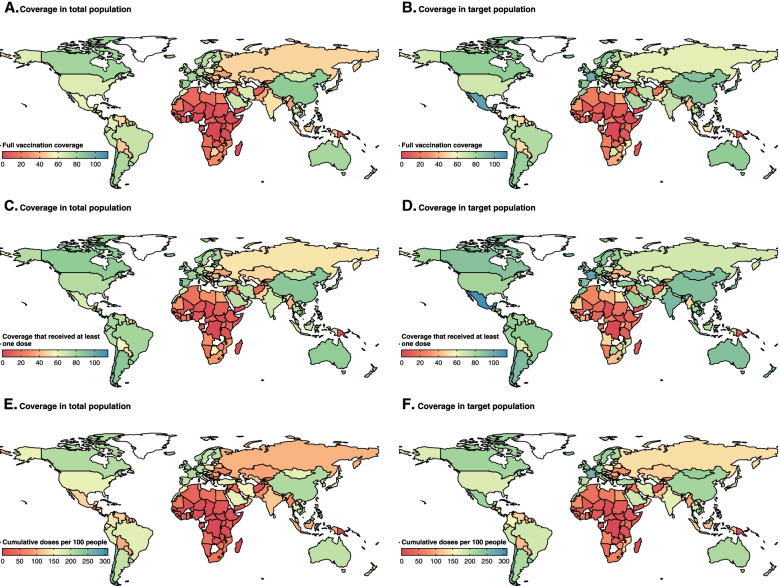
Vaccine coverage among total or target populations. Country-level full vaccine coverage among total populations (**A)** or target populations (**B)**. Country-level proportion of people that received at least one dose among total populations (**C**) or target populations (**D**). Country-level cumulative doses per 100 people among total populations (**E**) or target populations (**F**). The white areas represent countries with no vaccination rollout or for which data are unavailable. The data shown here are as of 31 January 2022. Percentages of target populations that received full or at least one dose that exceed 100% may represent off-label use

Vaccination data for 181 countries were available as of 31 January 2022; 162.1 doses have been administered per 100 individuals in target populations; 77.9% and 66.8% of target populations received at least one dose and full-schedule doses, respectively; 39.8% (70/176) and 29.4% (52/177) of countries have vaccinated more than two thirds of their population with at least one dose and full-schedule doses, respectively (Fig. 3). The amount of time needed to achieve one dose per 100 individuals in their whole population varied by country (Additional file 1: Fig S5).

Highest full-dose target population coverage (86.5%) and doses administered per 100 individuals in target populations (208.3 doses per 100 individuals in target populations) were reported in the WHO Western Pacific Region, followed by the Americas (73.2%, 180.5 doses per 100 target populations), Europe (71.9%, 182.5 doses per 100 target populations), South-East Asia (65.4%, 153.5 doses per 100 target populations), Eastern Mediterranean (49.1%, 113.1 doses per 100 target populations), and Africa (14.1%, 34.5 doses per 100 target populations) (Fig. 3B, F). There was significant inter-country heterogeneity in full-dose coverage among target populations, ranging from 0.1% (Burundi) to more than 95.0% (Fig. 3B), and cumulative doses per 100 individuals in target populations, ranging from 0.2 (Burundi) to 308.6 (Cuba) (Fig. 3F). In high-income countries, 85.5% of target populations have received at least one dose; in low-income countries, 19.4% of target populations have received at least one dose (Additional file 1: Fig. S6). Countries selling or donating vaccine had higher coverage than those receiving vaccine (Additional file 1: Fig. S6-S7).

**Fig. 4:**
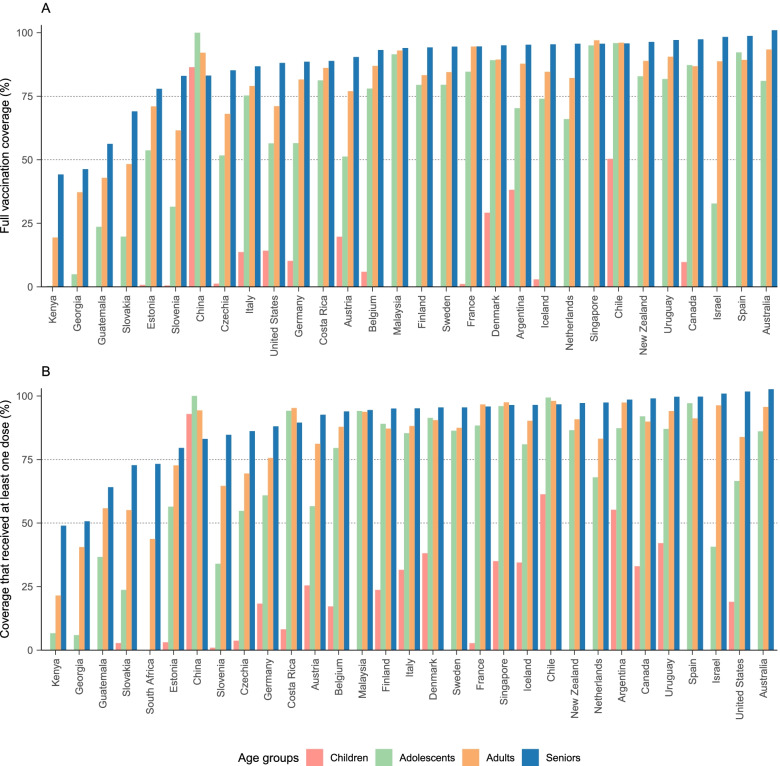
Vaccine coverage by age group. Proportion of age-specific populations that received full doses (**A**) and at least one dose (**B**) among total populations. The dividing line between age groups was not consistent across countries. Children refers to 0–11-year-olds; adolescent refers to 12–17-year-olds; adult refers to people aged 18–64 (Austria, Belgium, Chile, United States), 18–59 years old (Argentina, Canada, China, Czechia, Estonia, Finland, France, Georgia, Germany, Guatemala, Kenya, Malaysia, Iceland, Slovakia, Slovenia, South Africa, Sweden, Netherlands), 20–59 (Australia, Costa Rica, Israel, Italy, Singapore, Spain), or 20–64 (Denmark, New Zealand, Uruguay); and senior refers to people aged over 60 or 65 years old. Some countries only reported data for 0–19-year-olds (Israel), which we designated as an adolescent group

Among 31 countries reporting vaccine administration data by age group, the percent of people that received full-schedules was highest among seniors (people over 60 or 65 years old) (range: 44.2–100.0%), followed by middle-aged adults (18–60 or 18–65 years old) (19.4–97.0%), adolescents (12–17 years old) (0.3–100.0%), and children (0–11 years old) (0.03–86.4%) (Fig. 4A). A similar pattern of vaccine distribution was also observed in one-or-more-dose coverage (Fig. 4B). Exceptionally, vaccination rates among seniors in some countries, such as China and Chile, were lower than that among adolescents and adults.

### Influencing factors of vaccine coverage

**Fig. 5:**
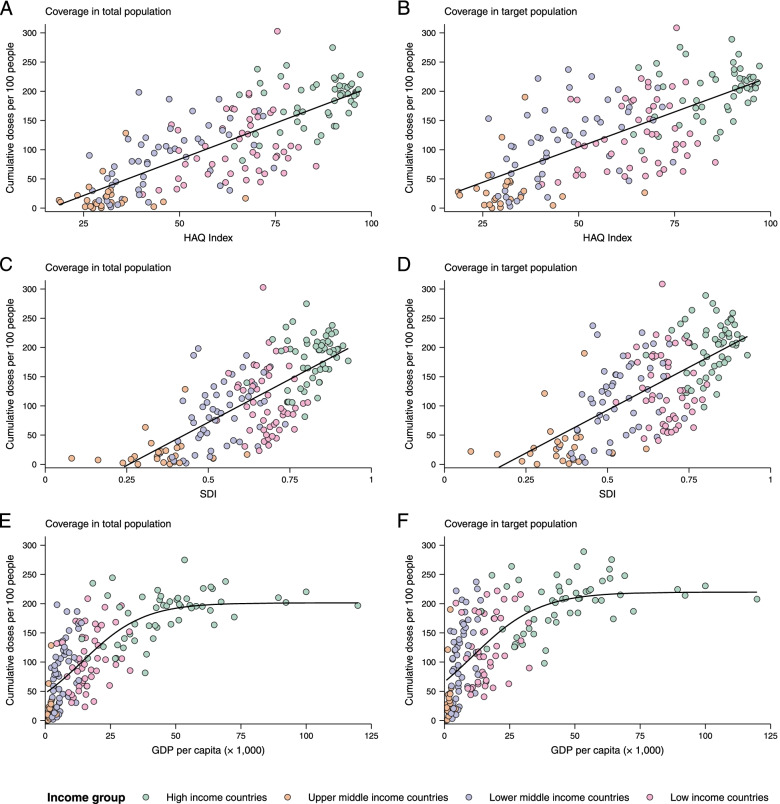
Correlations with vaccine coverage. Correlation of cumulative doses administered per 100 people among total populations or target populations with healthcare access and quality (HAQ) index (**A**, **B**), socio-demographic index (SDI) (**C**, **D**), GDP per capita (units: dollars) after adjusting for purchasing power parity (**E**, **F**). Solid lines show linear or nonlinear fit

Vaccine coverage was moderately associated with SDI, HAQ, and GDP per capita-PPP. Correlations between doses administered per 100 individuals in total population and the HAQ index (*R*^2^ = 0.59), SDI index (*R*^2^ = 0.52), and GDP per capita-PPP (*R*^2^ = 0.61) are shown in Fig. 5A, C, E; correlations per 100 persons in target population and HAQ index (*R*^2^ = 0.50), SDI index (*R*^2^ = 0.46), and GDP per capita-PPP (*R*^2^ = 0.51) are shown in Fig. 5B, D, F. In general, countries with higher socio-demographic or health resource-related levels had higher coverage. As GDP per capita-PPP increased to approximately 50,000 dollars, coverage plateaued (Fig. 5E, F). In addition, the vaccine coverage was also related with physician density and government health spending per capita (Additional file 1: Fig. S8).

### Demands of vaccine doses

The total estimated global demand was 6.4 billion doses to complete ongoing vaccination programs—3.3 billion for primary immunization and 3.1 billion for additional/booster programs. The highest demand occurred in South-East Asia (1.37 billion doses), followed by Western Pacific (1.35 billion doses), Africa (1.16 billion doses), the Americas (0.88 billion doses), Europe (0.86 billion doses), and Eastern Mediterranean (0.78 billion doses) (Additional file 1: Table S9).

**Fig. 6:**
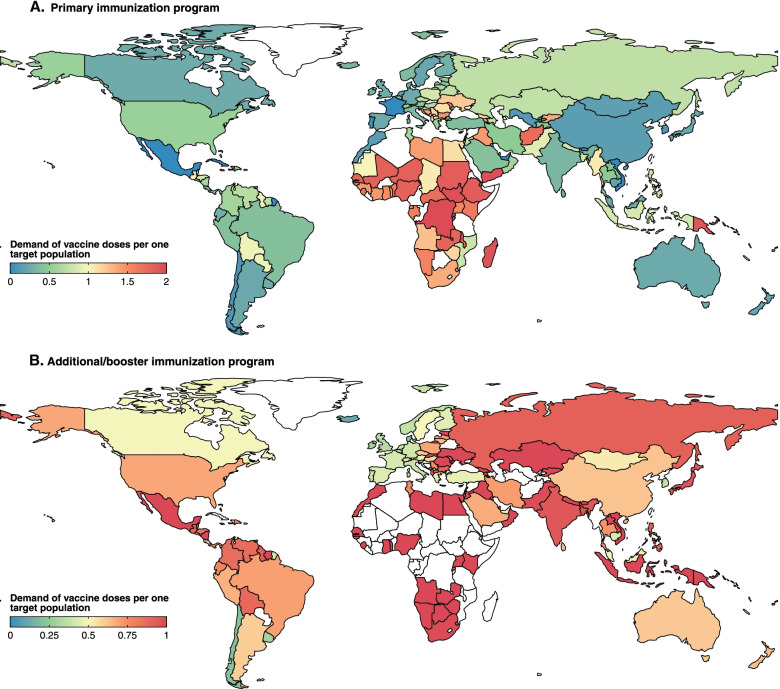
Current demand of vaccine dose per individual in the target population. **A** Demand of vaccine doses for primary immunization per individual in the target population. **B** Demand of vaccine dose for additional/booster immunization per individual in the target population. Target population is defined as those eligible for primary or additional/booster vaccination recommended by each country’s immunization policy

Global demand for primary immunization was 0.53 doses per individual in the target population, with regional-heterogeneity observed in the African (1.41), Eastern Mediterranean (0.88), European (0.50), South-East Asian (0.49), American (0.46), and Western Pacific (0.19) regions (Fig. 6A). Estimated vaccine dose demands at the country level were presented in the Additional file 1: Table S7. Additional and booster immunization policies increased demand to 0.74 per individual in the target population, as most additional/booster doses have not yet been administered to target populations for low- and middle-income countries (LMICs) (Fig. 6B).

## Discussion

Our study provided insight into the global landscape of COVID-19 vaccination policy, vaccine coverage, and demand of vaccines at this phase of vaccine rollout. Most countries have made COVID-19 vaccination a priority, and more than 10 billion doses of COVID-19 vaccines administered around the world represents a significant milestone in the response to the COVID-19 pandemic. With increases in vaccine production, the main target populations globally were groups aged 12 years and older, with some countries extending vaccination to children as young as 2 years old. However, we showed vast differences in vaccine coverage across countries, in which doses administered per capita in high-income countries was 11.9 times that of low-income countries. As a result, a huge imbalance in demand raises concerns of inequitable access to vaccines, even as production capacity increases.

Vaccination has shaped the epidemic curve of COVID-19, especially for severe outcomes [1]. However, we found a pattern similar to the dilemma of vaccine distribution during the 2009 H1N1 influenza pandemic in which high-income countries procured most vaccines, and access to vaccines remained inequitable [39, 40]. Although vulnerable groups have not been effectively protected in some resource-poor countries, countries with high vaccine coverage have initiated booster programs or even fourth doses based on the evidence that current COVID-19 vaccines provide sustained protection against severe outcomes and even variants of concern, but antibody levels have waned [41]. This may further widen the gap of population immunity between countries, as supply exceeded demand very early in high-income countries, with relatively little transfer of vaccines to LMICs. Disparities in coverage among countries may impede the global effort of building herd immunity to stop the pandemic. Slow vaccine rollouts in some low-income countries left individuals vulnerable to emerging variants, and the expanding epidemic may further increase risk of emergence of new SARS-CoV-2 variants [42]. Optimal allocation for limited supplies of vaccines to people at high risk or who have no immunity may save more lives and help contain the pandemic by building more immunity. Although COVAX planned to procure and deliver at least 2 billion doses by the end of 2021 [43], only 367 million doses have been successfully allocated as of August 2021 [31].

Scaling up production capacity of current vaccines and developing more effective vaccines remain top priorities, especially since concerns over waning immunity and SARS-CoV-2 variants have led some countries to deploy extra vaccine doses. It may be feasible to use a booster dose with less antigen as a dose-sparing strategy that still provides adequate immune response [44, 45]. With limited supplies of several vaccines, heterologous prime-boost regimens also should be considered because they appear to induce strong immune responses [46]. Research and development of one-dose, variant-specific, and broad-spectrum vaccines could curb the pandemic more effectively and efficiently. It is also vital to strengthen international coordination of development, manufacturing, and deployment—for example, by sharing knowledge and expertise, and providing useful guidance to build or improve production facility layouts and production lines [47], enabling more countries to make vaccines.

In addition to challenges of production, affordability, and allocation, vaccine hesitancy is a key barrier for some countries with sufficient supplies to reach the expected vaccine coverage levels among target populations (Additional file 1: Fig. S9) [48]. Since vaccine hesitancy is not a singular problem, interventions should be implemented that build and sustain vaccine confidence through joint efforts by vaccine manufacturers, governments, and other parties to ensure safety and effectiveness of vaccines and provide timely disclosure of relevant information to the public [49]. Although the varying degree of vaccine hesitancy among countries might have impacted our association analyses and estimates of demand, we believe the impact was relatively small since acceptance of COVID-19 vaccines will likely change over time as more robust evidence and monetary incentive policies emerge [50].

Our univariate analysis showed that socioeconomic and health system-related factors might be predictors of vaccine coverage. Countries with a higher SDI and HAQ score were more likely to have more vaccine doses administered than those with lower scores. This finding may reflect that healthcare systems play a key role in vaccine rollout, because health-related resources and capabilities are necessary for vaccination campaigns. For example, inadequate equipment for temperature control and time lags between shipments and deployment of vaccines may impede vaccination progress in low-income countries. However, the associated factors identified in our univariate analysis cannot be always interpreted as being naturally causal and the interaction of those factors are not addressed since these are complex processes that affect vaccination rollout and merit further investigations [6]. Arguably, widespread vaccine coverage needs to fit multiple efforts simultaneously—at global, national, and sub-national levels, including accessibility of vaccine, individual acceptance, and healthcare system requirements for vaccination [5, 51].

Planning COVID-19 vaccination programs is complex and arduous. Some concerns were raised during vaccination campaigns. Immunization strategy for individuals previously infected with SARS-CoV-2 remains unclear, while the number of confirmed cases worldwide has reached 400 million (as of 8 February 2022), accounting for 5.3% of the total global population [1]. Interim evidence suggests that antibody levels in naturally-infected people persist for over 1 year [52, 53]. Vaccine-induced immune responses in individuals with mild previous infection are generally higher [54], as previously infected individuals who were given one dose of a COVID-19 vaccine have higher responses than full-schedule vaccination of people who had not been previously infected [55, 56]. Therefore, a one-dose schedule for previously infected individuals might be acceptable to achieve the dual purpose of protecting populations and saving supplies. Certainly, more evidence is needed to guide future policies.

The emergence and global dominance of Omicron variant further highlights the importance of rapidly addressing the global vaccine disparities and inequality. COVAX would benefit from increasing cooperative actions facilitating access to more pre-qualified vaccines for participating countries. For LMIC populations that have not benefited from COVAX vaccines, the World Bank, wealthier countries, and other capable groups could provide more financial support for vaccine purchase or direct donation of vaccines. With these approaches, some countries have achieved relatively high vaccine coverage, including Indonesia and Bangladesh [57, 58]. The delivery of COVID-19 vaccines requires much preparation—for example establishing vaccination rollout platforms and infrastructure in LMICs [59]. Low-income countries can construct regional networks, apart from bilateral partnerships, to support ultracold chain requirements of mRNA COVID-19 vaccines and train healthcare system personnel to identify target population [60]. Most importantly, transferring vaccine technologies to LMIC-based manufacturers would be a long-term benefit to global equity. Such transfer needs waiver of intellectual property protections for COVID-19 vaccines and strengthening of regulatory capacity [60, 61].

There are several limitations to our study. First, all data in our study were obtained from public sources. Therefore untimely, opaque, and language-restricted data disclosure limited data completeness and prediction of subpopulation immunity. Standardizing reporting of COVID-19 vaccination and increasing data sharing and transparency could promote progress of the global vaccination campaign. Second, estimates of population sizes with contraindications and immunosuppressing conditions were constrained because it is difficult to determine what proportions of these populations could be vaccinated. Some countries use vaccines off label, further complicating determination of the eligible population. Third, country vaccination policies will change with more real-world evidence and experience; subnational units may make local vaccination policy independent of national recommendations. Vaccine demand in our study reflected only national level policies. Fourth, to provide a comprehensive landscape to the end of the study period, we included country data within two weeks of the end of the study period, introducing a small amount of error from missing end-period values. Fifth, our association analyses make it impossible to accurately measure the contribution of each factor, thereby further studies are needed to identify the explicit drivers of vaccination.

## Conclusions

Our full picture of COVID-19 vaccination policy, coverage, and current demand in an ongoing epidemic deepens the understanding of this unprecedent vaccination effort. Disparity and inequity of vaccination rollout worldwide implies that susceptibility of unvaccinated populations in some countries may impede or reverse pandemic control, especially in the face of Omicron and future variants. More countries and organizations should be involved in the global response to the pandemic, taking responsibility and providing leadership to overcome the complex challenges that lie ahead—financially, politically, and technically.

## Supplementary Information

**Additional file 1: Figure S1.** The distribution of whether local residents need to pay for vaccine. **Figure S2.** Geographic distribution of overall technical platforms for vaccines. **Figure S3.** Proportion administered by vaccine technical platforms. **Figure S4.** Proportion administered by vaccine types. **Figure S5.** Date at which achieved one dose per 100 people in total population by country. **Figure S6.** Vaccine coverage over time stratified by income groups and role of vaccine seller/donor or recipient. **Figure S7.** Vaccine coverage stratified by SDI quintile and WHO region. **Figure S8.** The association between vaccine coverage with physician density and government health spending per capita. **Figure S9.** Corrections between vaccine coverage and country-level vaccine acceptance. **Table S1.** Authorization information of COVID-19 vaccines by technical platforms and country. **Table S2.** Target populations and contraindications recommended by regulatory agencies. **Table S3.** Policies on additional or booster dose of COVID-19 vaccine. **Table S4.** Country lists of selling/donating or receiving COVID-19 vaccines. **Table S5.** Categories and definitions of special population groups belonging to indication and contraindication lists. **Table S6.** Global, regional, and national target population (TP). **Table S7.** The list of variables for investigating associations with vaccine coverage. **Table S8.** Analysis of multicollinearity. **Table S9.** Global, regional, and national demand of vaccine dose.

## Data Availability

Data used in this study have been presented within the manuscript and additional supporting files. Requests for other materials should be addressed to the corresponding author.

## Abbreviations

COVID-19: The coronavirus disease 2019
NPIs: Non-pharmaceutical interventions
SARS-CoV-2: Severe acute respiratory syndrome coronavirus-2
COVAX: COVID-19 Vaccine Global Access
GDP: Gross domestic product
WHO: World Health Organization
SDI: Socio-demographic index
HAQ: Healthcare access and quality
PPP: Purchasing power parity
VIF: Variance inflation factor
LMICs: Low- and middle-income countries
