# Supplementary material for: Global diversity of policy, coverage, and demand of COVID-19 vaccines: a descriptive study
Source: BMC Med. 2022 Apr 4;20:130. doi: 10.1186/s12916-022-02333-0 (PMC8977121; doi:10.1186/s12916-022-02333-0)
Supplement: Supplementary file 1 — Additional file 1: Figure S1. The distribution of whether local residents need to pay for vaccine. Figure S2. Geographic distribution of overall technical platforms for vaccines. Figure S3. Proportion administered by vaccine technical platforms. Figure S4. Proportion administered by vaccine types. Figure S5. Date at which achieved one dose per 100 people in total population by country. Figure S6. Vaccine coverage over time stratified by income groups and role of vaccine seller/donor or recipient. Figure S7. Vaccine coverage stratified by SDI quintile and WHO region. Figure S8. The association between vaccine coverage with physician density and government health spending per capita. Figure S9. Corrections between vaccine coverage and country-level vaccine acceptance. Table S1. Authorization information of COVID-19 vaccines by technical platforms and country. Table S2. Target populations and contraindications recommended by regulatory agencies. Table S3. Policies on additional or booster dose of COVID-19 vaccine. Table S4. Country lists of selling/donating or receiving COVID-19 vaccines. Table S5. Categories and definitions of special population groups belonging to indication and contraindication lists. Table S6. Global, regional, and national target population (TP). Table S7. The list of variables for investigating associations with vaccine coverage. Table S8. Analysis of multicollinearity. Table S9. Global, regional, and national demand of vaccine dose. [file 12916_2022_2333_MOESM1_ESM.docx]

**Additional file 1**

**Global diversity of policy, coverage, and demand of COVID-19 vaccines: a descriptive study**

Zhiyuan Chen^1†^, Wen Zheng^1†^, Qianhui Wu^1†^, Xinghui Chen^1†^, Cheng Peng^1†^, Yuyang Tian^1^, Ruijia Sun^1^, Jiayi Dong^1^, Minghan Wang^1^, Xiaoyu Zhou^1^, Zeyao Zhao^1^, Guangjie Zhong^1^, Xuemei Yan^1^, Nuolan Liu^1^, Feiran Hao^1^, Sihong Zhao^1^, Tingyu Zhuang^1^, Juan Yang^1,2^, Andrew S. Azman^3,4^, Hongjie Yu^1,2,5^

Author Affiliations:

1. School of Public Health, Fudan University, Key Laboratory of Public Health Safety, Ministry of Education, Shanghai, China
2. Shanghai Institute of Infectious Disease and Biosecurity, Fudan University, Shanghai, China
3. Department of Epidemiology, Johns Hopkins Bloomberg School of Public Health, Baltimore, MD, USA
4. Institute of Global Health, Faculty of Medicine, University of Geneva, Switzerland
5. Department of Infectious Diseases, Huashan Hospital, Fudan University, Shanghai, China

^†^These authors contributed equally to this work.

Corresponding authors: Hongjie Yu, School of Public Health, Fudan University, Shanghai 200032, China; E-mail: [yhj@fudan.edu.cn](mailto:yhj@fudan.edu.cn)

**Contents**

[COVID-19 vaccination policy 3](#_Toc96206297)

[*Metrics included in the dataset of COVID-19 vaccination policy* 3](#_Toc96206298)

[*Supplementary results* 4](#_Toc96206299)

[Table S1. Authorization information of COVID-19 vaccines by technical platforms and country. 4](#_Toc96206300)

[Table S2. Target populations and contraindications recommended by regulatory agencies 12](#_Toc96206301)

[Table S3. Policies on additional or booster dose of COVID-19 vaccine 42](#_Toc96206302)

[Table S4. Country lists of selling/donating or receiving COVID-19 vaccines 69](#_Toc96206303)

[Figure S1. The distribution of whether local residents need to pay for vaccine. 70](#_Toc96206304)

[Figure S2. Geographic distribution of overall technical platforms for vaccines 71](#_Toc96206305)

[Target population for primary vaccination 72](#_Toc96206306)

[*Method to estimate the size of target population* 72](#_Toc96206307)

[*Supplementary results* 73](#_Toc96206308)

[Table S5. Categories and definitions of special population groups belonging to indication and contraindication lists 73](#_Toc96206309)

[Table S6. Global, regional, and national target population (TP). 78](#_Toc96206310)

[COVID-19 vaccine coverage 85](#_Toc96206311)

[*Metrics included in the dataset of administered doses* 85](#_Toc96206312)

[*Supplementary results* 85](#_Toc96206313)

[Table S7. The list of variables for investigating associations with vaccine coverage 85](#_Toc96206314)

[Table S8. Analysis of multicollinearity 85](#_Toc96206315)

[Figure S3. Proportion administered by vaccine technical platforms 86](#_Toc96206316)

[Figure S4. Proportion administered by vaccine types. 87](#_Toc96206317)

[Figure S5. Date at which achieved one dose per 100 people in total population by country. 88](#_Toc96206318)

[Figure S6. Vaccine coverage over time stratified by income groups and role of vaccine seller/donor or recipient. 89](#_Toc96206319)

[Figure S7. Vaccine coverage stratified by SDI quintile and WHO region. 90](#_Toc96206320)

[Figure S8. The association between vaccine coverage with physician density and government health spending per capita. 91](#_Toc96206321)

[Figure S9. Corrections between vaccine coverage and country-level vaccine acceptance. 92](#_Toc96206322)

[Demand of COVID-19 vaccine doses 93](#_Toc96206323)

[*Method to calculate the demand of COVID-19 vaccine doses* 93](#_Toc96206324)

[Table S9. Global, regional, and national demand of vaccine dose. 94](#_Toc96206325)

[References 105](#_Toc96206326)

# COVID-19 vaccination policy

## *Metrics included in the dataset of COVID-19 vaccination policy*

(1) **Authorization status**. Status includes licensure in use, emergency use authorization, conditional marketing, special access route, and recipients of COVAX or other countries.

(2) **Indications and contraindications**. People who could and should not get vaccinated against COVD-19, recommended by governments/health departments.

(3) **Additional/booster dose policy**. If the government/health department gives a clear definition of an additional/booster dose, we adopted it. Otherwise, we used the definition by the United States Centers for Disease Control and Prevention (https://www.cdc.gov/coronavirus/2019-ncov/vaccines/booster-shot.html).

3.1 **Target population**. Individuals who are eligible for a COVID-19 vaccine additional/booster shot.

3.2 **Interval**. The time between the completion of primary immunization and additional/booster shots.

(4) **Whether local residents need to pay for vaccine.**

## *Supplementary results*

### Table S1. Authorization information of COVID-19 vaccines by technical platforms and country.

| **Vaccine (Trade name)** | **Authorization status** | **Use status** | **Country lists** |
| --- | --- | --- | --- |
| **Adenovirus vectored vaccine** | | | |
| 1. Convidecia | Conditional marketing | In use | China |
|  | Emergency Use Authorization | In use | Argentina, Chile, Ecuador, Indonesia, Malaysia, Mexico, Pakistan, Russian Federation |
| 1. Covishield | Conditional marketing | Currently not in use | Canada |
|  | Emergency Use Authorization | Currently not in use | South Africa |
|  | Emergency Use Authorization | In use | Argentina, Bahrain, Bangladesh, Barbados, Bhutan, Botswana, Brazil, Cambodia, Cameroon, Dominica, Egypt, Ghana, India, Iran, Lao People's Democratic Republic, Madagascar, Maldives, Mauritius, Mexico, Morocco, Nepal, Nicaragua, Nigeria, Nigeria, Oman, Republic of Korea, Republic of Moldova, Seychelles, Sri Lanka, Ukraine, Ukraine, Uzbekistan |
|  | Recipients of COVAX or other countries | In use | Afghanistan, Angola, Ethiopia, Grenada, Guinea, Lesotho, Liberia, Malawi, Mali, Mauritania, Papua New Guinea, Saint Kitts and Nevis, Somalia, Suriname, Syrian Arab Republic, Togo, Uganda, Yemen, Zambia, Trinidad and Tobago |
| 1. Janssen COVID-19 Vaccine | Conditional marketing | Currently not in use | Denmark, Finland, Norway, Sweden |
|  | Conditional marketing | In use | Austria, Belgium, Bulgaria, Canada, Croatia, Cyprus, Czechia, Estonia, France, Germany, Greece, Iceland, Ireland, Italy, Latvia, Lithuania, Luxembourg, Malta, Netherlands, Romania, Slovakia, Slovenia, Spain |
|  | Emergency Use Authorization | Currently not in use | Chile, India, Thailand |
|  | Emergency Use Authorization | In use | Antigua and Barbuda, Bahamas, Bangladesh, Belize, Bolivia, Botswana, Brazil, Brunei, Burkina Faso, Cambodia, Cameroon, Colombia, Cote d'Ivoire, Egypt, Ghana, Guinea, Hungary, Indonesia, Kenya, Kuwait, Lao People's Democratic Republic, Lesotho, Libya, Madagascar, Malaysia, Maldives, Marshall Islands, Mauritius, Mexico, Micronesia, Namibia, New Zealand, Niger, Nigeria, Niue, Oman, Philippines, Poland, Portugal, Republic of Korea, Republic of Moldova, Rwanda, Saudi Arabia, South Africa, Sudan, Switzerland, Togo, Tunisia, United Kingdom, United States, Viet Nam, Zambia, Zimbabwe, Trinidad and Tobago |
|  | Recipients of COVAX or other countries | In use | Afghanistan, Algeria, Angola, Benin, Central African Republic, Chad, Djibouti, Eswatini, Ethiopia, Gambia, Guyana, Haiti, Jamaica, Liberia, Malawi, Mali, Mauritania, Mozambique, Nepal, Palau, Papua New Guinea, Senegal, Sierra Leone, Somalia, South Sudan, Tajikistan, United Republic of Tanzania, Yemen |
| 1. Sputnik Light | Emergency Use Authorization | In use | Belarus, Mongolia, Saint Vincent and the Grenadines, San Marino, Venezuela |
|  | Licensure | In use | Russian Federation |
| 1. Sputnik V | Emergency Use Authorization | Currently not in use | Bangladesh, Maldives |
|  | Emergency Use Authorization | In use | Albania, Algeria, Argentina, Armenia, Azerbaijan, Bahrain, Belarus, Bolivia, Bosnia and Herzegovina, Ecuador, Egypt, Gabon, Ghana, Guatemala, Guinea, Guyana, Honduras, Hungary, India, Iran, Iraq, Jordan, Kazakhstan, Kyrgyzstan, Libya, Mauritius, Mexico, Mongolia, Montenegro, Myanmar, Namibia, Nicaragua, Nigeria, North Macedonia, Oman, Pakistan, Paraguay, Philippines, Republic of Moldova, Saint Vincent and the Grenadines, San Marino, Serbia, Seychelles, Slovakia, Sri Lanka, Syrian Arab Republic, Tunisia, Turkmenistan, United Arab Emirates, Uzbekistan, Venezuela, Viet Nam, Zimbabwe |
|  | Licensure | In use | Russian Federation |
|  | Recipients of COVAX or other countries | In use | Angola, Tajikistan |
| 1. Sputnik M | Licensure | In use | Russian Federation |
| 1. Vaxzevria | Conditional marketing | Currently not in use | Denmark, Norway, Sweden |
|  | Conditional marketing | In use | Austria, Belgium, Bulgaria, Canada, Croatia, Cyprus, Czechia, Estonia, Finland, France, Germany, Greece, Iceland, Ireland, Italy, Latvia, Lithuania, Luxembourg, Malta, Netherlands, Romania, Slovakia, Slovenia, Spain, Viet Nam |
|  | Emergency Use Authorization | In use | Albania, Andorra, Antigua and Barbuda, Argentina, Armenia, Australia, Azerbaijan, Bahamas, Belize, Benin, Bolivia, Bosnia and Herzegovina, Botswana, Brazil, Brunei, Burkina Faso, Cabo Verde, Cambodia, Chile, Colombia, Costa Rica, Cote d'Ivoire, Dominica, Dominican Republic, Ecuador, Egypt, El Salvador, Georgia, Guatemala, Guyana, Honduras, Hungary, Indonesia, Iraq, Israel, Japan, Kenya, Kuwait, Kyrgyzstan, Lebanon, Libya, Malaysia, Maldives, Mauritius, Mexico, Mongolia, Montenegro, Namibia, Nauru, New Zealand, Nicaragua, Niger, Niue, North Macedonia, Oman, Pakistan, Panama, Paraguay, Peru, Philippines, Poland, Portugal, Saint Lucia, Saint Vincent and the Grenadines, Samoa, Saudi Arabia, Serbia, Thailand, Tunisia, Turkmenistan, Ukraine, United Arab Emirates, United Kingdom, Uruguay |
|  | Recipients of COVAX or other countries | In use | Afghanistan, Algeria, Central African Republic, Democratic Republic of the Congo, Djibouti, Eswatini, Ethiopia, Fiji, Gambia, Guinea-Bissau, Jamaica, Jordan, Kiribati, Malawi, Mauritania, Mozambique, Myanmar, Papua New Guinea, Republic of the Congo, Rwanda, Sao Tome and Principe, Senegal, Sierra Leone, Solomon Islands, Somalia, South Sudan, Sudan, Tajikistan, Timor-Leste, Togo, Tonga, Tuvalu, Vanuatu, Zambia |
| **Conjugate vaccine** | | | |
| 1. Soberana 02 | Emergency Use Authorization | In use | Cuba, Iran, Nicaragua |
| 1. Soberana Plus | Emergency Use Authorization | In use | Cuba |
| **DNA vaccine** | | | |
| 1. ZyCoV-D | Emergency Use Authorization | Currently not in use | India |
| **Inactivated vaccine** | | | |
| 1. BBIBP-CorV | Conditional marketing | In use | China |
|  | Emergency Use Authorization | In use | Antigua and Barbuda, Argentina, Armenia, Bahrain, Bangladesh, Barbados, Belarus, Belize, Bolivia, Brunei, Cambodia, Cameroon, Comoros, Dominica, Dominican Republic, Egypt, Equatorial Guinea, Gabon, Gambia, Georgia, Guyana, Hungary, Indonesia, Iran, Iraq, Jordan, Kenya, Kyrgyzstan, Lao People's Democratic Republic, Lao People's Democratic Republic, Lesotho, Madagascar, Maldives, Mauritania, Mauritius, Mongolia, Montenegro, Morocco, Namibia, Nepal, Niger, Nigeria, North Macedonia, Oman, Pakistan, Paraguay, Peru, Philippines, Republic of Moldova, Republic of the Congo, Saudi Arabia, Senegal, Serbia, Seychelles, Sri Lanka, Suriname, Thailand, United Arab Emirates, Venezuela, Viet Nam, Zimbabwe, Trinidad and Tobago |
|  | Recipients of COVAX or other countries | In use | Afghanistan, Angola, Bosnia and Herzegovina, Burundi, Chad, Cote d'Ivoire, Ethiopia, Guinea-Bissau, Jamaica, Kazakhstan, Kiribati, Lebanon, Mali, Mozambique, Myanmar, Nicaragua, Papua New Guinea, Rwanda, Sierra Leone, Solomon Islands, Somalia, Sudan, Turkmenistan, United Republic of Tanzania, Vanuatu, Zambia |
|  | Special access route | In use | Singapore |
| 1. CoronaVac | Conditional marketing | In use | China |
|  | Emergency Use Authorization | Currently not in use | Panama |
|  | Emergency Use Authorization | In use | Albania, Azerbaijan, Bangladesh, Benin, Bosnia and Herzegovina, Botswana, Cambodia, Chile, Colombia, Djibouti, Dominican Republic, Ecuador, Egypt, El Salvador, Equatorial Guinea, Georgia, Guinea, Indonesia, Indonesia, Kazakhstan, Malaysia, Mexico, Nepal, North Macedonia, Oman, Pakistan, Paraguay, Philippines, Singapore, Thailand, Tunisia, Turkey, Ukraine, Uruguay, Venezuela, Zimbabwe |
|  | Licensure | In use | Brazil |
|  | Recipients of COVAX or other countries | In use | Algeria, Armenia, Libya, Mali, Myanmar, Republic of Moldova, Syrian Arab Republic, Tajikistan, Timor-Leste, Togo, Turkmenistan, Uganda, Yemen |
| 1. Covaxin | Emergency Use Authorization | Currently not in use | Mexico |
|  | Emergency Use Authorization | In use | Botswana, Guyana, India, Iran, Mauritius, Nepal, Paraguay, Philippines, Zimbabwe |
| 1. Covidful | Emergency Use Authorization | In use | China |
| 1. COVIran Barekat | Emergency Use Authorization | In use | Iran |
| 1. CoviVac | Licensure | In use | Russian Federation |
| 1. FAKHRAVAC | Emergency Use Authorization | In use | Iran |
| 1. KCONVAC | Emergency Use Authorization | In use | China |
| 1. QazVac | Emergency Use Authorization | In use | Kazakhstan, Kyrgyzstan |
| 1. WBIP-CorV | Conditional marketing | In use | China |
| 1. Turkovac | Emergency Use Authorization | In use | Turkey |
| **mRNA vaccine** | | | |
| 1. Comirnaty | Conditional marketing | In use | Austria, Belgium, Bulgaria, Canada, Croatia, Cyprus, Czechia, Denmark, Estonia, Finland, France, Germany, Greece, Iceland, Ireland, Italy, Latvia, Lithuania, Luxembourg, Malta, Monaco, Netherlands, Republic of Korea, Romania, San Marino, Slovakia, Slovenia, Spain, Sweden |
|  | Emergency Use Authorization | In use | Albania, Andorra, Argentina, Armenia, Australia, Azerbaijan, Bahamas, Bahrain, Bangladesh, Barbados, Belize, Bhutan, Bolivia, Bosnia and Herzegovina, Botswana, Brazil, Brunei, Cabo Verde, Cambodia, Cameroon, Chile, Colombia, Comoros, Cook Islands, Costa Rica, Democratic Republic of the Congo, Dominican Republic, Ecuador, Egypt, El Salvador, Gabon, Ghana, Georgia, Guatemala, Guinea, Honduras, Hungary, Indonesia, Iraq, Israel, Jamaica, Japan, Jordan, Kazakhstan, Kenya, Kuwait, Lao People's Democratic Republic, Lebanon, Lesotho, Libya, Madagascar, Malaysia, Maldives, Marshall Islands, Mauritania, Mexico, Micronesia, Mongolia, Montenegro, Morocco, Namibia, Nepal, New Zealand, Niger, Nigeria, Niue, North Macedonia, Norway, Oman, Pakistan, Palau, Panama, Paraguay, Peru, Philippines, Poland, Portugal, Qatar, Republic of Moldova, Saint Kitts and Nevis, Saint Lucia, Saint Vincent and the Grenadines, Serbia, Seychelles, Singapore, South Africa, Sri Lanka, Suriname, Thailand, Tunisia, Turkey, Ukraine, United Arab Emirates, United Kingdom, United States, Uruguay, Viet Nam, Trinidad and Tobago |
|  | Licensure | In use | Saudi Arabia, Switzerland |
|  | Recipients of COVAX or other countries | In use | Angola, Antigua and Barbuda, Benin, Chad, Cote d'Ivoire, Dominica, Eswatini, Ethiopia, Fiji, Grenada, Guyana, Malawi, Mali, Mauritius, Nauru, Rwanda, Samoa, Sierra Leone, Solomon Islands, Sudan, Tajikistan, Timor-Leste, Togo, Tonga, Uganda, Uzbekistan, Zambia |
| 1. Spikevax | Conditional marketing | In use | Austria, Belgium, Bulgaria, Canada, Croatia, Cyprus, Czechia, Denmark, Estonia, Finland, France, Germany, Greece, Iceland, Ireland, Italy, Latvia, Lithuania, Luxembourg, Malta, Netherlands, Romania, San Marino, Slovakia, Slovenia, Spain, Sweden |
|  | Emergency Use Authorization | Currently not in use | India |
|  | Emergency Use Authorization | In use | Argentina, Australia, Botswana, Brunei, Cambodia, Colombia, Democratic Republic of the Congo, Guatemala, Guinea, Honduras, Hungary, Indonesia, Israel, Japan, Kenya, Kuwait, Libya, Maldives, Marshall Islands, Mexico, Micronesia, Mongolia, Nepal, Nigeria, Norway, Oman, Pakistan, Paraguay, Philippines, Poland, Portugal, Qatar, Republic of Korea, Republic of Moldova, Saudi Arabia, Singapore, Sri Lanka, Suriname, Switzerland, Thailand, Ukraine, United Arab Emirates, United Kingdom, United States, Uzbekistan, Viet Nam |
|  | Recipients of COVAX or other countries | In use | Fiji, Guyana, Haiti, Montenegro, Palau, Republic of the Congo, Rwanda, Sao Tome and Principe, Tajikistan, Tunisia, Uganda, Zambia |
| **Protein subunit vaccine** | | | |
| 1. Abdala | Emergency Use Authorization | In use | Cuba, Nicaragua, Saint Vincent and the Grenadines, Venezuela, Viet Nam |
| 1. EpiVacCorona | Emergency Use Authorization | In use | Turkmenistan |
|  | Licensure | In use | Russian Federation |
|  | Recipients of COVAX or other countries | In use | Belarus |
| 1. EpiVakKorona-N | Licensure | In use | Russian Federation |
| 1. Nuvaxovid | Conditional marketing | Currently not in use | Austria, Belgium, Bulgaria, Czechia, Estonia, France, Italy, Sweden |
|  | Emergency Use Authorization | Currently not in use | Australia, Indonesia, Republic of Korea |
| 1. Zifivax | Emergency Use Authorization | In use | China, Indonesia, Uzbekistan |

### Table S2. Target populations and contraindications recommended by regulatory agencies

| **Country** | **Target population** | **Contraindicated population** | **Data source** |
| --- | --- | --- | --- |
| **Africa** | | | |
| Algeria | 18+ | No data | http://www.xinhuanet.com/english/2020-12/21/c_139605514.htm  http://www.news.cn/english/2021-09/05/c_1310168528.htm |
| Angola | 12+ | No data | https://www.theeastafrican.co.ke/tea/rest-of-africa/angola-to-start-vaccinating-children-and-teenagers-3653532  https://newsaf.cgtn.com/news/2021-12-16/Angola-to-expand-COVID-19-vaccination-to-reach-12-year-olds-161ntZI5Epq/index.html |
| Benin | 12+ | No data | http://french.peopledaily.com.cn/Afrique/n3/2021/1013/c96852-9906467.html |
| Botswana | 12+ | No data | <https://fenyacovid.gov.bw/armready/> |
| Burkina Faso | 18+ | No data | https://www.rfi.fr/fr/afrique/20211221-le-burkina-faso-redouble-d-efforts-pour-vacciner-la-population |
| Burundi | 18+ | No data | https://reliefweb.int/sites/reliefweb.int/files/resources/burundi_covid-19_rapport_de_situation_2022-02-10_0.pdf |
| Cabo Verde | 12+ | No data | <https://reliefweb.int/report/cabo-verde/new-world-bank-support-equitable-access-covid-19-vaccines-cabo-verde>  https://covid19.cv/wp-content/uploads/2021/12/Vaccination-Bulletin-No-22_Ingles.pdf |
| Cameroon | 12+ | No data | https://www.gov.uk/foreign-travel-advice/cameroon/coronavirus  https://www.stopblablacam.com/society/0812-7891-cameroon-to-inoculate-12-15-year-olds-with-pfizer-vaccine |
| Central African Republic | 18+ | No data | https://www.africanews.com/2021/05/21/car-launches-covid-19-vaccination-in-bangui// |
| Chad | 18+ | No data | https://www.facebook.com/permalink.php?story_fbid=1182259412221131&id=586855375094874 |
| Comoros | 12+ | No data | https://www.facebook.com/permalink.php?story_fbid=311305667684521&id=100064152692176 |
| Cote d’Ivoire | 18+ | No data | <https://www.sante.gouv.ci/welcome/actualites/1192> |
| Democratic Republic of the Congo | 18+ | No data | https://actualite.cd/2021/12/15/covid-en-rdc-la-campagne-de-vaccination-volontaire-lancee-en-ituri |
| Equatorial Guinea | 18+ | No data | https://gq.usembassy.gov/covid-19-information/ |
| Eritrea | No data | No data |  |
| Eswatini | 12+ | No data | <https://twitter.com/eswatinigovern1/status/1445737600732459017?lang=ar-x-fm> |
| Ethiopia | 12+ | People with a history of anaphylaxis to any component of the vaccine or an anaphylactic reaction following the first dose of this vaccine | https://www.ephi.gov.et/images/novel_coronavirus/EPHI_PHEOC_COVID-19_Weekly_Bulletin_45_English_03132021.pdf  https://www.afro.who.int/news/ethiopia-launches-covid-19-vaccination-campaign-targeting-12-years-and-above-population |
| Gabon | 18+ | No data | http://www.xinhuanet.com/english/africa/2021-08/20/c_1310137345.htm |
| Gambia | 18+ | No data | https://www.facebook.com/Covid19GOUVGA/posts/382264960050625 |
| Ghana | 15+ | No data | https://www.voanews.com/a/us-donates-560-000-vaccine-doses-to-ghana/6423984.html |
| Guinea | 12+ | No data | https://www.reuters.com/world/africa/guinea-starts-vaccinating-children-against-covid-19-with-pfizer-moderna-2021-11-10/ |
| Guinea-Bissau | 12+ | No data | https://guyanachronicle.com/2021/08/26/vaccination-of-children-commences-today/ |
| Kenya | 15+ | No data | https://www.bbc.com/news/world-africa-59367726 |
| Lesotho | 12+ | No data | https://www.gov.ls/pfizer-vaccine-to-be-rolled-out/ |
| Liberia | 18+ | No data | https://frontpageafricaonline.com/health/crusaders-for-peace-unicef-partner-to-end-covid-19-through-rapid-immunization/  https://lr.usembassy.gov/wp-content/uploads/sites/53/COVID-19-Vaccine-Fact-Sheet_FINAL_3Aug21.pdf |
| Madagascar | 18+ | No data | https://mg.usembassy.gov/u-s-citizen-services/security-and-travel-information/covid-19-information/ |
| Malawi | 12+ | No data | https://reliefweb.int/report/malawi/situation-coronavirus-pandemic-country-and-review-rules-and-guidelines-context |
| Mali | 18+ | No data | http://www.sante.gov.ml/index.php/actualites/communiques/item/6341-lutte-contre-la-covid-19-au-mali-835-200-doses-de-sinovac-pour-poursuivre-la-campagne-de-vaccination |
| Mauritania | 12+ | No data | https://www.facebook.com/UNICEFMauritanie/posts/3009669819275108 |
| Mauritius | 12+ | No data | https://health.govmu.org/Documents/Main%20Page/Corona/775_New%20Registration%20and%20Consent%20Form%20Covid-19%20Vaccination.pdf |
| Mozambique | 15+ | Pregnant women | <https://covid19.ins.gov.mz/vacina-covid-19/>  https://covid19.ins.gov.mz/primeiro-ministro-lancou-esta-sexta-feira-o-plano-nacional-de-vacinacao/ |
| Namibia | 12+ | No data | http://www.news.cn/english/africa/2021-11/12/c_1310307551.htm |
| Niger | 18+ | No data | https://ne.usembassy.gov/u-s-citizen-services/covid-19-information/ |
| Nigeria | 16+ | People with allergic reactions to vaccine component | https://nphcda.gov.ng/faqs/ |
| Republic of the Congo | 18+ | No data | https://cd.usembassy.gov/covid-19-information/ |
| Rwanda | 12+ | No data | https://www.rbc.gov.rw/index.php?id=777 |
| Sao Tome and Principe | 12+ | No data | https://www.vaticannews.va/pt/africa/news/2022-01/sao-tome-e-principe-decorre-no-pais-campanha-de-vacinacao-de-ad.html |
| Senegal | 12+ | No data | https://www.reuters.com/world/africa/senegal-authorizes-covid-19-booster-shots-vaccines-children-2022-01-13/ |
| Seychelles | 12+ | No data | https://allafrica.com/stories/202108270089.html  http://www.health.gov.sc/index.php/covid-19-vaccination-faqs/ |
| Sierra Leone | 18+ | No data | https://dhse.gov.sl/wp-content/uploads/2021/09/PUBLIC-NOTICE-covid-113092021-1-1.pdf |
| South Africa | 12+ | People with a history of severe allergic reaction to any ingredient in the vaccine; people who is allergic to polyethene glycol (PEG) should not get the Pfizer vaccine, as it is one of the components; people who had a severe allergic reaction after the first dose should not get the second dose of that vaccine | https://www.sahpra.org.za/wp-content/uploads/2021/04/SAHPRA-Registration-with-conditions-for-the-Covid-19-vaccine-Janssen-Media-release-statement_final.pdf  https://www.iol.co.za/sunday-tribune/news/children-over-12-now-eligible-to-receive-covid-vaccine-58327f81-c0a3-49de-b4b0-097c605fa3aa  https://www.gov.za/coronavirus/faqs/vaccine |
| South Sudan | 18+ | No data | https://www.afro.who.int/news/south-sudan-receives-first-batch-covid-19-vaccines-through-covax-facility  https://www.afro.who.int/news/south-sudan-receives-its-first-consignment-johnson-johnson-covid-19-vaccines-through-covax |
| Tanzania | 18+ | No data | https://www.afro.who.int/news/united-republic-tanzania-receives-first-covax-shipment  https://tz.usembassy.gov/covid-19-information/ |
| Togo | 12+ | No data | https://www.republicoftogo.com/toutes-les-rubriques/sante/jeune-et-deja-vaccine |
| Uganda | 12+ | No data | http://www.xinhuanet.com/english/africa/2021-07/26/c_1310086931.htm |
| Zambia | 12+ | No data | https://www.moh.gov.zm/?p=6799 |
| Zimbabwe | 14+ | No data | https://www.aa.com.tr/en/africa/zimbabwe-free-vaccination-to-begin-thursday/2147237  https://apnews.com/article/lifestyle-africa-health-zimbabwe-coronavirus-pandemic-3e0b007ccccf08ae285710250daa84be |
| **Americas** | | | |
| Antigua and Barbuda | 12+ | Pregnant / breastfeeding women; persons who have severe allergic reactions to ingredients of the vaccine | https://www.facebook.com/investingforwellness/posts/1262969224143049  https://vaccineantiguabarbuda.com/faq/ |
| Argentina | 3+ | People with an allergic reaction to vaccine components or first dose; an acute SARS-CoV-2 infection; pre-vaccination pregnancy | https://www.argentina.gob.ar/coronavirus/vacuna/preguntas-frecuentes#7  https://www.argentina.gob.ar/coronavirus/vacuna/preguntas-frecuentes#22  https://www.argentina.gob.ar/coronavirus/vacuna/preguntas-frecuentes#20 |
| Bahamas | 12+ | No data | https://www.bahamas.gov.bs/wps/portal/public  https://vax.gov.bs/ |
| Barbados | 12+ | No data | https://gisbarbados.gov.bb/blog/pfizer-vaccine-now-available-to-the-general-public/ |
| Belize | 12+ | No data | https://www.facebook.com/Belizehealth/posts/2767419013556496 |
| Bolivia | 5+ | No data | <https://www.unidoscontraelcovid.gob.bo/index.php/vacunas/>  https://www.unidoscontraelcovid.gob.bo/index.php/2022/02/14/reporte-vacunados-14-2-2022/ |
| Brazil | 5+ | People with allergic action to the first dose or any vaccine component | <https://translate.googleusercontent.com/translate_f#21>  https://www.reuters.com/world/americas/pfizer-apply-covid-vaccine-use-brazil-children-5-11-years-2021-11-09/ |
| Canada | 5+ | No data | <https://www.canada.ca/en/public-health/services/diseases/coronavirus-disease-covid-19/vaccines/how-vaccinated.html>  https://www.canada.ca/en/public-health/services/vaccination-children/covid-19.html |
| Chile | 6+ | People with a known history of severe acute allergy (anaphylaxis) | <https://www.gob.cl/yomevacuno/>  <https://www.gob.cl/yomevacuno/preguntasfrecuentes/> |
| Colombia | 5+ | No data | https://www.como.gov/covidvaccine/#elementor-toc__heading-anchor-5  https://www.como.gov/covidvaccine/ |
| Costa Rica | 5+ | No data | https://www.ccss.sa.cr/arc/covid19/Manual_procedimientos_vacunacion_COVID.pdf |
| Cuba | 2+ | No data | https://salud.msp.gob.cu/actualizacion-de-la-vacunacion-en-el-marco-de-los-estudios-de-los-candidatos-vacunales-cubanos-y-la-intervencion-sanitaria/  <https://salud.msp.gob.cu/actualizacion-de-la-vacunacion-en-el-marco-de-los-estudios-de-los-candidatos-vacunales-cubanos-y-la-intervencion-sanitaria/> |
| Dominica | 12+ | No data | http://news.gov.dm/news/5337-pfizer-vaccines-arrive-in-dominica |
| Dominican Republic | 12+ | No data | <https://covid-19pharmacovigilance.paho.org/pfizer-biontech> |
| Ecuador | 12+ | No data | <https://ec.usembassy.gov/covid-19-information-ecu-2/>  <https://www.coronavirusecuador.com/2021/09/inicio-vacunacion-a-poblacion-de-12-a-15-anos-en-coordinacion-entre-los-ministerios-de-salud-y-educacion/> |
| El Salvador | 6+ | No data | https://www.presidencia.gob.sv/la-poblacion-meta-a-vacunar-contra-covid-19-incremento-a-5-9-millones-con-la-inclusion-de-los-nuevos-grupos-etarios/ |
| Grenada | 12+ | People with a severe allergic reaction to any ingredient in a COVID-19 vaccine | https://covid19.gov.gd/faq-vaccination/  https://caribbean.loopnews.com/content/grenada-begins-its-pfizer-vaccine-roll-out-soon |
| Guatemala | 12+ | People with a fever; people with serious illness; pregnant women | https://www.mspas.gob.gt/component/jdownloads/category/890-plan-nacional-de-vacunaci%C3%B3n-contra-la-covid-19.html?Itemid=-1  https://24newsrecorder.com/world/78123 |
| Guyana | 5+ | No data | https://www.health.gov.gy/  https://guyanachronicle.com/2021/11/14/children-5-11-to-get-covid-vaccine-from-nov-19/ |
| Haiti | 18+ | No data | https://www.paho.org/fr/haiti/vaccination-contre-covid-19-information-pratiques  https://ayibopost.com/haiti-en-pleine-campagne-de-vaccination/ |
| Honduras | 10+ | People with allergic reaction to the first dose or vaccine component; people with a fever | http://www.salud.gob.hn/site/index.php/component/edocman/resumen-ejecutivo-ampliacion-vii-campana-de-vacunacion-covid-19-v0709  http://www.salud.gob.hn/site/index.php/component/k2/item/2457-la-secretaria-de-salud-amplio-la-vacunacion-contra-el-covid-19-a-menores-de-10-anos |
| Jamaica | 12+ | No data | <https://vaccination.moh.gov.jm/frequently-asked-questions/>  https://www.moh.gov.jm/jamaica-opens-booster-programme-to-all/ |
| Mexico | 12+ | People with allergic reaction to vaccine component; people received blood transfusion or monoclonal antibodies | http://vacunacovid.gob.mx/wordpress/preguntas-frecuentes/ |
| Nicaragua | 2+ | No data | https://twitter.com/CraptnFreedom/status/1452853883663884288 |
| Panama | 5+ | No data | http://www.minsa.gob.pa/noticia/mas-de-31800-dosis-de-refuerzo-se-han-administrado-en-los-santos |
| Paraguay | 5+ | No data | https://www.vacunate.gov.py/index-documentos-tecnicos.html |
| Peru | 5+ | No data | https://doh.gov.ph/vaccines/know-your-vaccines  https://www.gob.pe/pongoelhombro#contador-de-vacunados |
| Saint Kitts and Nevis | 12+ | People who are allergic to substances in the vaccine; pregnant and breastfeeding women | https://covid19.gov.kn/covid-19-vaccination/  https://www.sknis.gov.kn/2021/08/13/pfizer-covid-19-vaccines-will-offer-protection-to-the-federations-children-12-years-and-up-minister-of-health-byron-nisbett/ |
| Saint Lucia | 12+ | No data | https://suntci.com/people-in-st-lucia-can-now-register-online-for-covid-vaccine-p5886-135.htm  http://socialtransformation.govt.lc/news/covid-19-vaccination-update2 |
| Saint Vincent and the Grenadines | 5+ | No data | https://www.facebook.com/permalink.php?story_fbid=5232397083487339&id=206099429450488 |
| Suriname | 12+ | People with proven severe allergies to any of the components of the vaccine; pregnant women | https://laatjevaccineren.sr/  https://laatjevaccineren.sr/vragen/#vaccineren |
| United States | 5+ | People with allergic reactions to vaccine component | https://www.cdc.gov/coronavirus/2019-ncov/vaccines/recommendations/specific-groups.html |
| Uruguay | 5+ | People with allergic reactions to the first dose or vaccine component | https://www.gub.uy/ministerio-salud-publica/comunicacion/publicaciones/preguntas-frecuentes-vacunacion-covid-19/sobre-vacunas/casos-indicados  https://www.gub.uy/uruguaysevacuna |
| Venezuela | 2+ | No data | https://america.cgtn.com/2021/12/03/venezuela-rolls-out-covid-19-vaccination-program-for-kids-aged-2-11 |
| Trinidad and Tobago | 12+ | People with allergic reactions to vaccine component | <https://health.gov.tt/covid-19/covid-19-vaccine/faqs> |
| **Eastern Mediterranean** | | | |
| Afghanistan | 18+ | No data | https://www.adb.org/sites/default/files/linked-documents/55012-001-sd-03.pdf |
| Bahrain | 12+; 3-11 with respiratory diseases, heart diseases, diabetes mellitus, obesity, cancer, Down syndrome and birth defects | People should not have allergies to any of the vaccine's components; women should not be pregnant or planning on getting pregnant, or lactating women | https://healthalert.gov.bh/uploads/dhep3ew1_l2l.pdf  <https://healthalert.gov.bh/uploads/sf3iuypu_oby.pdf>  <https://www.cvdvaccine-bh.com/files/V6_6-2-Final-121120-EUA_Full-Prescribing-Info_HCP-Fact-Sheet-Pfizer-BioNTech-COVID-19-vaccine-14-1-2021.pdf>  <https://www.nhra.bh/Media/Announcement/MediaHandler/GenericHandler/documents/Announcements/NHRA_News_MoH%20Circular_Inactivated%20COVID19%20Vaccine%20(Snopharm)_20201215n.pdf> |
| Djibouti | 18+ | No data | https://www.aa.com.tr/en/africa/djibouti-receives-more-covid-19-jabs-as-vaccination-drive-underway/2307470 |
| Egypt | 12+ | No data | http://www.news.cn/english/2021-12/16/c_1310377133.htm |
| Iran | 5+ | No data | https://www.aa.com.tr/en/latest-on-coronavirus-outbreak/iran-approves-vaccination-of-children-aged-5-11/2482532 |
| Iraq | 12+ | No data | <https://www2.hse.ie/screening-and-vaccinations/covid-19-vaccine/get-the-vaccine/deciding-on-vaccination-for-12-to-15-year-olds/> |
| Jordan | 12+ | People with allergic reactions to vaccine component, first dose, another vaccine; pregnant or breastfeeding women | <https://corona.moh.gov.jo/ar/page/1061/CoronaVaccineQuestions> |
| Kuwait | 5+ | No data | http://www.xinhuanet.com/english/20220130/9465f13875974ea48786ca146246d3fc/c.html |
| Lebanon | 5+ | People with an anaphylactic shock after receiving a previous dose of this vaccine, or a severe allergic reaction to any component of this vaccine | https://www.familyfirsthealth.org/lebanon-covid-19-vaccine-clinic  <https://www.moph.gov.lb/userfiles/files/Prevention/COVID-19%20Vaccine/COVID-19%20Vaccine%20FAQ%20-%20EN%20-22-3-%202021.pdf>  https://www.moph.gov.lb/userfiles/files/AwarenessCampaign/National%20Awareness%20Campaign%20on%20COVID%2019%20Vaccine%202021/COVID-19%20Vaccine%20FAQ%20-%20EN%20-%2008_02_2022.pd |
| Libya | 12+ | Pregnant and breastfeeding women | https://www.eservices.ly/  https://www.libyaobserver.ly/inbrief/ncdc-covid-19-vaccination-campaign-targets-age-group-12-17 |
| Morocco | 12+ | People with allergic reactions to vaccine component or the first dose; people allergic to other vaccine; pregnant or breastfeeding women; people with an infectious disease in the acute phase | https://www.africanews.com/2021/09/01/covid-19-morocco-begins-inoculating-children-over-12-years-old//  <https://liqahcorona.ma/fr/page-je-minforme-sur-le-vaccin#209>  https://liqahcorona.ma/fr/questions#faq |
| Oman | 5+ | No data | https://om.usembassy.gov/u-s-citizen-services/covid-19-information/  https://www.omanobserver.om/article/1108826/oman/health/children-above-5-years-to-get-vaccine-in-oman |
| Pakistan | 12+ | No data | https://ncoc.gov.pk/covid-vaccination-en.php |
| Qatar | 5+ | People with allergic reactions to vaccine component; pregnant women | https://covid19.moph.gov.qa/EN/Covid19-Vaccine/Pages/FAQ.aspx  https://www.moph.gov.qa/english/mediacenter/News/Pages/NewsDetails.aspx?ItemId=46 |
| Saudi Arabia | 5+ | People seriously infected with SARS-CoV-2 | <https://www.moh.gov.sa/en/eServices/Pages/Covid19-egistration.aspx>  https://articlesen.covid19awareness.sa/COVID-19-Vaccine-FAQs  https://saudigazette.com.sa/article/615900/SAUDI-ARABIA/Saudi-Arabia-expands-COVID-19-vaccinations-to-children-aged-5-11 |
| Somalia | 18+ | No data | https://reliefweb.int/report/somalia/race-against-time-boost-covid-19-vaccine-uptake-somalia |
| Sudan | 18+ | People with allergic reaction to the first dose; people with a fever | https://www.facebook.com/FMOH.SUDAN/posts/2986575494948742  https://www.unicef.org/sudan/stories/covid-19-vaccination-sudan |
| Syria | 18+ | No data | http://www.xinhuanet.com/english/2021-05/06/c_139926698.htm  http://www.emro.who.int/syria/news/update-on-covid-19-vaccination-in-syria-22-september-2021.html |
| Tunisia | 18+ | No data | https://reliefweb.int/report/tunisia/tunisia-receives-first-batch-covid-19-vaccines-through-covax-facility  https://reliefweb.int/report/tunisia/tunisia-receives-first-batch-covid-19-vaccines-through-covax-facility |
| United Arab Emirates | 3+ | People with allergic reactions to vaccine component; pregnant women | https://u.ae/en/information-and-services/justice-safety-and-the-law/handling-the-covid-19-outbreak/vaccines-against-covid-19-in-the-uae.  https://www.wam.ae/en/details/1395302961623 |
| Yemen | 18+ | No data | https://www.thenationalnews.com/gulf-news/yemenis-urged-to-take-covid-19-vaccine-as-first-astrazeneca-shipment-arrives-1.1194914 |
| **Europe** | | | |
| Albania | 16+; 12+ with immunosuppression | No data | http://shendetesia.gov.al/fushata-e-vaksinimit-shqiperia-buzeqesh/  https://shendetesia.gov.al/vaksina-anticovid-pyetje-pergjigje/ |
| Andorra | 12+ | No data | <https://www.salut.ad/preinscripcio-vacuna-covid19/> |
| Armenia | 12+ | If you have a history of severe allergic reactions to any ingredients of a COVID-19 vaccine; if you are currently sick or experiencing symptoms of COVID-19. | https://www.unicef.org/armenia/en/stories/everything-you-need-know-about-covid-19-vaccination-armenia#Question1  https://www.azatutyun.am/a/31535627.html |
| Austria | 5+ | Seriously allergic to first dose | https://www.sozialministerium.at/Corona-Schutzimpfung/Corona-Schutzimpfung---Fachinformationen.html  https://www.euronews.com/2021/11/13/vienna-to-begin-vaccinating-young-children-against-covid-19 |
| Azerbaijan | 12+ | People with uncontrolled epilepsy; patients receiving immunosuppressive therapy; pregnant women | http://sehiyye.gov.az/xeberler/3418-azrbaycan-respublikasnn-shiyy-nazirliyi-cbari-tibbi-sorta-zr-dvlt-agentliyi-v-tibbi-razi-blmlrini-daretm-birliyinin-mlumati.html  http://health.gov.az/xeberler/3477-azrbaycan-respublikas-shiyy-nazirliyinin-mlumati.html |
| Belarus | 12+ | Immunosuppressive therapy | https://cms.law/en/int/expert-guides/cms-expert-guide-to-vaccine-compensation-regimes/belarus  http://minzdrav.gov.by/ru/dlya-spetsialistov/rekomendatsii-po-vaktsinatsii-protiv-covid-19.php  https://eng.belta.by/society/view/covid-19-vaccination-rate-in-belarus-doubles-in-december-146559-2021/ |
| Belgium | 5+ | No data | <https://www.info-coronavirus.be/en/vaccination/#faq>  https://eng.belta.by/society/view/covid-19-vaccination-rate-in-belarus-doubles-in-december-146559-2021/ |
| Bosnia and Herzegovina | 12+ | No data | https://sarajevotimes.com/cerkez-recommended-vaccination-of-children-over-12-years-of-age-with-weak-immunity/ |
| Bulgaria | 5+ | People who have allergic reactions to vaccine component | https://coronavirus.bg/bg/vaccinations/faq  https://coronavirus.bg/bg/vaccinations_news/3100 |
| Croatia | 5+ | People with an immediate allergic reaction to any other vaccine or injection therapy | https://www.koronavirus.hr/o-covidu/892  https://www.koronavirus.hr |
| Cyprus | 5+ | No data | http://www.news.cn/english/2021-12/10/c_1310364684.htm |
| Czechia | 5+ | People with allergic reaction to vaccine component | <https://covid.gov.cz/en/situations/information-about-vaccine/vaccine-indication-selected-cohorts>  https://koronavirus.mzcr.cz/ockovani-proti-covid-19/ |
| Denmark | 5+ | People with a severe allergic reaction to the first shot or any ingredient of the vaccine; people with a fever | <https://www.sst.dk/da/corona/Vaccination/Saadan-bliver-du-vaccineret>  <https://www.sst.dk/-/media/Udgivelser/2021/Corona/Vaccination/Notater/Vidensbank-til-almen-praksis-og-vaccinationscentre.ashx?la=da&hash=A5DF7E41671F7370E23EE58D9124B5935FA55D3D>  https://www.sst.dk/da/corona/Vaccination/Saadan-bliver-du-vaccineret |
| Estonia | 5+ | People with a severe allergic reaction to the first shot or any ingredient of the vaccine; people with a fever; people suffering from severe frailty syndrome, are in a very bad general condition or nearing the end of their life | <https://www.kriis.ee/en/vaccination-plan-and-risk-groups>  <https://kkk.kriis.ee/en/faq/covid-19-vaccination/vaccination-plan-and-risk-groups>  https://vaktsineeri.ee/uudised/algas-5-11-aastaste-laste-vaktsineerimine-covid-19-vastu/ |
| Finland | 5+ | People with allergic reactions to the first dose or vaccine component | <https://thl.fi/fi/web/infektiotaudit-ja-rokotukset/rokotteet-a-o/koronavirusrokotteet-eli-covid-19-rokotteet-ohjeita-ammattilaisille>  <https://thl.fi/en/web/infectious-diseases-and-vaccinations/what-s-new/coronavirus-covid-19-latest-updates/vaccines-and-coronavirus/suitability-of-covid-19-vaccines-for-various-groups>  https://thl.fi/en/web/thlfi-en/-/new-decree-on-coronavirus-vaccinations-in-effect-from-23-december-coronavirus-vaccinations-may-be-offered-to-children-aged-5-11 |
| France | 5+ | People with allergic reactions to the first dose or vaccine component; people with thrombocytopenia and coagulation disorders, Capillary leak syndrome; 12-17 with developed pediatric multisystem inflammatory syndrome | <https://vaccination-info-service.fr/Les-maladies-et-leurs-vaccins/COVID-19> |
| Georgia | 12+ | Pregnant women | https://www.provax.ge/Content/files/en_Vaccination_plan.pdf  https://www.provax.ge/en/  https://vaccines.ncdc.ge/vaccinationprocess/#who |
| Germany | 12+ | People with allergies to components of the COVID-19 vaccines; people with a fever | <https://www.bundesgesundheitsministerium.de/coronavirus/faq-covid-19-impfung.html#c21988>  https://www.rki.de/SharedDocs/FAQ/COVID-Impfen/gesamt.html |
| Greece | 5+ | People with a severe allergic reaction to the first shot or any ingredient of the vaccine | People with a severe allergic reaction to the first shot or any ingredient of the vaccine  https://www.moh.gov.gr/articles/health/dieythynsh-dhmosias-ygieinhs/emboliasmoi/systaseis-emboliasmoy-kata-thn-periodo-ths-pandhmias-covid19 |
| Hungary | 5+ | No data | https://koronavirus.gov.hu/gyik  https://vakcinainfo.gov.hu/az-oltas-menete |
| Iceland | 5+ | People with a history of severe allergies | https://www.lyfjastofnun.is/covid-19/comirnaty-biontech-pfizer-2/  https://www.landlaeknir.is/um-embaettid/greinar/grein/item44019/vaccination-against-covid-19  https://www.icelandreview.com/society/vaccinations-recommended-for-chilren-aged-5-11/ |
| Ireland | 5+ | No data | https://www.gov.ie/en/press-release/0393d-minister-for-health-announces-acceleration-of-irelands-covid-19-vaccination-programme/ |
| Israel | 5+ | People with serious allergic reactions; people with a fever | https://govextra.gov.il/ministry-of-health/covid19-vaccine/en-covid19-vaccination-information/  https://corona.health.gov.il/en/vaccine-for-covid/under-12-faq/ |
| Italy | 5+ | People with allergic reactions to the first dose | https://www.aifa.gov.it/en/domande-e-risposte-su-vaccini-covid-19  https://www.salute.gov.it/portale/nuovocoronavirus/dettaglioContenutiNuovoCoronavirus.jsp?lingua=italiano&id=5452&area=nuovoCoronavirus&menu=vuoto |
| Kazakhstan | 12+ | No data | https://egov.kz/cms/en/articles/health_care/Vakcinaciya-protiv-koronavirusnoy-infekcii-  https://primeminister.kz/en/news/press/deti-ot-12-let-i-starshe-smogut-poluchit-vakcinu-pfizer-tolko-na-dobrovolnoy-osnove-i-s-soglasiya-roditeley-a-coy-2782049 |
| Kyrgyzstan | 18+ | People with allergic reaction to vaccine component; people with a fever; people with an acute attack of chronic disease; people with epilepsy; pregnant or breastfeeding women | <http://med.kg/ru/novosti/5345-kak-zashchitit-lyudej-s-osobymi-potrebnostyami-ot-sovid-19-delat-li-vaktsinatsiyu.html>  <http://med.kg/ru/vaktsinatsiya/5037-vaktsinatsiya-lits-s-nevrologicheskimi-zabolevaniyami.html> |
| Latvia | 5+ | People with a severe allergic reaction in the past after receiving other vaccines or medications | <https://www.spkc.gov.lv/lv/manavakcinalv>  https://www.spkc.gov.lv/lv/visparigi-jautajumi-par-vakcinam  https://www.spkc.gov.lv/lv/jautajumi-par-pfizer-biontech-razoto-vakcinu-comirnaty |
| Lithuania | 5+ | People with a fever | https://koronastop.lrv.lt/lt/duk/vakcinacija-nuo-covid-19/vakcinacija-nuo-12-metu-amziaus  https://experience.arcgis.com/experience/cab84dcfe0464c2a8050a78f817924ca/page/page_3/ |
| Luxembourg | 5+ | No data | https://covid19.public.lu/fr/vaccination/infovaxx.html  https://msan.gouvernement.lu/en/actualites.gouvernement%2Ben%2Bactualites%2Btoutes_actualites%2Bcommuniques%2B2021%2B12-decembre%2B23-vaccination-enfants.html |
| Malta | 5+ | People with a history of serious allergy (anaphylaxis) following a vaccine or injectable medication | https://deputyprimeminister.gov.mt/en/health-promotion/covid-19/Pages/frequently-asked-questions.aspx  https://deputyprimeminister.gov.mt/en/health-promotion/covid-19/Pages/travel.aspx |
| Moldova | 12+ | No data | http://vaccinare.gov.md/permitted-vaccines  https://vaccinare.gov.md/news/15-implementarea-procesului-de-vaccinare-impotriva-covid-19-in-republica-moldova |
| Monaco | 12+; 5+ at high risk | No data | https://covid19.mc/en/fight-against-coronavirus/vaccination/qui-peut-se-faire-vacciner-contre-la-covid19/  https://covid19.mc/lutter-contre-la-covid-19/vaccination/  https://covid19.mc/en/fight-against-coronavirus/vaccination/qui-peut-se-faire-vacciner-contre-la-covid19/ |
| Montenegro | 5+ | No data | https://www.covidodgovor.me/me/cesta-pitanja |
| Netherlands | 5+ | No data | https://www.government.nl/topics/coronavirus-covid-19/dutch-vaccination-programme |
| North Macedonia | 12+ | No data | http://zdravstvo.gov.mk/sq/jane-aplikuar-1-435-392-doza-te-vaksines-ndersa-jane-rivaksinuar-667-739-qytetare/ |
| Norway | 5+ | People with allergic reactions to the first dose; people with a fever | https://www.fhi.no/sv/vaksine/koronavaksinasjonsprogrammet/koronavaksine/#vaksinering-av-personer-som-har-gjennomgaatt-covid19- |
| Poland | 5+ | People with allergic reactions to the first dose or vaccine component; people with immunodeficiency; pregnant or breastfeeding women | <https://www.gov.pl/web/szczepimysie/narodowy-program-szczepien-przeciw-covid-19>  <https://www.gov.pl/web/szczepimysie/jak-sie--zaszczepic>  <https://www.gov.pl/web/szczepimysie/pytania-i-odpowiedzi>  https://www.gov.pl/web/szczepimysie/materialy-informacyjne-dla-szpitali-i-pacjentow-dotyczace-szczepien-przeciw-covid-19 |
| Portugal | 5+ | No data | https://covid19.min-saude.pt/perguntas-frequentes/  https://covid19.min-saude.pt/pedido-de-agendamento/ |
| Romania | 5+ | No data | https://vaccinare-covid.gov.ro/vaccinuri-autorizate/  https://www.euractiv.com/section/politics/short_news/romania-vaccinates-more-than-5000-children-in-a-week/ |
| Russia | 18+ | People with allergic reaction to vaccine component; people with a fever; people with an acute attack of chronic diseases; pregnant or breastfeeding women | https://static-0.minzdrav.gov.ru/system/attachments/attaches/000/054/706/original/%D0%9E%D1%82%D0%B2%D0%B5%D1%82%D1%8B_%D0%BF%D0%BE_%D0%B2%D0%B0%D0%BA%D1%86%D0%B8%D0%BD%D0%B0%D1%86%D0%B8%D0%B8_COVID_19-19.02.2021-Red.pdf |
| San Marino | 5+ | People with allergic reaction;  People taking anticoagulant therapy | https://vaccinocovid.iss.sm/faq  https://vaccinocovid.iss.sm/vaccinazioni-anticovid-fascia%20et%C3%A0-5-11%20anni |
| Serbia | 12+ | People with fever | https://www.france24.com/en/video/20210326-go-to-serbia-for-a-free-covid-vaccine-country-offers-jabs-to-foreigners  https://vakcinacija.gov.rs/vakcine-protiv-covid-19-u-srbiji/  https://borgenproject.org/covid-19-vaccinations-in-serbia/  https://vakcinacija.gov.rs/vakcine-protiv-covid-19-u-srbiji/ |
| Slovakia | 12+ | No data | https://www.slovenskoproticovidu.sk/sk/vsetko-o-ockovani/vakciny/viac-o-vakcine-comirnaty-od-vyrobcov-pfizer-biontech |
| Slovenia | 5+ | People with severe allergy to the ingredients of the vaccine; people with a fever; people with thrombosis syndrome with thrombocytopenia after the first vaccination with Vaxzevria | https://www.nijz.si/sites/www.nijz.si/files/uploaded/priporocila_za_cepljenje_proti_covid_uskl_psc_apr_2021.pdf  https://www.cepimose.si/cepljenje-proti-covidu-19/pogosta-vprasanja-in-odgovori/  https://sloveniatimes.com/covid-19-vaccination-of-children-aged-5-11-starting/ |
| Spain | 5+ | People with a history of having had severe allergic reactions (e.g., anaphylaxis) to any component of the vaccine | https://www.vacunacovid.gob.es/preguntas-y-respuestas/deben-vacunarse-los-ninos-y-las-ninas-y-la-poblacion-adolescente  https://www.vacunacovid.gob.es/preguntas-y-respuestas |
| Sweden | 12+ | No data | https://www.folkhalsomyndigheten.se/smittskydd-beredskap/utbrott/aktuella-utbrott/covid-19/vaccination-mot-covid-19/fragor-och-svar-om-vaccination-mot-covid-19/  https://www.1177.se/en/other-languages/other-languages/covid-19/vaccin-engelska/#section-133829 |
| Switzerland | 5+ | No data | https://www.swissmedicinfo.ch/ShowText.aspx?textType=FI&lang=DE&authNr=68225  https://www.ge.ch/en/getting-vaccinated-against-covid-19/vaccination-scheme |
| Tajikistan | 18+ | No data | https://apa.az/en/xeber/cis-countries-news/tajikistan-declares-mandatory-covid-19-vaccination-353147  https://eurasianet.org/dashboard-vaccinating-eurasia-june |
| Turkey | 12+ | People with allergic reactions to the first dose or vaccine component | <https://covid19asi.saglik.gov.tr/EN-78316/frequently-asked-questions.html>  https://www.hurriyetdailynews.com/erdogan-urges-parents-teachers-to-get-covid-19-vaccine-167672  https://www.dailysabah.com/turkey/turkey-lowers-covid-19-vaccination-age-to-15-starts-fourth-doses/news |
| Turkmenistan | 18+ | No data | https://tdh.gov.tm/en/post/25815/objectives-of-increasing-national-health-system%E2%80%99s-potential-discussed  https://www.barrons.com/news/uzbekistan-certifies-russia-s-sputnik-vaccine-for-mass-use-01613559912  https://www.garda.com/crisis24/news-alerts/508931/turkmenistan-authorities-tighten-covid-19-countermeasures-in-ashgabat-as-of-aug-2-update-16  https://tdh.gov.tm/en/post/25911/turkmenistan-registers-vaccines-prevention-infectious-diseases |
| Ukraine | 12+ | People with any contraindications or allergies to any vaccine; people with an acute illness and body temperature above 38.5ºC | https://vaccination.covid19.gov.ua/faq  https://www.kyivpost.com/ukraine-politics/ukraine-allows-children-over-12-to-be-vaccinated-with-pfizer.html |
| United Kingdom | 12+; some children aged 5 to 11 can get a 1st and 2nd dose of the COVID-19 vaccine if either: they have a condition that means they're at high risk of getting seriously ill from COVID-19, or they live with someone who has a weakened immune system | No data | https://www.gov.uk/government/publications/regulatory-approval-of-pfizer-biontech-vaccine-for-covid-19/information-for-uk-recipients-on-pfizerbiontech-covid-19-vaccine  https://www.nhs.uk/conditions/coronavirus-covid-19/coronavirus-vaccination/who-can-get-the-vaccine/ |
| Uzbekistan | 12+ | No data | https://xs.uz/uz/post/covid-19ga-qarshi-12-yoshdan-katta-bolalar-ham-ikhtiyorij-emlanadi |
| **South-East Asia** | | | |
| Bangladesh | 12+ | No data | https://www.facebook.com/opmbs/posts/from-the-statement-the-astrazeneca-vaccine-will-be-administered-to-eligible-baha/762781414443838/ |
| Bhutan | 12+ | People with a severe allergic reaction to the first shot or any ingredient of the vaccine; pregnant or breastfeeding women | <https://drive.google.com/file/d/1UMlrlI-HvD5x1q99NHi50LoMP-_qjaIA/view>  <https://www.gov.bt/covid19/03-03-21-information-series-on-covid-19-vaccines-moh/> |
| India | 15+ | People with a severe allergic reaction to the first shot or any ingredient of the vaccine; pregnant or breastfeeding women; people with severe illness | https://www.mohfw.gov.in/vaccinationbooklet/#page/1  <https://www.mohfw.gov.in/covid_vaccination/vaccination/faqs.html#about-the-vaccine>  https://www.cowin.gov.in/ |
| Indonesia | 6+ | No data | https://www.kemkes.go.id/article/view/21121400001/pemerintah-gelar-kick-off-vaksinasi-covid-19-anak-usia-6-11-tahun-serentak-di-3-provinsi.html |
| Maldives | 12+ | People with a fever | [http://health.gov.mv/Uploads/Downloads//Informations/Informations(362).pdf](http://health.gov.mv/Uploads/Downloads/Informations/Informations(362).pdf)  https://covid19.health.gov.mv/vaccination/?c=0  https://raajje.mv/104861 |
| Myanmar | 12+ | No data | https://www.mohs.gov.mm/page/17483  http://www.xinhuanet.com/english/asiapacific/2021-10/10/c_1310236389.htm |
| Nepal | 5+ | No data | https://reliefweb.int/sites/reliefweb.int/files/resources/Focused%20COVID-19_Media%20Monitoring_31%20January%202022.pdf |
| North Korea | No data | No data |  |
| Sri Lanka | 12+ | No data | http://www.epid.gov.lk/web/index.php?option=com_content&view=article&id=131:topics-recent&Itemid=487&lang=en  http://www.epid.gov.lk/web/index.php?option=com_content&view=article&id=131:topics-recent&Itemid=487&lang=en |
| Thailand | 5+ | No data | https://thainews.prd.go.th/en/news/detail/TCATG210727104313781  https://thethaiger.com/news/national/more-kids-aged-5-11-getting-covid-19 |
| Timor-Leste | 12+ | No data | https://www.facebook.com/profile/100064941300042/search/?q=Pfizer |
| **Western Pacific** | | | |
| Australia | 5+ | No data | https://www.health.gov.au/initiatives-and-programs/covid-19-vaccines/getting-vaccinated-for-covid-19  https://www.health.gov.au/committees-and-groups/australian-technical-advisory-group-on-immunisation-atagi#statements |
| Brunei | 12+ | No data | <http://www.moh.gov.bn/Shared%20Documents/COVID-19%20Vaccine/FAQs%20General%20Covid%2019%20Vaccine_04032021.pdf>  https://www.thestar.com.my/aseanplus/aseanplus-news/2021/10/20/brunei-to-vaccinate-children-aged-12-17  <http://www.moh.gov.bn/Shared%20Documents/COVID-19%20Vaccine/Brunei%20Darussalam%20Vaccination%20Strategy%20V2.pdf>  https://www.thestar.com.my/aseanplus/aseanplus-news/2021/10/20/brunei-to-vaccinate-children-aged-12-17 |
| Cambodia | 5+ | No data | https://www.reuters.com/business/healthcare-pharmaceuticals/cambodia-starts-coronavirus-vaccinations-young-children-2021-09-17/ |
| China | 3+ | Those who are allergic to the ingredients of the vaccine or who have history of allergy to the same type of vaccine; history of serious allergy to vaccines (such as acute allergic reactions, angioedema, breathing difficulty); people with uncontrolled epilepsy and other serious neurological diseases; patients with fever, or acute diseases, or during acute attacks of chronic diseases, or patients with uncontrolled severe chronic diseases; women during pregnancy | <http://www.nhc.gov.cn/wjw/hygq/202104/8e62004e41d648d5a084b3fb7bf098ea.shtml>  http://english.nmpa.gov.cn/2021-04/01/c_608350.htm  https://www.twoeggz.com/info/903497.html |
| Cook Islands | 5+ | No data | https://covid19.gov.ck/sites/default/files/2022-02/Media%20Release%20-%20Vaccination%20Roll-Out%20Update%206%20February%20Final.pdf |
| Fiji | 12+ | People with allergic reactions to the first dose | <https://www.health.gov.fj/covid-19-vaccination-campaign/>  https://www.health.gov.fj/covid-vaccine/vaccine-faqs/  https://www.fbcnews.com.fj/news/covid-19/pfizer-vaccine-to-rollout-from-15th/ |
| Japan | 12+ | People with allergic reactions to vaccine component; people with a fever | <https://www.mhlw.go.jp/stf/covid-19/vaccine.html>  <https://www.cov19-vaccine.mhlw.go.jp/qa/receive/>  https://www.mhlw.go.jp/content/000759294.pdf |
| Kiribati | 18+ | No data | https://www.mhms.gov.ki/single.php?id=26 |
| Laos | 12+ | No data | https://www.thestar.com.my/aseanplus/aseanplus-news/2021/09/18/laos-health-ministry-advises-covid-19-vaccine-jabs-for-pregnant-women-and-older-students  https://drive.google.com/file/d/1n1U1b3fOm2mlju2qDtJ0MhPvjVXK4WUu/view |
| Malaysia | 5+ | People with allergic reaction to vaccine component; people with immunodeficiency | https://www.npra.gov.my/easyarticles/images/users/1047/Frequently-Asked-Questions-FAQ-about-Comirnaty-Covid-19-Vaccine.pdf  https://www.reuters.com/world/asia-pacific/malaysia-approves-pfizers-covid-19-vaccine-children-aged-5-11-2022-01-06/ |
| Marshall Islands | 18+ | No data | <https://www.rnz.co.nz/international/pacific-news/436910/marshalls-leads-pacific-s-covid-19-vax-charg> |
| Micronesia | 5+ | No data | https://hsa.gov.fm/fsm-covid-19-vaccination/  https://www.facebook.com/piofsm/posts/4582650958483833 |
| Mongolia | 12+ | No data | https://moh.gov.mn/news/ |
| Nauru | 12+ | No data | http://naurugov.nr/media/146514/nauru_bulletin__03_9jul2021__228_.pdf |
| New Zealand | 5+ | People with a history of anaphylaxis to any component or previous dose of mRNA-CV | https://www.health.govt.nz/our-work/immunisation-handbook-2020/5-coronavirus-disease-covid-19  https://www.health.govt.nz/our-work/diseases-and-conditions/covid-19-novel-coronavirus/covid-19-vaccines/covid-19-vaccine-health-advice/covid-19-vaccine-severely-immunocompromised-people |
| Niue | 12+ | No data | https://covid19.govt.nz/iwi-and-communities/translations/niuean/ |
| Palau | 5+ | No data | https://covid19.govt.nz/iwi-and-communities/translations/niuean/the-covid-19-vaccine/vaccine-basics/  http://www.palauhealth.org/2019nCoV/PR-old/MHHS%20PSA%20VACC%20SCHEDULE%2002042022.pdf |
| Papua New Guinea | 18+ | No data | https://postcourier.com.pg/chinese-vaccine-to-be-gazetted/  https://www.pna.gov.ph/articles/1167527 |
| Philippines | 5+ | People with allergic reactions to vaccine component or the first dose | https://doh.gov.ph/vaccines  https://doh.gov.ph/vaccines/when-will-the-COVID-19-available-to-me |
| Samoa | 5+ | People with allergic reactions to vaccine component; people with a fever; people taking anticoagulant therapy; people with immunodeficiency; pregnant women; 85+ (optional) | https://vaccinocovid.iss.sm/faq  https://www.samoaobserver.ws/category/samoa/90307 |
| Singapore | 5+ | No data | <https://www.gov.sg/features/covid-19-vaccination>  https://www.moh.gov.sg/covid-19/vaccination |
| Solomon Islands | 12+ | No data | https://solomons.gov.sb/roll-out-of-2nd-dose-of-astrazeneca-vaccine-now-underway-in-honiara/  https://www.facebook.com/officialmhmssi/posts/229197692717629 |
| South Korea | 12+ | People with allergic reactions to the first dose or vaccine component; pregnant women; people with acute symptoms such as fever (37.5℃ or higher) | http://english.seoul.go.kr/covid/covid-19-vaccination-guideline/  https://www.reuters.com/world/asia-pacific/skorea-vaccinate-12-17-year-olds-give-boosters-elderly-2021-09-27/ |
| Tonga | 12+ | No data | https://matangitonga.to/2021/08/24/tonga-fully-vaccinates-43-eligible-people-over-18?fbclid=IwAR3KFE6s_Brg8uj0JWH-YhmejAg1fdrlv-2nh95NOLu_uL510fdPSfOCeT4  https://www.facebook.com/mohtonga/posts/339635561322313 |
| Tuvalu | 18+ | No data | https://www.facebook.com/AusHCfnfu/posts/750425468961962 |
| Vanuatu | 18+ | No data | https://covid19.gov.vu/index.php/vaccination/information |
| Vietnam | 5+ | No data | https://en.vietnamplus.vn/ministry-grants-conditional-approval-of-pfizer-biontech-vaccine/202977.vnp  https://vietnamnews.vn/society/1142625/govt-agrees-to-buy-219m-pfizer-vaccine-shots-for-children-aged-5-11-years.html |

Abbreviation: 18+, people aged 18 years and above; HCW, healthcare workers

### Table S3. Policies on additional or booster dose of COVID-19 vaccine

| **Country** | **Policy on additional dose** | **Policy on booster dose** | **Data source** |
| --- | --- | --- | --- |
| **Africa** | | | |
| Angola | No data | **Indication**: 18+ | https://newsaf.cgtn.com/news/2021-12-16/Angola-to-expand-COVID-19-vaccination-to-reach-12-year-olds-161ntZI5Epq/index.html |
| Botswana | No data | **Indication**: 18+  **Vaccines in use**: Comirnaty, Vaxzevria, Covishield, Janssen COVID-19 Vaccine, Spikevax, CoronaVac  **Interval**: 180 d | http://www.xinhuanet.com/english/20220116/bf56abfdf49548909bbc4102dcea6a18/c.html |
| Cabo Verde | No data | **Indication**: 40+, people with underlying conditions | https://www.rfi.fr/pt/cabo-verde/20211221-cabo-verde-inicia-vacina%C3%A7%C3%A3o-de-refor%C3%A7o-para-faixa-et%C3%A1ria-de-40-e-mais-anos |
| Eswatini | No data | **Indication**: 18+  **Vaccines in use**: Comirnaty, Janssen COVID-19 Vaccine  **Interval**: 180 d | https://eswatinihealth.org/boosters/ |
| Gambia | No policy | No policy |  |
| Ghana | No data | **Indication**: 60+, healthcare workers, people with underlying conditions, frontline security personnel, members of the Executive, Judiciary and Legislature  **Interval**: 90-180 d | https://www.pulse.com.gh/news/local/ghana-starts-giving-covid-19-vaccine-booster-shots/532z0k4 |
| Guinea-Bissau | No data | **Indication**: 18+  **Interval**: 180 d | https://www.jamaicaobserver.com/latestnews/Guyana_to_give_booster_shots_to_persons_over_18_years_old |
| Guyana | No data | **Indication**: 18+  **Vaccines in use and interval**: For persons who took the Johnson and Johnson vaccine, a booster shot of the Sinopharm vaccine is recommended, 52 days after the dose. Those who took the Sinopharm vaccine, can take the same vaccine as their booster shot three to six months after the second jab. It is advised that persons who have been immunized using the AstraZeneca vaccine, can receive an MRNA vaccine which should be taken six months after the second dose. A shot of the Johnson and Johnson vaccine can be used as a booster shot for the Sputnik V vaccine  **Interval**: 180 d | http://radiojamaicanewsonline.com/local/guyana-offering-covid-booster-shot-to-all-adults |
| Kenya | No data | **Indication**: 18+  **Vaccines in use**: Comirnaty, Spikevax, Janssen COVID-19 Vaccine  **Interval**: 180 d | https://newsaf.cgtn.com/news/2021-12-26/Kenya-set-to-start-administering-COVID-19-vaccine-booster-jabs--16i8YeH5KFi/index.html |
| Malawi | No data | No policy |  |
| Mauritania | No data | **Indication**: 18+ | https://www.facebook.com/UNICEFMauritanie/posts/3009669819275108 |
| Mauritius | No data | **Indication:** 40+  **Vaccines in use**: Comirnaty, Janssen COVID-19 Vaccine  **Interval**: 120 d | https://www.zawya.com/mena/en/press-releases/story/Mauritius__COVID19_Pfizer_Booster_Dose_to_be_administered_to_those_aged_over_40-AFPR0601202247990/ |
| Namibia | No data | **Indication**: 18+ | https://allafrica.com/stories/202112010479.html |
| Niger | No data | No policy |  |
| Nigeria | No data | **Indication:** 18+ |  |
| Rwanda | No data | **Indication**: 12+  **Interval**: 90 d | https://vaccinare-covid.gov.ro/doza-de-rapel-booster/ |
| Sao Tome and Principe | No data | **Indication**: healthcare workers, teachers | https://e-global.pt/noticias/lusofonia/sao-tome-e-principe/o-reforco-da-vacina-contra-covid-19-arranca-em-sao-tome-e-principe/ |
| Senegal | No data | **Indication**: 18+  **Interval**: 180 d | https://www.reuters.com/world/africa/senegal-authorizes-covid-19-booster-shots-vaccines-children-2022-01-13/ |
| Seychelles | No data | **Indication**: 18+ | https://www.facebook.com/watch/?v=1008352873229494 |
| Sierra Leone | No data | **Indication**: 60+, healthcare workers, people with immunosuppression  **Interval**: 180 d | https://dhse.gov.sl/corona-virus-covid-19-information/ |
| South Africa | **Indication**: people with immunosuppression | **Indication:** 18+  **Vaccines in use**: Comirnaty, Janssen COVID-19 Vaccine  **Interval**: 180 d | https://www.sanews.gov.za/south-africa/covid-19-booster-shot-immunocompromised-south-africans |
| Togo | No data | **Indication**: 18+  **Interval**: 60 d | https://www.africa-press.net/togo/toutes-les-actualites/covid-19-vaccination-togo-begins-the-booster-dose |
| Uganda | No data | **Indication:** 50+, healthcare workers, teachers, religious leaders, cultural leaders, security personnel, media, drivers and conductors of passenger service vehicles, boda boda riders, bar and nightclub workers, market workers and vendors | http://www.china.org.cn/world/Off_the_Wire/2022-01/17/content_77995294.htm |
| United Republic of Tanzania | No data | No policy |  |
| Zambia | No data | **Indication**: 18+  **Vaccines in use**: mRNA vaccines or the same type in primary immunization  **Interval**: 180 d | https://www.aa.com.tr/en/africa/zambia-introduces-covid-19-booster-shots/2458815 |
| Zimbabwe | No data | **Indication**: 18+  **Vaccines in use**: CoronaVac | https://www.sundaynews.co.zw/covid-19-schools-to-remain-open-booster-shots-start/ |
| **Americas** | | | |
| Antigua and Barbuda | No data | **Indication**: 12+  **Vaccines in use**: Comirnaty or the same type in primary immunization  **Interval**: 60-180 d | https://www.facebook.com/photo/?fbid=591469248797383&set=a.582034583074183 |
| Argentina | **Indication**: people with immunosuppression, 50+ receiving Sinopharm  **Interval**: 28 d | **Indication**: 18+  **Vaccines in use**: Comirnaty, Spikevax, Vaxzevria, Sputnik V, Convidecia  **Interval**: 120 d | https://www.argentina.gob.ar/coronavirus/vacuna/preguntas-frecuentes#20  https://bancos.salud.gob.ar/recurso/memorandum-recomendacion-sobre-el-intervalo-de-dosis-de-refuerzo-de-vacunacion-contra-covid |
| Bahamas | **Indication**: people with immunosuppression  **Interval**: 30-90 d | **Indication**: 12+  **Vaccines in use**: Comirnaty, Vaxzevria  **Interval**: 60-150 d | https://vax.gov.bs |
| Barbados | No data | **Indication**: 18+  **Vaccines in use**: All types  **Interval**: 180 d | https://gisbarbados.gov.bb/blog/booster-shot-now-for-all-eligible-persons/ |
| Belize | No data | **Indication**: People living with comorbidities; 60+  **Interval**: 60-180 d | https://amandala.com.bz/news/booster-shots-approved-in-belize/ |
| Bolivia | No data | **Indication**: 18+  **Interval**: 120-300 d | https://www.plenglish.com/news/2021/11/29/covid-19-booster-vaccines-are-being-applied-in-bolivia/ |
| Brazil | **Indication**: People with immunosuppression  **Vaccines in use:** Covishield, Vaxzevria, Comirnaty, Janssen COVID-19 Vaccine  **Interval**: 56 d | **Indication**: 18+  **Vaccines in use:** Covishield, Vaxzevria, Comirnaty, Janssen COVID-19 Vaccine  **Interval**: 120 d | https://www.gov.br/saude/pt-br/coronavirus/vacinas/NTDoseReforo.pdf  https://www.reuters.com/world/americas/brazil-offer-covid-19-booster-shot-everyone-older-than-18-2021-11-16/ |
| Canada | **Indication**: People with moderate to severe immunocompromision  **Interval**: 28 d | **Indication**: 18+  **Vaccines in use**: Comirnaty, Spikevax  **Interval**: 180 d | <https://www.canada.ca/en/public-health/services/immunization/national-advisory-committee-on-immunization-naci/statement-september-10-2021-additional-dose-covid-19-vaccine-immunocompromised-following-1-2-dose-series.html>  https://www.canada.ca/content/dam/phac-aspc/documents/services/immunization/national-advisory-committee-on-immunization-naci/guidance-booster-covid-19-vaccine-doses/guidance-booster-covid-19-vaccine-doses.pdf |
| Chile | No data | **Indication:** 12+  **Indication:** People receiving Sinovac  **Interval**: 90 d | http://outbreaknewstoday.com/chile-begins-second-covid-19-booster-for-immunocompromised-soon-55-and-older-37116/ |
| Colombia | **Indication**: People with immunosuppression  **Vaccines in use**: Comirnaty, Spikevax  **Interval**: 28 d | **Indication:** 12+  **Vaccines in use**: Comirnaty, Spikevax, Janssen COVID-19 Vaccine  **Interval**: 120 d | https://www.como.gov/covidvaccine/#elementor-toc__heading-anchor-5  https://www.reuters.com/business/healthcare-pharmaceuticals/colombia-reduces-booster-vaccine-waiting-time-four-months-2022-01-12/ |
| Costa Rica | No data | **Indication**: 58+, essential workers, teachers  **Interval**: 150 d | https://www.telesurenglish.net/news/5-Months-to-Get-3rd-COVID-Vaccine-Dose-in-Costa-Rica-20220204-0017.html |
| Cuba | No data | **Indication**: 2+  **Vaccines in use**: Soberana-02  **Interval**: 90 d  **Interval for COVID-19 survivals**: 90 d | https://www.granma.cu/cuba/2022-01-11/convalecientes-de-la-covid-19-podran-recibir-dosis-de-refuerzo-de-soberana-plus-video |
| Dominica | No data | **Indication**: 65+, 18+ living in nursing homes, 18+ with underlying conditions, essential workers  **Interval**: 180 d | https://dominicanewsonline.com/news/homepage/news/close-to-2000-booster-vaccines-administered-as-of-january-11-2022-cmo-johnson/ |
| Dominican Republic | No data | **Indication**: 18+ | https://crisis24.garda.com/alerts/2022/01/dominican-republic-authorities-to-require-a-third-dose-of-a-covid-19-vaccine-for-individuals-to-enter-multiple-businesses-effective-jan-31-update-47 |
| Ecuador | **Indication**: People with weak immune systems | **Indication**: 18+  **Interval**: 180 d | https://www.reuters.com/world/americas/ecuador-give-immune-weakened-people-third-covid-19-vaccine-2021-08-17/  https://www.coronavirusecuador.com/2022/02/se-adelanta-refuerzo-contra-covid-19-para-poblacion-desde-45-anos/ |
| El Salvador | No data | **Indication**: 18+  **Interval**: 120 d | https://www.presidencia.gob.sv/el-salvador-supero-el-de-aplicaciones-de-la-tercera-dosis-de-la-vacuna-anticovid-19/ |
| Grenada | No data | **Indication**: 18+ | https://caribbean.loopnews.com/content/grenada-begins-its-pfizer-vaccine-roll-out-soon |
| Guatemala | No data | **Indication**: 18+  **Interval**: 120 d | https://24newsrecorder.com/world/78123 |
| Honduras | **Indication**: People with immunosuppression | **Indication**: 18+, pregnant women aged 12-17  **Interval**: 90 d | http://www.salud.gob.hn/site/index.php/component/k2/item/2457-la-secretaria-de-salud-amplio-la-vacunacion-contra-el-covid-19-a-menores-de-10-anos |
| Jamaica | No data | **Indication**: 18+  **Vaccines in use**: Comirnaty, Janssen COVID-19 Vaccine (only for the same type in the primary vaccination)  **Interval**: 180 d | https://www.moh.gov.jm/jamaica-opens-booster-programme-to-all/ |
| Mexico | No data | **Indication**: 60+ | https://www.reuters.com/business/healthcare-pharmaceuticals/mexico-reports-110-more-covid-19-deaths-752-new-cases-2021-12-07/ |
| Nicaragua | No data | **Indication**: 18+ | https://www.laprensani.com/2022/02/12/nacionales/2949499-es-nicaragua-el-segundo-pais-mas-avanzado-del-istmo-en-cuanto-a-la-vacunacion-contra-la-covid-19 |
| Panama | **Indication**: People with immunosuppression  **Interval**: 28 d | **Indication**: 16+  **Interval**: 90 d | https://www.reuters.com/world/americas/panama-give-immunocompromised-people-third-covid-19-vaccine-shot-2021-09-22/  https://www.minsa.gob.pa/noticia/mas-de-31800-dosis-de-refuerzo-se-han-administrado-en-los-santos |
| Paraguay | No data | **Indication, vaccines in use & interval**: Adolescents aged 12 to 17 who have completed the vaccination schedule, with a minimum interval of 4 months, may receive the booster dose with the Pfizer platform.  Pregnant women from 12 weeks of gestation, with a minimum interval of 4 months from the application of the second dose, may receive the booster dose with the Pfizer platform.  People aged 18 and over who have completed their scheme in Paraguay or abroad and health personnel:  Those who applied Coronavac, Covaxin, Sinopharm, HayatVax, Pfizer, AstraZeneca, Sputnik V or Moderna receive Pfizer or AstraZeneca. With a minimum interval of 4 months, from the application of the second dose.  Those who applied the Janssen (J&J) receive Pfizer or Moderna. With an interval of at least 3 months from its application. | https://www.mspbs.gov.py/portal/24783/dosis-disponibles-para-acceder-a-vacunacion-contra-covid-19.html |
| Peru | No data | **Indication**: 18+  **Interval**: 90 d | https://www.youtube.com/watch?v=NiqPRcPo_1A |
| Saint Kitts and Nevis |  | **Indication**: 65+, people with underlying conditions  **Interval**: 180 d | https://caribbean.loopnews.com/content/nevis-covid-19-booster-shots-only-available-most-vulnerable |
| Saint Lucia | No data | **Indication**: 18+  **Vaccines in use**: Comirnaty  **Interval**: 180 d | http://health.govt.lc/news/covid-19-booster-doses-available-to-the-public |
| Saint Vincent and the Grenadines |  | **Indication**: 18+  **Vaccines in use**: Sputnik Light, Comirnaty, Vaxzevria  **Interval**: 150 d | https://www.facebook.com/SVGHEALTH/posts/403197071603040 |
| Suriname | No data | **Indication**: 18+  **Interval**: 90 d | http://www.dwtonline.com/mobiel/?node=524060 |
| Trinidad and Tobago | No data | **Indication**: 18+  **Vaccines in use**: All types  **Interval**: 180 d | https://health.gov.tt/covid-19-vaccine-booster-programme |
| United States | **Indication**: People with immunosuppression  **Interval**: 28 d | **Indication**: 12+  **Vaccines in use**: All types  **Interval**: 60-90 d | https://www.cdc.gov/coronavirus/2019-ncov/vaccines/booster-shot.html  https://www.fda.gov/news-events/press-announcements/fda-issues-emergency-use-authorization-third-covid-19-vaccine |
| Uruguay | **Indication**: People with immunosuppression  **Interval**: 30 d | **Indication**: 18+  **Vaccines in use**: Comirnaty  **Interval**: 120 d  **Interval for COVID-19 survivals**: 120 d | https://www.gub.uy/ministerio-salud-publica/comunicacion/noticias/administracion-dosis-refuerzo-vacuna-contra-covid-19-inmunodeprimidos  https://www.gub.uy/ministerio-salud-publica/comunicacion/publicaciones/preguntas-frecuentes-vacunacion-covid-19/preguntas-frecuentes-13 |
| Venezuela | No data | **Indication**: HCW; the elderly | https://www.telesurenglish.net/news/Venezuela-Begins-Booster-Vaccination-20220103-0001.html |
| **Eastern Mediterranean** | | | |
| Afghanistan | No data | No data |  |
| Bahrain | No data | **Indication:** 18+  **Vaccines in use**: Comirnaty or the same type in primary immunization  **Interval**: 90-180 d  **Interval for COVID-19 survivals**: 180 d | https://healthalert.gov.bh/en/category/vaccine  https://healthalert.gov.bh/en/article/booster-shot-protocols-have-been-updated-2021-12-01 |
| Egypt | No data | **Indication**: 12+  **Vaccines in use**: All types  **Interval**: 180 d | http://www.news.cn/english/2021-12/16/c_1310377133.htm |
| Iran | No data | **Indication**: 18+ | https://financialtribune.com/articles/national/112384/over-19m-iranians-received-covid-booster-shot |
| Iraq | No data | **Indication**: all people at high risk of severe disease, others who are regularly exposed to the virus, 65+  **Interval**: 180 d | https://shafaq.com/ku/Iraq-News/Iraq-approves-a-booster-COVID-19-dose-for-Healthcare-workers-and-patients-at-risk |
| Jordan | No data | **Indication**: 18+  **Interval**: 90 d | https://www.thenationalnews.com/coronavirus/2021/11/14/jordan-offers-fifth-of-adults-covid-booster-jab/ |
| Kuwait | No data | **Indication**: 16+  **Interval**: 180 d | https://cov19vaccine.moh.gov.kw/SPCMS/CVD_19_Vaccine_Booster_Registration.aspx |
| Lebanon | **Indication**: People with immunosuppression; people receiving Sinopharm | **Indication**: 12+  **Interval**: 150 d | https://today.lorientlejour.com/article/1286872/ready-for-your-covid-19-booster-heres-how-you-can-get-it-and-why-health-experts-say-you-should.html |
| Libya | No data | **Indication**: 50+, people with underlying conditions, healthcare workers | https://lana.gov.ly/post.php?lang=en&id=224594 |
| Morocco | No data | **Indication**: 18+  **Interval**: 120 d | https://en.hespress.com/33206-morocco-cuts-interval-between-second-and-booster-shot-to-4-months.html |
| Oman | No data | **Indication**: 18+  **Vaccines in use**: Comirnaty, Vaxzevria or Covishield (only for the same type in the primary vaccination)  **Interval**: 90 d | https://www.bna.bh/en/Omanshortensboosterdoseintervalto3months.aspx?cms=q8FmFJgiscL2fwIzON1%2BDvGNVscqhKvyxpo2SE2Dviw%3D |
| Pakistan | **Indication**: 12+ international travelers  **Interval**: 28 d | **Indication**: 18+  **Interval**: 180 d  **Interval for COVID-19 survivals**: 28 d | https://ncoc.gov.pk/covid-vaccination-en.php |
| Qatar | No data | **Indication:** 12+  **Interval**: 180 d | https://covid19.moph.gov.qa/EN/Covid19-Vaccine/Pages/FAQ.aspx  https://www.moph.gov.qa/english/mediacenter/News/Pages/NewsDetails.aspx?ItemId=459 |
| Saudi Arabia | No data | **Indication:** 18+  **Interval**: 90 d  **Interval for COVID-19 survivals**: 10 d | https://gulfnews.com/world/gulf/saudi/saudi-arabia-booster-shot-available-3-months-after-2nd-dose-1.84541126 |
| Tunisia | No data | **Indication**: 50+ with underlying conditions  **Interval**: 150 d | https://allafrica.com/stories/202109280209.html |
| United Arab Emirates | No data | **Indication:** 16+  **Vaccines in use**: Comirnaty or the same type in primary immunization  **Interval**: 180 d | https://www.wam.ae/en/details/1395302977389  https://u.ae/en/information-and-services/justice-safety-and-the-law/handling-the-covid-19-outbreak/eligibility-for-covid19-booster |
| **Europe** | | | |
| Albania | **Indication**: 12+ with immunosuppression | **Indication**: 18+  **Interval**: 180 d | https://www.euractiv.com/section/politics/short_news/albanian-government-rolls-out-covid-19-boosters-for-over-18s/ |
| Andorra | **Indication**: people with immunosuppression; people on dialysis; people receiving a transplant | **Indication**: 18+ | https://www.govern.ad/comunicats/item/13197-s-inicia-l-administracio-de-la-tercera-dosi-voluntaria-als-residents-a-centres-sociosanitaris-de-gent-gran |
| Armenia | No data | **Indication**: 18+  **Interval**: 180 d  **Interval for COVID-19 survivals**: 180 d | https://en.armradio.am/2021/12/01/armenia-offers-covid-19-boosters-to-adults/ |
| Austria | **Indication**: Personnel in old people's, nursing and retirement homes; health care personnel; personnel in mobile care, support, nursing and 24-hour care as well as caring relatives; staff in educational institutions (childcare, school, university, etc.)  **Interval**: 270-365 d | **Indication**: 12+  **Interval**: 180 d  **Interval for COVID-19 survival**s: 180 d | https://www.thelocal.at/20210827/first-austrian-state-to-start-covid-booster-shots-from-monday/  https://www.thelocal.at/20211101/austria-to-roll-out-covid-booster-shots-from-tuesday/ |
| Azerbaijan | No data | **Indication**: HCW; 60+; people with immunosuppression  **Vaccines in use**: All types  **Interval**: 180 d | https://www.azernews.az/nation/184187.html |
| Belarus | No data | **Indication**: 18+  **Vaccines in use**: BBIBP-CorV, Sputnik V, Sputnik Light  **Interval**: 180-360 d | http://minzdrav.gov.by/ru/novoe-na-sayte/o-razyasnenii-poryadka-provedeniya-povtornoy-vaktsinatsii-protiv-covid-19/ |
| Belgium | **Indication**: People with diminished immunity, caused by a particular disease or by treatment | **Indication**: 18+  **Vaccines in use**: The same type in primary immunization  **Interval**: 60-120 d | https://www.health.belgium.be/fr/news/conference-interministerielle-sante-publique-0  https://www.info-coronavirus.be/en/vaccination/#faq  https://www.brussels.be/booster-dose-brussels-residents-over-18 |
| Bosnia and Herzegovina |  |  |  |
| Bulgaria | **Indication**: people with immunosuppression  **Interval**: 28 d | **Indication**: 12+  **Vaccines in use**: Comirnaty, Spikevax, Janssen COVID-19 Vaccine  **Interval**: 90 d  **Interval for COVID-19 survivals**: 180 d | https://coronavirus.bg/bg/news/251  https://worldakkam.com/bulgaria-important-information-for-citizens-wanting-to-get-booster-dose-for-janssen-vaccine-novinite-com/634593/ |
| Cyprus | No data | **Indication**: 18+  **Interval**: 180 d | http://www.news.cn/english/2021-11/15/c_1310312673.htm |
| Czechia | **Indication**: People with moderate to severe immune suppression  **Vaccines in use**: Comirnaty, Spikevax  **Interval**: 28 d | **Indication**: 12+  **Vaccines in use**: Comirnaty, Spikevax  **Interval**: 150 d | http://www.news.cn/english/2021-08/31/c_1310158121.htm  https://covid.gov.cz/en/situations/register-vaccination/booster-and-additional-dose |
| Denmark | **Indication**: People with severely impaired immune system  **Interval**: 30-270 d | **Indication**: 18+  **Interval**: 140 d  **Interval for COVID-19 survivals**: 30 d | https://www.sst.dk/da/Nyheder/2021/Personer-med-et-svaert-nedsat-immunforsvar-bliver-nu-tilbudt-en-3_-dosis-COVID-19-vaccine  https://www.sst.dk/en/English/News/2021/Everyone-aged-18-and-over-can-now-get-the-3rd-booster-vaccine-dose |
| Estonia | **Indication:** People with a weakened immune system | **Indication**: 18+  **Vaccines in use**: Comirnaty, Spikevax  **Interval**: 90 d  **Interval for COVID-19 survivals**: 150 d | https://vaktsineeri.ee/en/news/third-vaccine-doses-may-be-available-in-october/  https://vaktsineeri.ee/uudised/ekspertkomisjon-soovitab-luhendada-vaktsiinikuuri-ja-tohustusdoosi-vahelist-soovituslikku-aega/ |
| Finland | **Indication**: People with severe immunosuppression; people receiving the first and second dose less than six weeks apart  **Interval**: 60 d | **Indication**: 18+  **Interval**: 90-180 d | https://thl.fi/en/web/thlfi-en/-/thl-proposes-a-third-coronavirus-vaccine-dose-for-limited-groups  https://thl.fi/fi/web/infektiotaudit-ja-rokotukset/rokotteet-a-o/koronavirusrokotteet-eli-covid-19-rokotteet-ohjeita-ammattilaisille/kolmas-koronarokoteannos |
| France | **Indication**: People with severe immunosuppression; people receiving the first and second dose less than six weeks apart  **Interval**: 60 d | **Indication**: 12+  **Vaccines in use**: Comirnaty, Spikevax  **Interval**: 90 d  **Interval for COVID-19 survivals**: 180 d | https://vaccination-info-service.fr/Les-maladies-et-leurs-vaccins/COVID-19 |
| Georgia | No data | **Indication**: 18+  **Vaccines in use**: Comirnaty, inactivated vaccines (only for the same type in the primary vaccination)  **Interval**: 90-180 d | https://vaccines.ncdc.ge/vaccinationprocess/#who |
| Germany | **Indication**: People who may not have a sufficient or rapidly decreasing immune response after a complete vaccination, including residents of care facilities, facilities for people with disabilities, other facilities with vulnerable groups, people with immunodeficiency or immunosuppression  **Interval**: 180 d | **Indication**: 12+  **Vaccines in use**: Comirnaty, Spikevax  **Interval**: 90-180 d  **Interval for COVID-19 survivals**: 90 d | https://www.bundesgesundheitsministerium.de/coronavirus/faq-covid-19-impfung.html#c21988  https://www.rki.de/DE/Content/Kommissionen/STIKO/Empfehlungen/PM_2021-10-07.html;jsessionid=A597C1109F012BB859E304E5DFDAF898.internet101  https://www.politico.eu/article/germany-recommends-covid-19-boosters-after-3-months/ |
| Greece | No data | **Indication**: 18+  **Interval**: 90-210 d | https://www.reuters.com/world/europe/greece-offers-covid-vaccine-booster-three-months-after-initial-shots-2021-12-03/ |
| Hungary | No data | **Indication**: 18+  **Interval**: 120 d | https://koronavirus.gov.hu/cikkek/magyarorszag-az-elso-ahol-mar-harmadik-oltasra-lehet-idopontot-foglalni |
| Iceland | **Indication**: people with severe immunosuppression  **Interval**: 84 d | **Indication**: 16+  **Vaccines in use**: Comirnaty, Spikevax  **Interval**: 120 d  **Interval for COVID-19 survivals**: 180 d | https://www.covid.is/covid-19-booster-vaccinations |
| Ireland | **Indication**: People with cancer, kidney diseases, HIV, transplants, genetic diseases, High dose systemic steroids, immunocompromise; people with following treatment in the last 6 months: Cyclophosphamide, Rituximab, Alemtuzumab, Cladribine, Ocrelizumab  **Interval**: 60 d | **Indication**: 16+  **Vaccines in use**: Comirnaty, Spikevax  **Interval**: 90 d | https://www2.hse.ie/screening-and-vaccinations/covid-19-vaccine/get-the-vaccine/weak-immune-system/  <https://www2.hse.ie/screening-and-vaccinations/covid-19-vaccine/get-the-vaccine/covid-19-vaccine-booster-dose/> |
| Israel | No data | **Indication**: 12+  **Vaccines in use**: Comirnaty, Spikevax  **Interval**: 90 d | https://www.covid.is/covid-19-booster-vaccinations |
| Italy | **Indication**: people who received a solid organ transplant or who are immunocompromised  **Interval**: 28 d | **Indication**: 12+  **Vaccines in use**: Comirnaty, Spikevax  **Interval**: 120 d | https://www.aifa.gov.it/en/domande-e-risposte-su-vaccini-covid-19  https://www.salute.gov.it/portale/nuovocoronavirus/dettaglioContenutiNuovoCoronavirus.jsp?lingua=italiano&id=5452&area=nuovoCoronavirus&menu=vuoto |
| Kazakhstan | No data | **Indication**: 18+  **Interval**: 180 d | https://www.inform.kz/en/kazakhstan-expands-eligibility-for-covid-19-vaccine-booster-shots_a3872210 |
| Kyrgyzstan | No data | **Indication**: 18+  **Vaccines in use**: Comirnaty, Spikevax, BBIBP-CorV, Vaxzevria, Sputnik V  **Interval**: 180 d | https://24.kg/english/217940_COVID-19_Everyone_can_get_booster_dose_of_vaccine_in_Kyrgyzstan/ |
| Latvia | **Indication**: People with high immunosuppression  **Interval**: 28 d | **Indication**: 18+  **Interval**: 90 d  **Interval for COVID-19 survivals**: 180 d | https://www.vm.gov.lv/lv/jaunums/ari-latvija-saks-treso-vakcinu-devu-pret-covid-19-administresanu-ka-pirmie-tas-sanems-cilveki-ar-butiski-novajinatu-imuno-sistemu  https://bnn-news.com/covid-19-vaccine-booster-shots-available-three-months-after-initial-vaccination-in-latvia-231095 |
| Lithuania | **Indication**: High-risk patients with onco-hematological diseases, receiving treatment for onco-hematological diseases, on dialysis or after organ transplantation, with autoimmune diseases when receiving immunosuppressive therapy | **Indication**: 12+  **Vaccines in use**: Comirnaty, Spikevax  **Interval**: 120-180 d | <https://sam.lrv.lt/lt/naujienos/nustatyta-tvarka-kaip-trecia-doze-bus-revakcinuojami-imunosupresiniai-pacientai>  <https://koronastop.lrv.lt/lt/duk/vakcinacija-nuo-covid-19/revakcinacija#item-1054>  https://www.lrt.lt/en/news-in-english/19/1556372/getting-your-booster-shot-in-lithuania-what-you-need-to-know |
| Luxembourg | **Indication**: Immunocompromised patients | **Indication**: 12+  **Interval**: 90 d | <https://sante.public.lu/fr/espace-professionnel/recommandations/conseil-maladies-infectieuses/covid-19/covid-19-annexes/CSMI-recommandation-3eme-dose-vaccin-COVID-19-personnes-immunodeprimees.pdf>  https://msan.gouvernement.lu/en/actualites.gouvernement%2Ben%2Bactualites%2Btoutes_actualites%2Bcommuniques%2B2021%2B09-septembre%2B14-vaccin-dose-additionnelle.html  https://msan.gouvernement.lu/en/actualites.gouvernement%2Ben%2Bactualites%2Btoutes_actualites%2Bcommuniques%2B2022%2B01-janvier%2B07-vaccination-booster.html |
| Malta | **Indication**: 65+; people with immunosuppression  **Interval**: 28 d | **Indication**:18+  **Vaccines in use**: Comirnaty, Spikevax  **Interval**: 90 d | https://deputyprimeminister.gov.mt/en/health-promotion/covid-19/Pages/vaccines.aspx |
| Monaco | No data | **Indication**:18+  **Interval**: 180 d | https://covid19.mc/lutter-contre-la-covid-19/vaccination/ |
| Montenegro | **Indication**: Vulnerable people  **Interval**: 60 d | **Indication**:18+  **Vaccines in use**: Comirnaty or the same type in primary immunization  **Interval**: 150 d | https://www.covidodgovor.me/me/cesta-pitanja |
| Netherlands | **Indication**: People with immunosuppression | **Indication**:18+  **Vaccines in use**: Comirnaty, Spikevax, Janssen COVID-19 Vaccine (only when people cannot get an mRNA vaccine)  **Interval**: 90 d | https://www.rivm.nl/en/covid-19-vaccination/vaccines/immunocompromised-patients#rm-why-are-some-people-with-severely-impaired-immunity-receiving-a-third-vaccination-620821-more  https://www.government.nl/topics/coronavirus-covid-19/dutch-vaccination-programme/booster-vaccination |
| North Macedonia | No data | Indication: 18+  Interval: 180 d | https://koronavirus.gov.mk/vesti/223492 |
| Norway | **Indication**: People with immunosuppression | **Indication**: 18+  **Interval**: 140 d | https://www.fhi.no/publ/brev/ytterligere-informasjon-om-en-tredje-dose-til-de-med-alvorlig-nedsatt-immun/  https://www.fhi.no/en/news/2021/more-people-will-be-offered-a-booster-dose/ |
| Poland | **Indication**: 12+ with immunosuppression  **Interval**: 28 d | **Indication**: 12+  **Vaccines in use**: Comirnaty, Spikevax, Janssen COVID-19 Vaccine  **Interval**: 60-150 d | https://www.gov.pl/web/zdrowie/komunikat-nr-12-ministra-zdrowia-w-sprawie-szczepien-przeciw-covid-19-dawka-przypominajaca-oraz-dawka-dodatkowa-uzupelniajaca-schemat-podstawowy  https://notesfrompoland.com/2021/10/25/poland-to-launch-covid-booster-jabs-for-all-adults-next-week/ |
| Portugal | **Indication**: People with immunosuppression | **Indication**: 18+  **Interval**: 90 d | https://covid19.min-saude.pt/terceira-dose-comeca-a-ser-administrada-a-idosos-com-mais-de-65-anos-e-residentes-em-lares/  https://www.theportugalnews.com/news/2022-01-17/booster-jabs-now-available-for-over-40s/64673 |
| Republic of Moldova | **Indication**: people with immunosuppression  **Interval**: 28 d | **Indication**: 18+  **Interval**: 56-180 d | https://vaccinare.gov.md/news/15-implementarea-procesului-de-vaccinare-impotriva-covid-19-in-republica-moldova |
| Romania | No data | **Indication**: 12+  **Vaccines in use**: Comirnaty, Spikevax, Janssen COVID-19 Vaccine (only for the same type in the primary vaccination)  **Interval**: 120 d | https://www.romania-insider.com/ro-covid-booster-interval-reduced-jan-2022 |
| Russia | No data | **Indication**: 18+  **Interval**: 180 d | https://www.reuters.com/business/healthcare-pharmaceuticals/moscow-begins-booster-vaccine-campaign-russias-covid-19-cases-surge-2021-07-01/ |
| San Marino | No data | **Indication**: 12+  **Vaccines in use**: Comirnaty for mRNA vaccines in the primary vaccination, Sputnik Light for Sputnik V in the primary vaccination  **Interval**: 180 d | https://www.iss.sm/on-line/home/aggiornamenti-coronavirus/articolo49015783.html |
| Serbia | No data | **Indication**: 18+  **Interval**: 180 d | https://www.srbija.gov.rs/vest/en/177106/third-dose-of-covid-19-vaccine-available-as-of-tomorrow.php |
| Slovakia | No data | **Indication**: 18+  **Interval**: 90 d | https://www.inform.kz/en/health-ministry-booster-shots-to-be-available-in-slovakia-after-three-months_a3874527 |
| Slovenia | **Indication**: 70+ and particularly vulnerable chronic patients | **Indication**: 12+  **Vaccines in use**: Comirnaty, Spikevax  **Interval**: 270 d | https://www.cepimose.si/cepljenje-proti-covidu-19/pogosta-vprasanja-in-odgovori/  https://sloveniatimes.com/booster-shot-needed-for-indefinite-validity-of-covid-pass/ |
| Spain | **Indication**: People with immunosuppression; 40+ with Down Syndrome  **Interval**: 28 d | **Indication**: 18+  **Vaccines in use**: Comirnaty, Spikevax  **Interval**: 90 d | https://www.vacunacovid.gob.es/preguntas-y-respuestas/quienes-recibiran-una-dosis-adicional-de-la-vacuna  https://www.thelocal.es/20220113/spain-approves-covid-19-booster-shots-for-all-people-over-18/ |
| Sweden | **Indication**: 18+ with severe immunosuppression  **Interval**: 56 d | **Indication**: 18+  **Interval**: 90 d  **Interval for COVID-19 survivals**: After recovery | https://www.folkhalsomyndigheten.se/smittskydd-beredskap/utbrott/aktuella-utbrott/covid-19/vaccination-mot-covid-19/fragor-och-svar-om-vaccination-mot-covid-19/  https://www.reuters.com/world/europe/sweden-cuts-recommended-gap-between-second-third-covid-shot-2022-01-12/ |
| Switzerland | **Indication**: People with severe immunosuppression | **Indication**: 12+  **Interval**: 120 d | https://www.ge.ch/en/getting-vaccinated-against-covid-19/faq-vaccination-against-covid-19  https://www.bag.admin.ch/bag/de/home/krankheiten/ausbrueche-epidemien-pandemien/aktuelle-ausbrueche-epidemien/novel-cov/information-fuer-die-aerzteschaft/covid-19-impfung.html#-1942803447 |
| Tajikistan | No data | **Indication**: healthcare workers, teachers, bank staff, people with chronic non-communicable diseases | https://www.ozodi.org/a/31652717.html |
| Turkey | No data | **Indication**: 18+  **Vaccines in use**: All types  **Interval**: 90 d | https://covid19asi.saglik.gov.tr/EN-78316/frequently-asked-questions.html?Sayfa=1  https://www.dailysabah.com/turkey/turkey-starts-offering-5th-dose-of-covid-19-booster-shots/news |
| Ukraine | No data | **Indication**: 18+  **Vaccines in use**: Comirnaty, Spikevax  **Interval**: 180-270 d | https://visitukraine.today/blog/69/booster-dose-revaccination-was-allowed-in-ukraine |
| United Kingdom | **Indication**: People with immunosuppression  **Interval**: 60 d | **Indication**: 16+; 12-15 can get a booster dose if they have a condition that puts them at high risk from COVID-19 or they live with someone who has a weakened immune system  **Vaccines in use**: Comirnaty, Spikevax  **Interval**: 90 d  **Interval for COVID-19 survivals**: 28 d | https://www.gov.uk/government/news/jcvi-issues-updated-advice-on-covid-19-booster-vaccination  https://www.nhs.uk/conditions/coronavirus-covid-19/coronavirus-vaccination/coronavirus-booster-vaccine/ |
| Uzbekistan | No data | **Indication**: Yes | https://m.akipress.com/news:664935:Uzbekistan_starts_COVID-19_booster_vaccination/ |
| **South-East Asia** | | | |
| Bangladesh | No data | **Indication**: Front liners, 50+  **Interval**: 180 d | https://newsonair.com/2022/01/17/bangladesh-reduces-the-minimum-age-for-covid-booster-vaccine-to-50-2/ |
| Bhutan | No data | **Indication**: overseas travelers, health workers, people with underlying conditions, essential workers and people living in nursing homes; 65+ | https://news.trust.org/item/20211224100000-l4re3/ |
| India | No data | **Indication**: HCW, essential workers, 60+ with underlying conditions | https://www.cowin.gov.in |
| Indonesia | No data | **Indication**: 18+  **Vaccines in use**: For the primary Sinovac vaccine or the first and second doses of Sinovac vaccine, half a booster dose of Pfizer or AstraZeneca will be given. For the primary AstraZeneca vaccine or the first and second doses of AstraZeneca vaccine, a half dose of Moderna will be given booster vaccine  **Interval**: 180 d | https://www.kemkes.go.id/article/view/21080200001/kemenkes-tegaskan-vaksinasi-booster-hanya-untuk-tenaga-kesehatan.html  https://www.kemkes.go.id/article/view/22011200002/vaksinasi-booster-gratis-dimulai-12-januari-2022.html |
| Maldives | No data | **Indication**: 18+  **Interval**: 180 d | https://edition.mv/news/23559 |
| Nepal | No data | **Indication**: Essential workers, 60+ | https://thehimalayantimes.com/nepal/covid-19-booster-vaccine-to-be-administered-from-sunday |
| Sri Lanka | No data | **Indication**: 20+  **Vaccines in use**: Comirnaty  **Interval**: 90 d | http://www.xinhuanet.com/asiapacific/20220128/ead34680a4ef4b85ba760526a24f8ef4/c.html |
| Thailand | No data | **Indication**: 18+  **Vaccines in use**: Comirnaty, Spikevax, Vaxzevria  **Interval**: 90 d | https://www.thaipbsworld.com/thailand-to-speed-up-vaccine-booster-shots/ |
| Timor-Leste | No data | **Indication**: People with underlying conditions, essential workers, 60+ | http://www.tatoli.tl/en/2022/01/03/timor-leste-to-introduce-covid-19-booster-jabs/ |
| **Western Pacific** | | | |
| Australia | **Indication**: 5+ with severe immunosuppression  **Interval**: 60-180 d | **Indication**: 16+  **Vaccines in use**: Comirnaty, Spikevax, Vaxzevria  **Interval**: 90 d | https://www.health.gov.au/ministers/the-hon-greg-hunt-mp/media/booster-shot-for-severely-immunocompromised-australians  https://www.health.gov.au/initiatives-and-programs/covid-19-vaccines/getting-your-vaccination/booster-doses |
| Brunei | No data | **Indication**: 18+  **Vaccines in use**: Comirnaty  **Interval**: 90 d | https://www.thestar.com.my/aseanplus/aseanplus-news/2021/12/14/covid-19-booster-shots-to-open-for-those-18-and-above-in-brunei |
| Cambodia | No data | **Indication**: 18+  **Vaccines in use**: CoronaVac  **Interval**: 120 d | https://www.reuters.com/business/healthcare-pharmaceuticals/colombia-reduces-booster-vaccine-waiting-time-four-months-2022-01-12/ |
| China | No data | **Indication:** 18+  **Vaccines in use**: The same type in primary immunization  **Interval**: 180 d | http://www.gov.cn/xinwen/2021-10/27/content_5645087.htm |
| Cook Islands | No data | **Indication:** 18+  **Vaccines in use**: Comirnaty  **Interval**: 120 d | https://covid19.gov.ck/sites/default/files/2022-02/Media%20Release%20-%20Vaccination%20Roll-Out%20Update%206%20February%20Final.pdf |
| Fiji | No data | **Indication:** 18+  **Vaccines in use**: Comirnaty  **Interval**: 150 d | http://www.news.cn/english/2021-12/22/c_1310388167.htm |
| Japan | **Interval**: 240 d | **Indication:** 18+  **Vaccines in use**: Comirnaty, Spikevax  **Interval**: 180 d | https://www.mhlw.go.jp/content/10601000/000833964.pdf  https://www.mhlw.go.jp/stf/seisakunitsuite/bunya/vaccine_booster.html |
| Laos | No policy | **Indication**: HCW | http://www.news.cn/english/asiapacific/2021-11/09/c_1310300238.htm |
| Malaysia | No data | **Indication**: 18+  **Vaccines in use**: Comirnaty, Spikevax, CoronaVac  **Interval**: 90 d | https://covid-19.moh.gov.my/semasa-kkm/2021/10/kelulusan-bersyarat-pemberian-dos-penggalak-booster-dose  https://www.straitstimes.com/asia/se-asia/malaysia-lifts-african-travel-ban-cuts-booster-wait-amid-omicron-fears |
| Micronesia | No data | **Indication**: 18+  **Vaccines in use**: Comirnaty, Spikevax, Janssen COVID-19 Vaccine  **Interval**: 180 d | https://fsmembassy.fm/what-the-fsm-knows-about-the-omicron-variant-of-covid-19-how-the-fsm-is-responding-to-it-best-advice-for-citizens-get-vaccinated-get-boosted/ |
| Mongolia | No data | **Indication**: 18+  **Interval**: 90 d  **Interval for COVID-19 survivals**: 21 d | https://www.facebook.com/watch/?v=182975560609936  https://akipress.com/news:663617:Mongolia_vaccinates_359,447_people_with_3rd_dose_of_coronavirus_vaccine/ |
| New Zealand | **Indication**: people with severe immunosuppression  **Interval**: 56 d | **Indication**: 18+  **Vaccines in use**: The same type in primary immunization  **Interval**: 90 d | https://www.health.govt.nz/our-work/diseases-and-conditions/covid-19-novel-coronavirus/covid-19-vaccines/covid-19-vaccine-health-advice/covid-19-vaccine-severely-immunocompromised-people  https://www.health.govt.nz/our-work/diseases-and-conditions/covid-19-novel-coronavirus/covid-19-vaccines/covid-19-vaccine-boosters |
| Niue | No data | **Indication**: 18+  **Vaccines in use**: Comirnaty  **Interval**: 120 d | https://www.facebook.com/page/102108048101682/search/?q=booster |
| Palau | **Indication**: People with immunosuppression | **Indication**: 18+  **Interval**: 180 d | http://www.palauhealth.org/2019nCoV/PR-old/MHHS%20PSA%20VACCINATION%203RD%20DOSE%2009092021.pdf  http://www.palauhealth.org/2019nCoV/PR-old/MHHS%20PSA%20VACC%20SCHEDULE%2002042022.pdf |
| Papua New Guinea | No data | **Indication**: 18+  **Vaccines in use**: Janssen COVID-19 Vaccine or the same type in primary immunization  **Interval**: 60-180 d | https://www.facebook.com/photo?fbid=265099225750612&set=pcb.265100749083793 |
| Philippines | No data | **Indication**: 18+  **Interval**: 90 d | https://doh.gov.ph/press-release/DOH-SHORTER-INTERVAL-FOR-COVID-19-VACCINE-BOOSTERS |
| Singapore | **Indication**: People with immunosuppression; people receiving Sinovac | **Indication**: 12+  **Vaccines in use**: Comirnaty, Spikevax  **Interval**: 150 d  **Interval for COVID-19 survivals**: 90 d | https://www.moh.gov.sg/news-highlights/details/updates-to-healthcare-protocols-and-implementation-of-vaccine-booster-strategy_10Sep2021  https://www.moh.gov.sg/covid-19/vaccination/faqs---booster-doses |
| South Korea | No data | **Indication**: 18+  **Interval**: 90 d | https://www.reuters.com/world/asia-pacific/skorea-cut-covid-19-booster-shot-interval-again-infections-rise-2021-12-10/ |
| Samoa | No data | **Indication**: 18+ | https://samoaglobalnews.com/samoa-to-roll-out-covid-19-booster-shots/ |
| Solomon Islands | No data | **Indication**: Essential workers | https://www.facebook.com/officialmhmssi/posts/223374359966629 |
| Tonga | No data | **Indication**: Essential workers  **Vaccines in use**: Comirnaty | https://www.facebook.com/mohtonga/posts/339635561322313 |
| Tuvalu | No policy | No policy |  |
| Vanuatu | No policy | No policy |  |
| Vietnam | **Indication**: people with immunosuppression and the elderly  **Interval**: 28 d | **Indication**: 18+  **Vaccines in use**: mRNA vaccines or the same type in primary immunization  **Interval**: 90 d | https://vietnamnews.vn/society/1108544/booster-shots-eligible-after-three-months-from-second-covid-19-vaccine-dose-health-ministry.html |

Abbreviation: d, days.

### Table S4. Country lists of selling/donating or receiving COVID-19 vaccines

| **Country role** | **Country lists** |
| --- | --- |
| Selling or donating countries | Australia; Bahrain; Bhutan; Canada; China; Colombia; Iceland; Japan; Korea; Kuwait; Mauritius; Mexico; Monaco; New Zealand; Norway; Oman; The Philippines; Qatar; Saudi Arabia; Singapore; Switzerland; Austria; Belgium; Croatia; Denmark; Estonia; Finland; France; Germany; Greece; Ireland; Italy; Luxembourg; Malta; Netherlands; Poland; Portugal; Spain; Sweden; United Kingdom; United States; Vietnam; United Arab Emirates. |
| Recipient countries | Afghanistan; Albania; Algeria; Andorra; Angola; Antigua and Barbuda; Argentina; Armenia; Australia; Azerbaijan; Bahamas; Bahrain; Bangladesh; Barbados; Belize; Benin; Bhutan; Bolivia; Bosnia and Herzegovina; Botswana; Brazil; Brunei Darussalam; Burkina Faso; Cabo Verde; Cambodia; Cameroon; Canada; Central African Republic; Chad; Chile; China; Colombia; Comoros; Congo, Dem. Rep.; Congo, Rep.; Costa Rica; Cote d'Ivoire; Djibouti; Dominica; Dominican Republic; Ecuador; Egypt, Arab Rep.; El Salvador; Eswatini; Ethiopia; Fiji; Gabon; Gambia, The; Georgia; Ghana; Grenada; Guatemala; Guinea; Guinea-Bissau; Guyana; Haiti; Honduras; India4; Indonesia; Iran; Iraq; Israel; Jamaica; Jordan; Kenya; Kiribati; Korea, Dem. People’s Rep.; Kosovo; Kuwait; Kyrgyz Republic; Lao PDR; Lebanon; Lesotho; Liberia; Libya; Madagascar; Malawi; Malaysia; Maldives; Mali; Mauritania; Mauritius; Mexico; Micronesia; Moldova; Monaco; Mongolia; Montenegro; Morocco; Mozambique; Myanmar; Namibia; Nauru; Nepal; New Zealand; Nicaragua; Niger; Nigeria; North Korea; North Macedonia; Oman; Pakistan; Panama; Papua New Guinea; Paraguay; Peru; Philippines; Qatar; Rwanda; Samoa; Sao Tome and Principe; Saudi Arabia; Senegal; Serbia; Sierra Leone; Singapore; Solomon Islands; Somalia; South Africa; South Sudan; South Korea; Sri Lanka; St. Kitts and Nevis; St. Lucia; St. Vincent and the Grenadines; Sudan; Suriname; Syria; Tajikistan; Timor-Leste; Togo; Tonga; Trinidad and Tobago; Tunisia; Tuvalu; Uganda; Ukraine; United Arab Emirates; United Kingdom; and Northern Ireland; Tanzania; Uruguay; Uzbekistan; Vanuatu; Venezuela; Vietnam; West Bank and Gaza; Yemen, Rep.; Zambia; Zimbabwe. |

The data are derived from COVAX (https://www.gavi.org/covax-facility).

### Figure S1. The distribution of whether local residents need to pay for vaccine.

Note: Singaporeans need to charge if they want to choose an inactivated vaccine as a booster, but not if they choose mRNA vaccine.


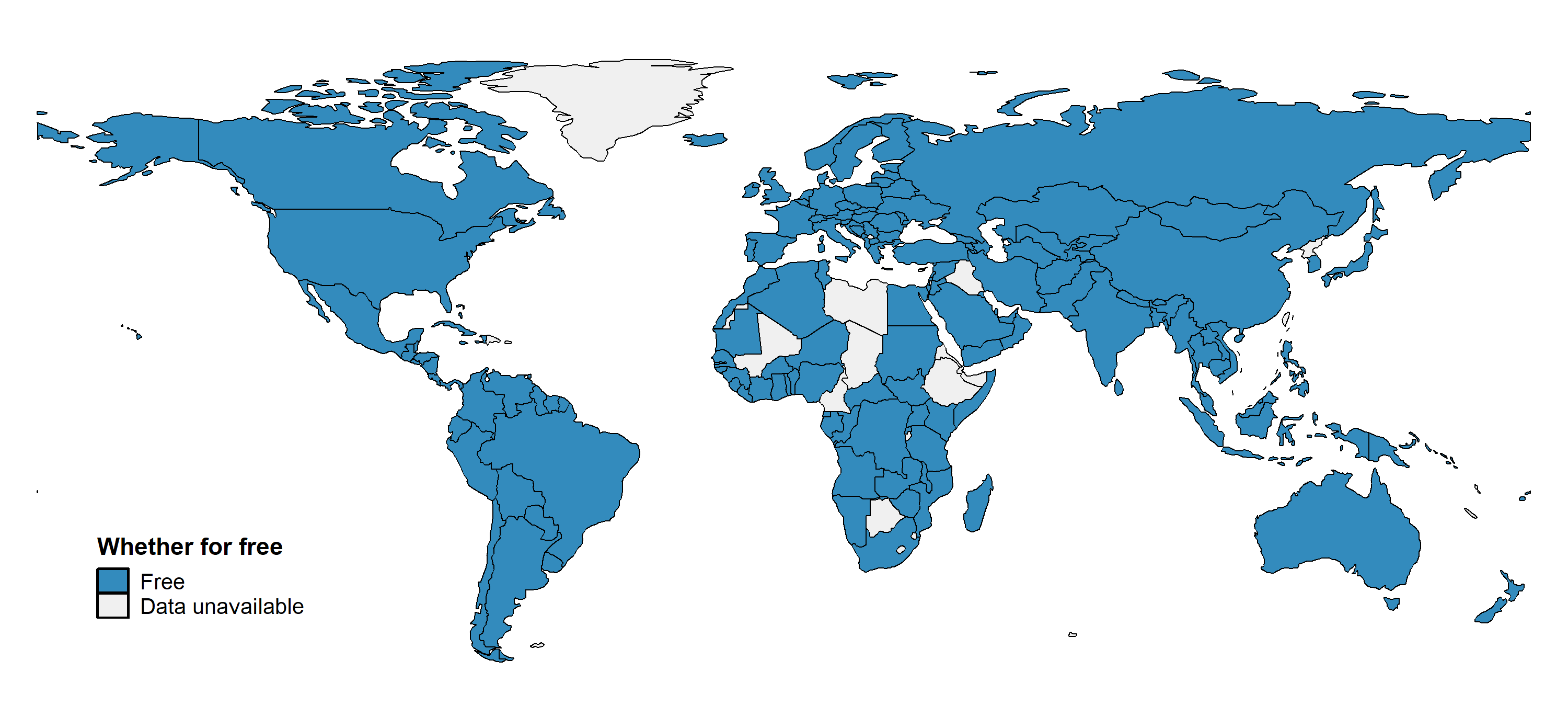


### Figure S2. Geographic distribution of overall technical platforms for vaccines

Since the conjugate vaccine was only used in three countries (Cuba, Iran and Nicaragua), it is not shown in the map.


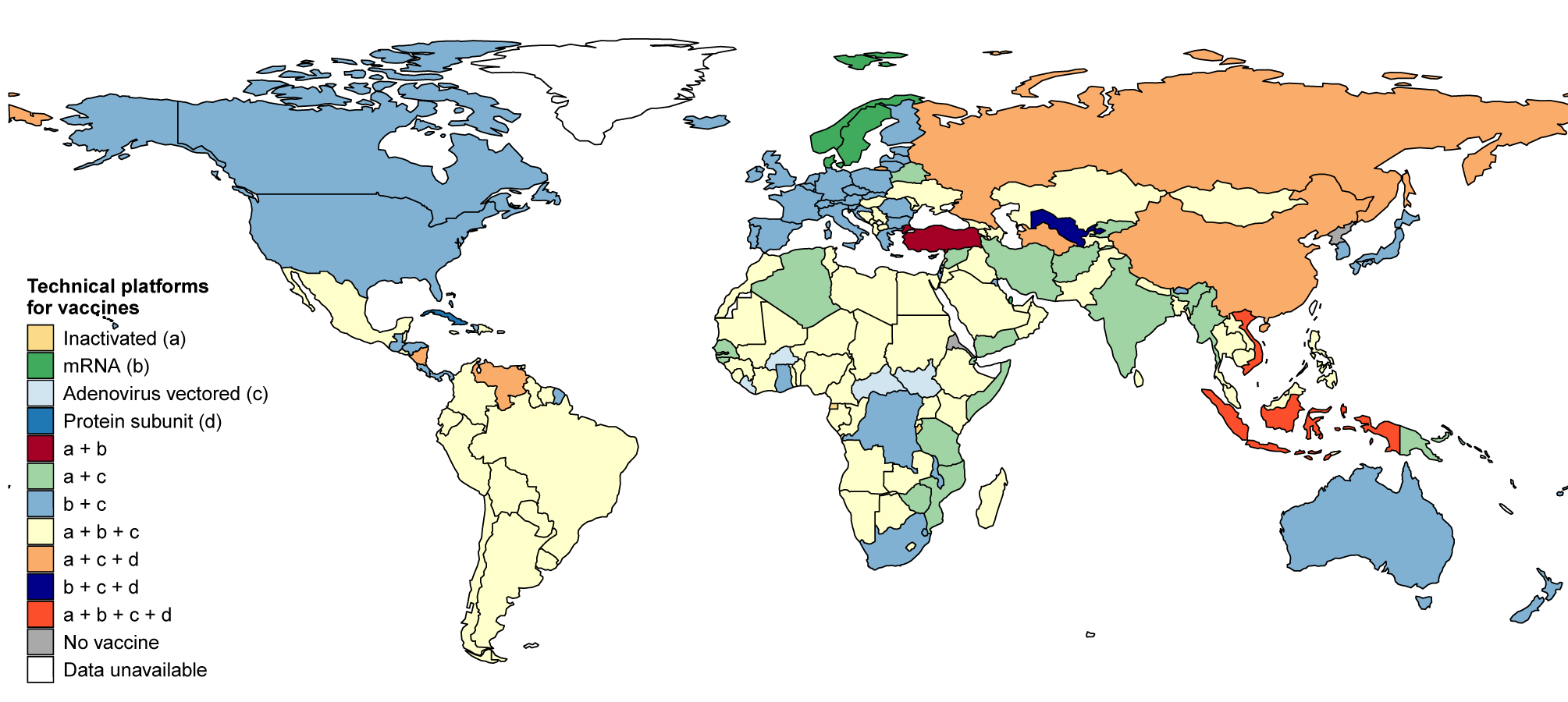


# Target population for primary vaccination

## *Method to estimate the size of target population*

**Metrices**

Some metrices have been collected to estimate target population: 1) **Total population estimates**. The age-specific population estimates by country in 2020 were mainly derived from the UN World Population Prospects, supplemented by the WorldPop datasets for some countries with age-specific proportion unavailable. 2) **The indication groups for primary vaccination**. The population groups who are eligible for a COVID-19 vaccine, recommended by governments/health departments. Indication groups varied by countries, which might be general population with a certain age group or some specific population groups, e.g., frontline workers. 3) **The contraindication groups**. The population who should not get vaccinated against COVD-19 recommended by governments/health departments, e.g., pregnant women, people with certain underlying conditions (i.e., bleeding disorders and immune suppression), and those previously infected with SARS-CoV-2. They should be excluded from the indication groups according to the vaccination policy in each country.

**Definition of** **special population groups**

Two underlying conditions are considered in this study: bleeding disorders and immune suppression. A bleeding disorder is defined as condition in which the blood’s ability to clot is impaired or at thrombotic states. Diseases that would cause bleeding disorder are considered as a bleeding problem. In the case of immune suppression, we include categories of cancers with direct immune suppression and cancers with possible immune suppression (from treatment therapy), as well as HIV/AIDS without receiving antiretroviral therapy (ART) based on Clark’s method. The categories and definitions of special population groups are presented in Supplementary Table 5, in which specific causes of bleeding disorders and immune suppression defined by GBD study group were listed.

**Assumptions for calculating the size of special population groups**

For each country, we calculated the size of target population through excluded those who aren’t allowed to receive vaccines based on a country’s immunization strategy. We assumed that the individuals belonging to the contraindication lists cannot be vaccinated, in which the size was added together. There are some assumptions made for calculating the size of special population groups. First, we assumed that countries with data regarding ART coverage among people living with HIV unavailable are replaced with average of regional data. For example, we replaced ART coverage in Sweden with average value in Europe Region (71.0%). In addition, for some country-level data for specific groups that are not available, we excluded them in the calculation by assuming that it has little impact on the calculation of target population due to their small size.

## *Supplementary results*

### Table S5. Categories and definitions of special population groups belonging to indication and contraindication lists

| **Population groups** | **Definition** | **Size data available** | **Data source** |
| --- | --- | --- | --- |
| **Pregnancy-related people** |  |  |  |
| Pregnant women | - | Yes | Wang W et al, BMJ |
| Breastfeeding (lactation) women | - | No | - |
| Plan to get pregnancy | - | No | - |
| **People working at potentially greater risk of SARS-CoV-2 infection** |  |  |  |
| Healthcare workers | Including healthcare workers, nurses and midwives | Yes | Wang W et al, BMJ |
| People maintaining society safety and national security | Police and military | Yes | Wang W et al, BMJ |
| Other essential workers | People engaging in electricity, gas, water, food, steam, air conditioning, and accommodation supply; participating in sewerage, waste management, and remediation activities; involved in domestic transportation and storage | Yes | Wang W et al, BMJ |
| **People at higher risk of severe COVID-19 disease** |  |  |  |
| Residents living in nursing homes/long-care settings or receiving home care | Elderly people receiving long-term care at a residential care facility and at home | Partially | <https://www.who.int/data/maternal->  newborn-child-adolescent-ageing/indicator-explorer-  new/mca/percentage-of-older-  people-receiving-long-term-care-  at-a-residential-care-facility-and-at-home |
| Vulnerable people with high risk of experiencing irreversible and devastating harm from COVID-19 due to their health conditions, which included the elderly and people with underlying medical conditions. | Underlying conditions included cardiovascular disease, chronic kidney disease, chronic respiratory disease, chronic liver disease, diabetes, cancer with direct immunosuppression, cancer without direct immunosuppression but with possible immunosuppression caused by treatment, HIV or AIDS, tuberculosis (excluding latent infections), chronic neurological disorders, and sickle cell disorders. And target population in this category was classified into (1) people younger than 60 years with at least one underlying condition; (2) people aged 60 years or older with at least one underlying conditions; (3) people aged 80 years or older without any underlying conditions. | Yes | Clark A et al. Lancet GH  Wang W et al, BMJ |
| People with (moderate to severe) immunosuppression | **Cancers with direct immune suppression:** Hodgkin lymphoma; Non-Hodgkin lymphoma; Multiple myeloma; Acute lymphoid leukemia; Chronic lymphoid leukemia; Acute myeloid leukemia; Chronic myeloid leukemia; Other leukemia; Other malignant neoplasms; Myelodysplastic, myeloproliferative, and other hematopoietic neoplasms  **Cancers with possible immune suppression (from treatment therapy):** Lip and oral cavity cancer; Nasopharynx cancer; Other pharynx cancer; Esophageal cancer; Stomach cancer; Colon and rectum cancer; Liver cancer due to hepatitis B; Liver cancer due to hepatitis B; Liver cancer due to hepatitis B; Liver cancer due to hepatitis C; Liver cancer due to alcohol use; Liver cancer due to NASH; Liver cancer due to other causes; Gallbladder and biliary tract cancer; Pancreatic cancer; Larynx cancer; Tracheal, bronchus, and lung cancer; Malignant skin melanoma; Breast cancer; Cervical cancer; Uterine cancer; Ovarian cancer; Prostate cancer; Testicular cancer; Kidney cancer; Bladder cancer; Brain and nervous system cancer; Thyroid cancer; Mesothelioma  **HIV/AIDS without receiving ART** | Yes | Estimated used data from GBD 2019 |
| People with bleeding disorders | Other nutritional deficiencies; Acute hepatitis A; Acute hepatitis B; Acute hepatitis C; Acute hepatitis E; Liver cancer due to hepatitis B; Liver cancer due to hepatitis C; Liver cancer due to alcohol use; Liver cancer due to other causes; Liver cancer due to NASH; Cirrhosis and other chronic liver diseases due to hepatitis B; Cirrhosis and other chronic liver diseases due to hepatitis C; Cirrhosis and other chronic liver diseases due to alcohol use; Cirrhosis and other chronic liver diseases due to other causes; Cirrhosis and other chronic liver diseases due to NAFLD; Endocrine, metabolic, blood, and immune disorders; Dengue; Yellow fever; Multiple myeloma; Acute lymphoid leukemia; Chronic lymphoid leukemia; Acute myeloid leukemia; Chronic myeloid leukemia; Other leukemia; Myelodysplastic, myeloproliferative, and other hematopoietic neoplasms; Venomous animal contact | Yes | Estimated used data from GBD 2019 |
| People on dialysis | - | No | - |
| **Others** |  |  |  |
| International travelers | - | No | - |
| People previously infected with SARS-CoV-2 | COVID-19 cumulative cases | Yes | WHO COVID-19 dashboard |
| People receiving vaccines from Janssen & Janssen, Sinovac or Sinopharm | - | Partially, and was available in Chile and Uruguay |  |

**Estimate peoples of population size with bleeding disorders and immune suppression**

Since the prevalence of the sub-categories of each a disease (bleeding disorders or immune suppression) was separately reported by GBD, one person can simultaneously suffer more than one sub-category of such disease, namely multimobidity. Thus, we estimated the number of people who suffered at least one bleeding disorders- or immune suppression-related conditions, rather than directly adding the number of sub-categories of bleeding disorders or immune suppression.

We mainly adapted the Clark’s method to estimate the number of individuals with bleeding disorders or immune suppression. Here, we briefly explain this process. First, data on the prevalence (***p***) of sub-categories of bleeding disorders or immune suppression were extracted by age and country from GBD 2019. Then, the expected proportion of individuals with at least one bleeding problems or at least one immune suppression problems were estimated, which we refer to ***e*** here. **e** _bleeding_ for bleeding problems was acquired by 1 minus the probability of not having a condition in any of the 26 bleeding problems c _bleeding i_: 1 – [1 – p(c _bleeding1_)]×[1 – p(c _bleeding2_)]×…×[1 – p(c _bleeding27_)]; and **e** _immuno_ for immunosuppression was acquired by 1 minus the probability of not having a condition in any of the 40 immunosuppression problems c _immunoi_: 1 – [1 – p(c _immuno1_)]×[1 – p(c _immuno2_)]×…×[1 – p(c _immuno40_)]. Subsequently, the observed proportion (P) of people with at least one underlying condition was calculated by P = ***e*** × **r**, where ***r*** was the ratio between the observed and expected percentage of individuals with at least one condition, with details in Clark et al.

14 types of sub-diseases are considered as both bleeding disorders and immune suppression, including multiple myeloma, acute lymphoid leukemia, chronic lymphoid leukemia, acute myeloid leukemia, chronic myeloid leukemia, other leukemia, myelodysplastic, myeloproliferative, other hematopoietic neoplasms, liver cancer due to hepatitis B, liver cancer due to hepatitis C, liver cancer due to alcohol use, liver cancer due to other causes, and liver cancer due to nonalcoholic steatohepatitis (NASH). When calculating the proportion of individuals with at least one underlying conditions for these sub-diseases, we counted them once: (1 – [1 – p (c _bleeding1_)] × [1 – p (c _bleeding2_)] ×…× [1 – p (c _bleeding26_)] × [1 – p (c _immuno1_)] × [1 – p (c _immuno2_)] ×…× [1 – p (c _immuno26_)]) × r.

**Estimate population size of target population**

We calculated the country-specific number of contraindication groups by adding the size of pregnant women, people with certain underlying conditions, and those previously infected with SARS-CoV-2 according to the national immunization policy. Then, we estimated the size of target population for primary immunization by subtracting the population of contraindications group from the population of indication groups.

### Table S6. Global, regional, and national target population (TP).

| **Locations** | **No. of total population (million)** | **TP for primary immunization (million)** | **TP for additional immunization (million)** | **TP for booster immunization (million)** |
| --- | --- | --- | --- | --- |
| Global | | | | |
| Total | 7750.0 | 6223.1 | 45.5 | 4182.9 |
| WHO regions | | | | |
| AFR | 1120.2 | 650.3 | 2.5 | 241.4 |
| AMR | 1018.1 | 894.9 | 15.6 | 685.9 |
| EMR | 725.7 | 522.9 | 1.0 | 361.5 |
| EUR | 932.9 | 798.7 | 17.0 | 744.1 |
| SEAR | 2021.4 | 1524.5 | 0.0 | 651.2 |
| WPR | 1931.7 | 1831.7 | 9.3 | 1498.8 |
| Country | | | | |
| Afghanistan | 38.9 | 19.8 | - | - |
| Albania | 2.9 | 2.3 | 0.0 | 2.3 |
| Algeria | 43.9 | 28.6 | - | - |
| Andorra | 0.1 | 0.1 | 0.0 | 0.1 |
| Angola | 32.9 | 20.1 | - | 15.4 |
| Antigua and Barbuda | 0.1 | 0.1 | - | 0.1 |
| Argentina | 45.2 | 42.2 | 0.6 | 32.0 |
| Armenia | 3.0 | 2.5 | - | 2.2 |
| Australia | 25.5 | 23.8 | 0.8 | 20.3 |
| Austria | 9.0 | 8.6 | 0.6 | 8.0 |
| Azerbaijan | 10.1 | 8.0 | - | 7.4 |
| Bahrain | 0.4 | 1.6 | - | 1.4 |
| Bangladesh | 1.7 | 129.7 | - | 29.3 |
| Barbados | 164.7 | 0.2 | - | 0.2 |
| Belarus | 0.3 | 7.9 | - | 7.5 |
| Belgium | 9.4 | 11.0 | 0.3 | 9.2 |
| Belize | 11.6 | 0.3 | - | 0.1 |
| Benin | 0.4 | 7.9 | - | - |
| Bhutan | 12.1 | 0.6 | - | 0.1 |
| Bolivia | 0.8 | 10.5 | - | 7.5 |
| Bosnia and Herzegovina | 11.7 | 2.9 | - | - |
| Botswana | 3.3 | 1.7 | - | 1.4 |
| Brazil | 2.4 | 198.1 | 1.8 | 159.0 |
| Brunei | 212.6 | 0.4 | - | 0.3 |
| Bulgaria | 0.4 | 6.6 | 0.2 | 6.2 |
| Burkina Faso | 6.9 | 10.2 | - | - |
| Burundi | 20.9 | 5.8 | - | - |
| Cambodia | 11.9 | 14.9 | - | 10.7 |
| Cameroon | 0.6 | 17.3 | - | - |
| Canada | 16.7 | 35.7 | 1.2 | 30.6 |
| Cape Verde | 26.5 | 0.4 | - | 0.3 |
| Central African Republic | 37.7 | 2.4 | - | - |
| Chad | 4.8 | 7.6 | - | - |
| Chile | 16.4 | 17.7 | - | 16.2 |
| China | 19.1 | 1389.8 | - | 1135.2 |
| Colombia | 1439.3 | 47.2 | 0.5 | 42.0 |
| Comoros | 50.9 | 0.6 | - | - |
| Congo | 0.9 | 2.9 | - | - |
| Cook Islands | 5.5 | 0.0 | - | 0.0 |
| Costa Rica | 0.0 | 4.7 | - | 1.0 |
| Cote d'Ivoire | 5.1 | 13.6 | - | - |
| Croatia | 26.4 | 3.9 | - | - |
| Cuba | 4.1 | 11.1 | - | 11.1 |
| Cyprus | 11.3 | 1.1 | - | 1.0 |
| Czech Republic | 1.2 | 10.1 | 0.3 | 9.4 |
| Democratic Republic of the Congo | 10.7 | 42.6 | - | - |
| Denmark | 89.6 | 5.5 | 0.2 | 4.6 |
| Djibouti | 5.8 | 0.6 | - | - |
| Dominica | 1.0 | 0.1 | - | 0.0 |
| Dominican Republic | 0.1 | 8.5 | - | 7.3 |
| Ecuador | 10.8 | 16.0 | 0.1 | 11.9 |
| Egypt | 17.6 | 73.2 | - | 73.2 |
| El Salvador | 102.3 | 5.8 | - | 4.4 |
| Equatorial Guinea | 6.5 | 0.8 | - | - |
| Eritrea | 1.4 | - | - | - |
| Estonia | 3.5 | 1.3 | 0.0 | 1.1 |
| Eswatini | 1.3 | 0.8 | - | 0.6 |
| Ethiopia | 1.2 | 77.2 | - | - |
| Federated States of Micronesia | 115.0 | 0.1 | - | 0.1 |
| Fiji | 0.9 | 0.7 | - | 0.6 |
| Finland | 5.5 | 5.3 | 0.2 | 4.5 |
| France | 65.3 | 51.9 | 2.0 | 56.1 |
| Gabon | 2.2 | 1.3 | - | - |
| Georgia | 2.4 | 3.3 | - | 3.1 |
| Germany | 4.0 | 74.4 | 2.8 | 74.4 |
| Ghana | 83.8 | 19.5 | - | 5.9 |
| Greece | 31.1 | 10.0 | - | 8.7 |
| Grenada | 10.4 | 0.1 | - | 0.1 |
| Guatemala | 0.1 | 12.7 | - | 13.1 |
| Guinea | 17.9 | 8.5 | - | - |
| Guinea-Bissau | 13.1 | 1.3 | - | 1.0 |
| Guyana | 2.0 | 0.7 | - | 0.5 |
| Haiti | 0.8 | 7.0 | - | - |
| Honduras | 11.4 | 7.9 | 0.0 | 6.3 |
| Hungary | 9.9 | 9.2 | - | 8.6 |
| Iceland | 9.7 | 0.3 | 0.0 | 0.3 |
| India | 0.3 | 994.4 | - | 359.2 |
| Indonesia | 1380.0 | 244.9 | - | 188.6 |
| Iran | 273.5 | 76.4 | - | 59.8 |
| Iraq | 84.0 | 27.7 | - | 10.1 |
| Ireland | 40.2 | 4.6 | 0.1 | 3.8 |
| Israel | 4.9 | 7.8 | - | 6.7 |
| Italy | 8.7 | 58.1 | 2.5 | 54.3 |
| Jamaica | 60.5 | 2.4 | - | 2.1 |
| Japan | 3.0 | 114.1 | - | 107.3 |
| Jordan | 126.5 | 7.3 | - | 6.2 |
| Kazakhstan | 10.2 | 14.2 | - | 12.6 |
| Kenya | 18.8 | 33.0 | - | 29.3 |
| Kiribati | 53.8 | 0.1 | - | - |
| Kuwait | 0.1 | 4.0 | - | 3.3 |
| Kyrgyzstan | 4.3 | 3.9 | - | 4.1 |
| Laos | 6.5 | 5.4 | - | 0.0 |
| Latvia | 7.3 | 1.8 | 0.0 | 1.5 |
| Lebanon | 1.9 | 6.3 | 0.1 | 5.4 |
| Lesotho | 6.8 | 1.6 | - | - |
| Liberia | 2.1 | 2.7 | - | - |
| Libya | 5.1 | 5.2 | - | 2.1 |
| Lithuania | 6.9 | 2.6 | 0.7 | 2.4 |
| Luxembourg | 2.7 | 0.6 | 0.0 | 0.5 |
| Macedonia | 0.6 | 1.8 | - | 1.7 |
| Madagascar | 2.1 | 14.8 | - | - |
| Malawi | 27.7 | 12.4 | - | - |
| Malaysia | 19.1 | 29.5 | - | 23.2 |
| Maldives | 32.4 | 0.5 | - | 0.4 |
| Mali | 0.5 | 9.3 | - | - |
| Malta | 20.3 | 0.4 | 0.1 | 0.4 |
| Marshall Islands | 0.4 | 0.0 | - | - |
| Mauritania | 0.1 | 3.1 | - | 0.7 |
| Mauritius | 4.6 | 1.1 | - | 0.8 |
| Mexico | 1.3 | 74.7 | - | 14.5 |
| Moldova | 128.9 | 3.5 | 0.1 | 3.3 |
| Monaco | 0.1 | 0.0 | - | 0.0 |
| Mongolia | 4.0 | 2.4 | - | 2.1 |
| Montenegro | 0.0 | 0.6 | 0.2 | 0.5 |
| Morocco | 3.3 | 28.2 | - | 25.2 |
| Mozambique | 0.6 | 16.3 | - | - |
| Myanmar | 36.9 | 43.5 | - | - |
| Namibia | 31.3 | 1.8 | - | 1.5 |
| Nauru | 54.4 | 0.0 | - | - |
| Nepal | 2.5 | 26.4 | - | 2.8 |
| Netherlands | 0.0 | 16.3 | 0.7 | 13.8 |
| New Zealand | 29.1 | 4.5 | 0.2 | 3.7 |
| Nicaragua | 17.1 | 6.4 | - | 4.3 |
| Niger | 4.8 | 10.5 | - | - |
| Nigeria | 6.6 | 111.8 | - | 102.9 |
| Niue | 24.2 | 0.0 | - | 0.0 |
| North Korea | 206.1 | - | - | - |
| Norway | 0.0 | 5.1 | 0.2 | 4.3 |
| Oman | 25.8 | 4.7 | - | 3.8 |
| Pakistan | 5.4 | 157.8 | 0.9 | 130.6 |
| Palau | 5.1 | 0.0 | 0.0 | 0.0 |
| Panama | 220.9 | 3.9 | 0.0 | 3.1 |
| Papua New Guinea | 0.0 | 5.2 | - | 5.2 |
| Paraguay | 4.3 | 6.4 | - | 5.5 |
| Peru | 8.9 | 30.1 | - | 23.4 |
| Philippines | 7.1 | 99.0 | - | 70.3 |
| Poland | 33.0 | 34.9 | 0.7 | 33.3 |
| Portugal | 109.6 | 9.8 | 0.3 | 8.6 |
| Qatar | 37.8 | 2.7 | - | 2.6 |
| Romania | 10.2 | 18.3 | - | 16.9 |
| Russian Federation | 2.9 | 113.1 | - | 114.8 |
| Rwanda | 19.2 | 8.8 | - | 8.8 |
| Saint Kitts and Nevis | 145.9 | 0.0 | - | 0.0 |
| Saint Lucia | 13.0 | 0.2 | - | 0.1 |
| Saint Vincent and the Grenadines | 0.1 | 0.1 | - | 0.1 |
| Samoa | 0.2 | 0.1 | - | 0.1 |
| San Marino | 0.1 | 0.0 | - | 0.0 |
| Sao Tome and Principe | 0.2 | 0.1 | - | 0.0 |
| Saudi Arabia | 0.0 | 31.2 | - | 24.9 |
| Senegal | 0.2 | 10.8 | - | 8.5 |
| Serbia | 34.8 | 7.7 | - | 7.1 |
| Seychelles | 16.7 | 0.1 | - | 0.1 |
| Sierra Leone | 8.7 | 4.2 | - | 0.4 |
| Singapore | 0.1 | 5.6 | 0.1 | 5.3 |
| Slovakia | 8.0 | 4.8 | - | 4.5 |
| Slovenia | 5.9 | 1.4 | 0.7 | 1.8 |
| Solomon Islands | 5.5 | 0.5 | - | 0.0 |
| Somalia | 2.1 | 7.4 | - | - |
| South Africa | 0.7 | 45.5 | 2.5 | 39.2 |
| South Korea | 15.9 | 45.9 | - | 43.4 |
| South Sudan | 59.3 | 5.8 | - | - |
| Spain | 51.3 | 44.8 | 1.4 | 38.7 |
| Sri Lanka | 11.2 | 17.4 | - | 14.7 |
| Sudan | 46.8 | 23.4 | - | - |
| Suriname | 21.4 | 0.5 | - | 0.4 |
| Sweden | 43.8 | 8.7 | 0.3 | 8.0 |
| Switzerland | 0.6 | 8.2 | 0.3 | 7.6 |
| Syria | 10.1 | 11.2 | - | - |
| Tajikistan | 8.7 | 5.5 | - | 1.8 |
| Tanzania | 17.5 | 29.7 | - | - |
| Thailand | 9.5 | 66.2 | - | 55.7 |
| The Bahamas | 59.7 | 0.3 | 0.0 | 0.3 |
| The Gambia | 69.8 | 1.2 | - | - |
| Timor-Leste | 1.3 | 0.9 | - | 0.3 |
| Togo | 8.3 | 5.5 | - | 4.4 |
| Tonga | 0.1 | 0.1 | - | 0.0 |
| Trinidad and Tobago | 1.4 | 1.2 | - | 1.1 |
| Tunisia | 11.8 | 8.5 | - | 4.4 |
| Turkey | 84.3 | 68.2 | - | 60.1 |
| Turkmenistan | 6.0 | 3.9 | - | - |
| Tuvalu | 0.0 | 0.0 | - | - |
| Uganda | 45.7 | 28.3 | - | 3.7 |
| Ukraine | 43.7 | 38.1 | - | 35.5 |
| United Arab Emirates | 9.9 | 9.5 | - | 8.3 |
| United Kingdom | 67.9 | 58.2 | 2.1 | 55.6 |
| United States | 331.0 | 311.3 | 11.1 | 282.9 |
| Uruguay | 3.5 | 3.2 | 0.1 | 2.6 |
| Uzbekistan | 33.5 | 25.5 | - | 25.5 |
| Vanuatu | 0.3 | 0.2 | - | - |
| Venezuela | 28.4 | 27.6 | - | 2.3 |
| Vietnam | 97.3 | 89.4 | 8.2 | 70.8 |
| Yemen | 29.8 | 16.3 | - | - |
| Zambia | 18.4 | 11.7 | - | 9.0 |
| Zimbabwe | 14.9 | 9.0 | - | 7.6 |

Abbreviation: AFR, African Region; AMR, Region of Americas; EMR, Eastern Mediterranean Region; EUR, European Region; SEAR, South-East Asia Region; WPR, Western Pacific Region.

# COVID-19 vaccine coverage

## *Metrics included in the dataset of administered doses*

(1) **Number of vaccine doses administered**. The cumulative administered number of COVID-19 vaccine doses stratified by time, vaccine technical platform, and age group, which might include initial doses, additional doses, and booster doses.

(2) **The number of people vaccinated at least one dose**. The number of people who have received the first dose of a multiple-dose vaccine or the dose administered for a one-dose vaccine.

(3) **The number of people fully vaccinated**. The number of people who have completed their primary immunization series according to the vaccination schedule.

(4) **The number of people receiving** **additional/booster doses**. The number of cumulative people receiving additional or/and booster doses.

## *Supplementary results*

### Table S7. The list of variables for investigating associations with vaccine coverage

| **Variables** | **Units** | **Temporal coverage** | **Source** |
| --- | --- | --- | --- |
| Socio-demographic index | Index | 2019 | GBD 2019 |
| Healthcare Access and Quality Index | Index | 2016 | GBD 2016 |
| Gross domestic product per capita | Purchasing power parity-adjusted dollars | 2019 | The World Bank |
| Physician density | Number of physicians per 10,000 | 2013-2019 | The World Bank |
| Government Health Spending per capita | Purchasing power parity-adjusted dollars | 2020 | GBD 2020 |

### Table S8. Analysis of multicollinearity

| **Variables** | **Variance inflation factors** |
| --- | --- |
| Socio-demographic index | 8.3 |
| Healthcare Access and Quality Index | 11.9 |
| Gross domestic product per capita | 5.9 |
| Physician density | 3.0 |
| Government Health Spending per capita | 5.3 |

### Figure S3. Proportion administered by vaccine technical platforms


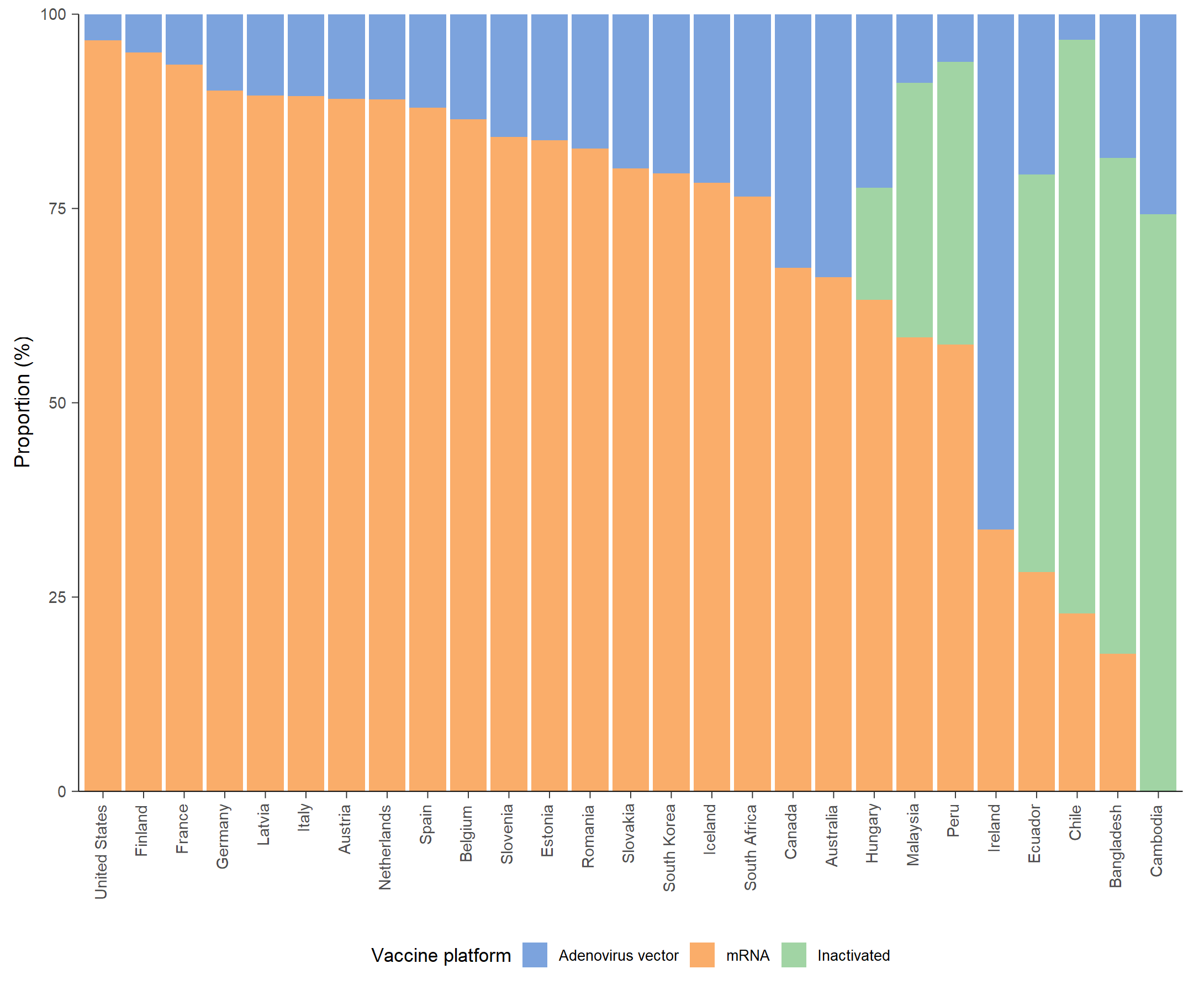


### Figure S4. Proportion administered by vaccine types.


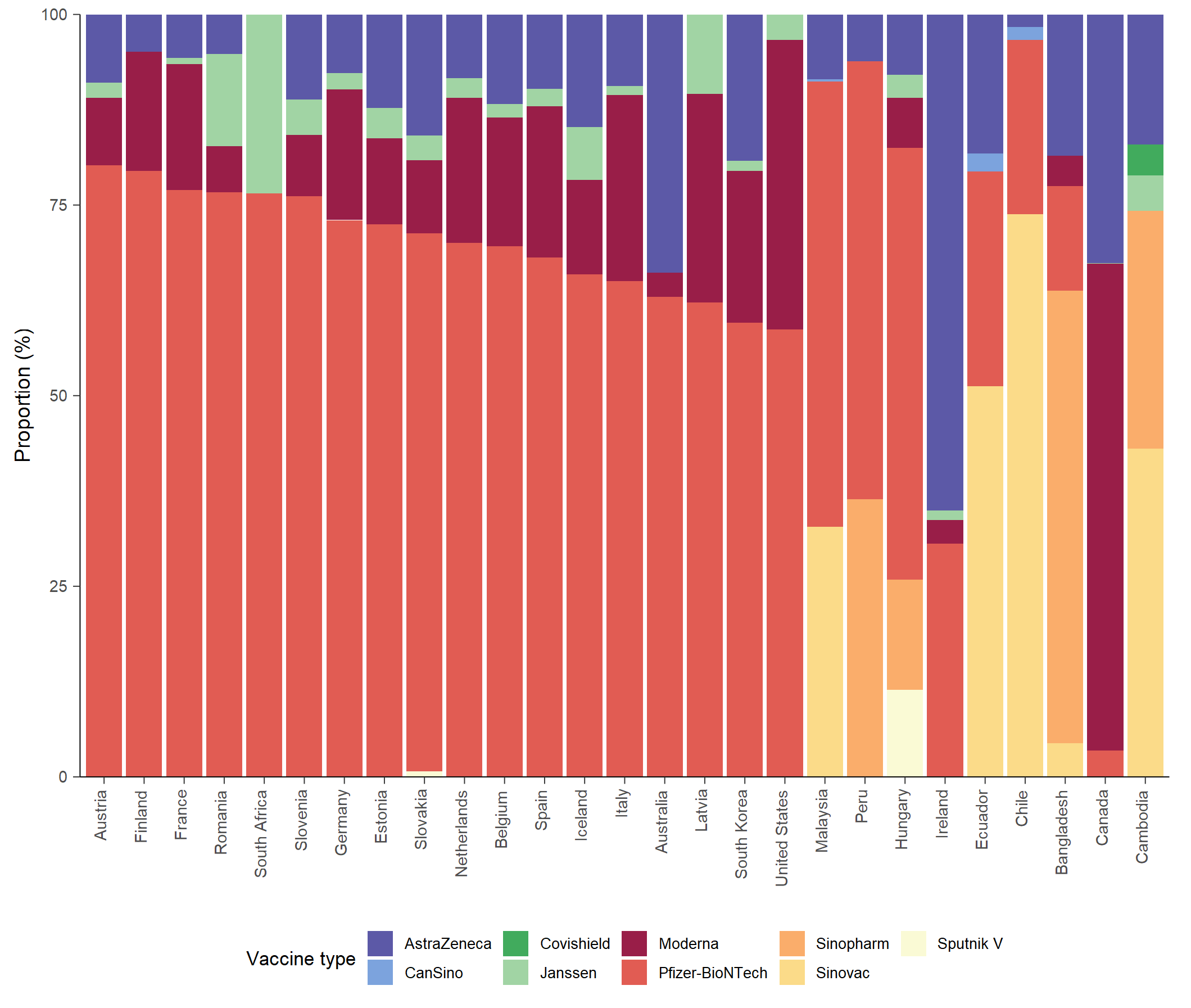


###

### Figure S5. Date at which achieved one dose per 100 people in total population by country.

The white areas represent countries that have not achieved one dose per 100 people or for which data are unavailable. The data shown here are as of January 31, 2022.


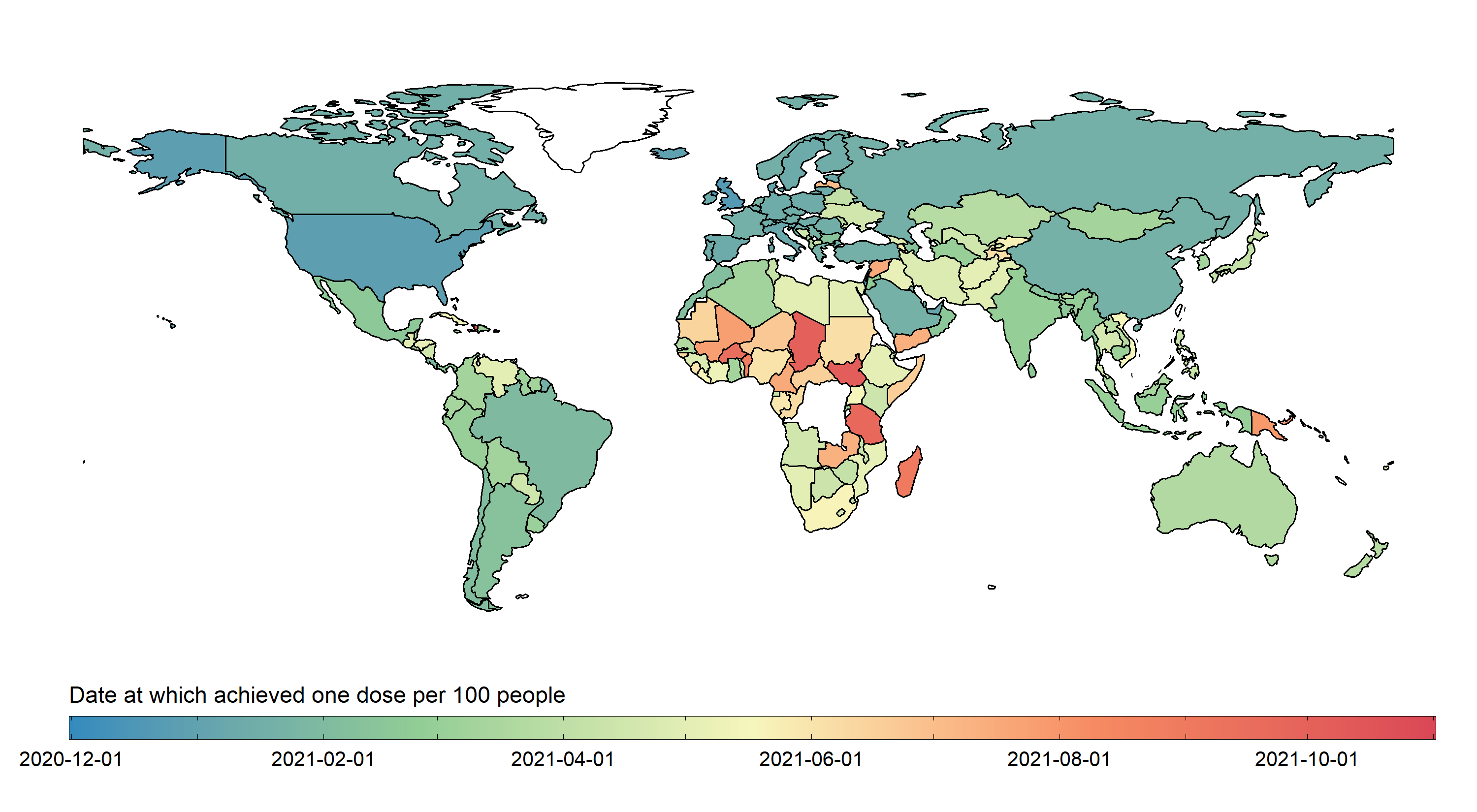


### Figure S6. Vaccine coverage over time stratified by income groups and role of vaccine seller/donor or recipient.

Cumulative doses per 100 people by income group in total population (A) and target population (B). Cumulative doses per 100 people by role of vaccine seller/donor or recipient in total population (C) and target population (D). Income group was defined by the World Bank. The data on role of vaccine seller/donor or recipient was derived from COVAX.


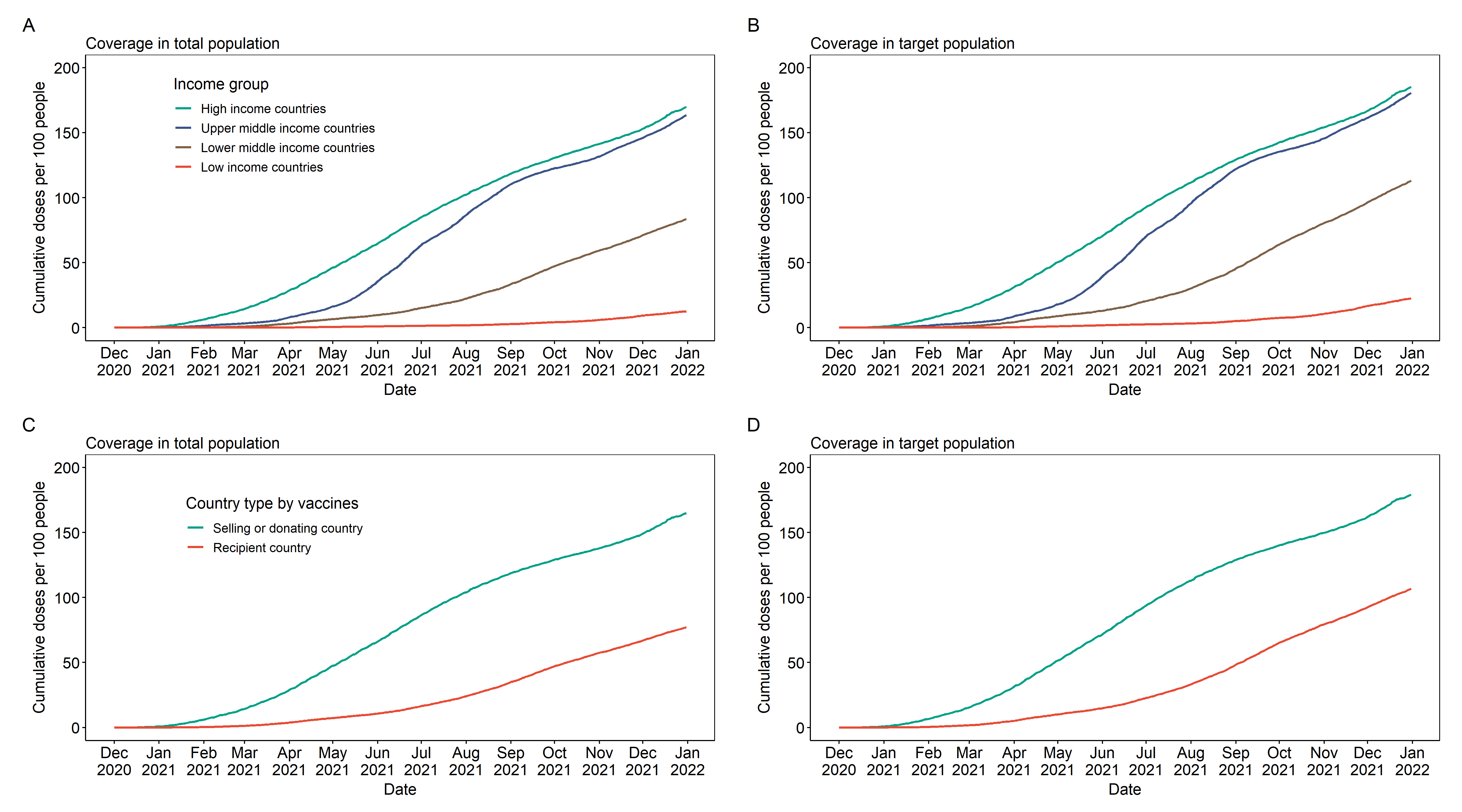


### Figure S7. Vaccine coverage stratified by SDI quintile and WHO region.

SDI, the socio-demographic index from the Institute for Health Metrics and Evaluation (IHME) (http://ghdx.healthdata.org/record/ihme-data/gbd-2019-socio-demographic-index-sdi-1950-2019).


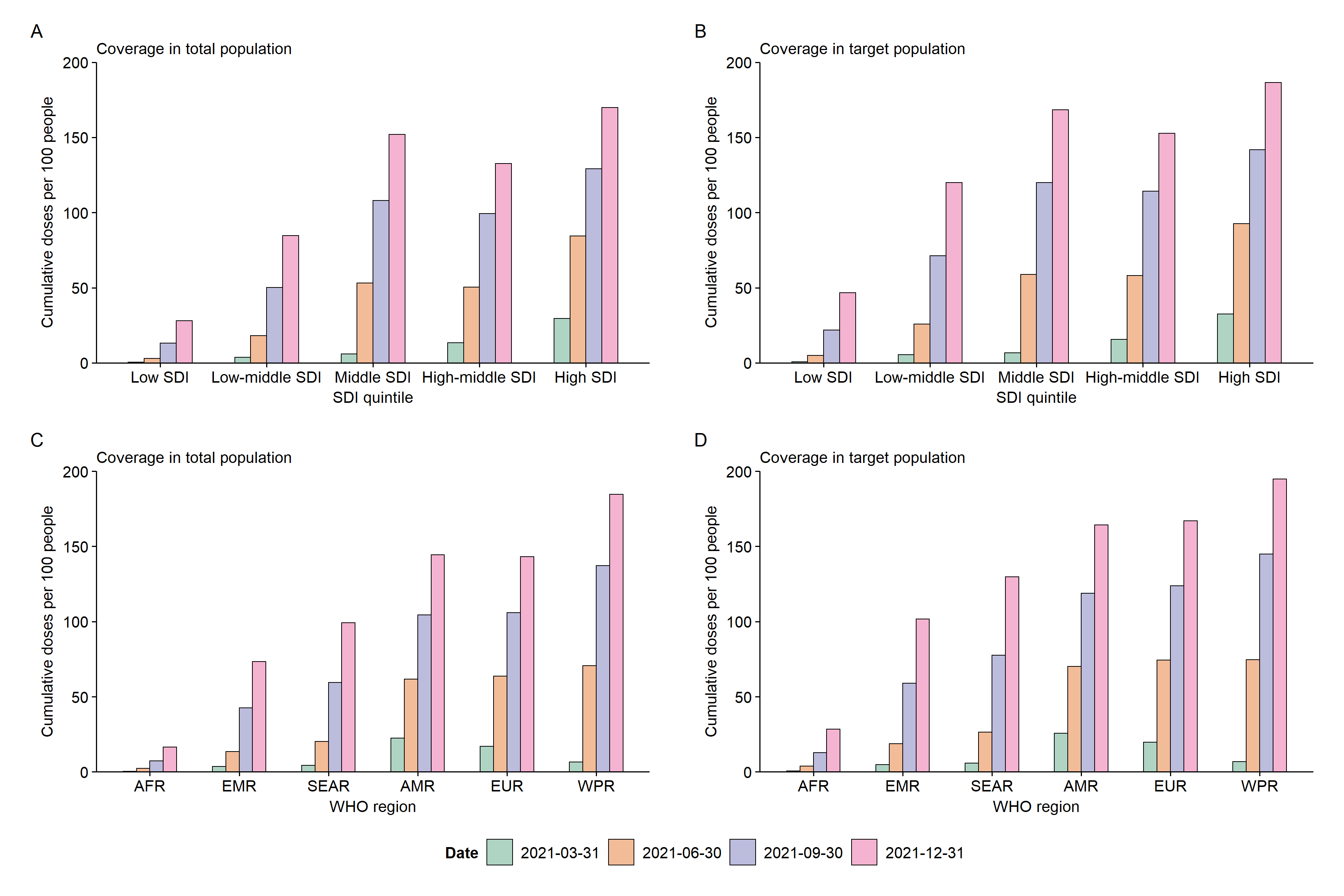


### Figure S8. The association between vaccine coverage with physician density and government health spending per capita.

Government health spending per capita has been adjusted for purchasing power parity. Solid line shows the linear or nonlinear fit.


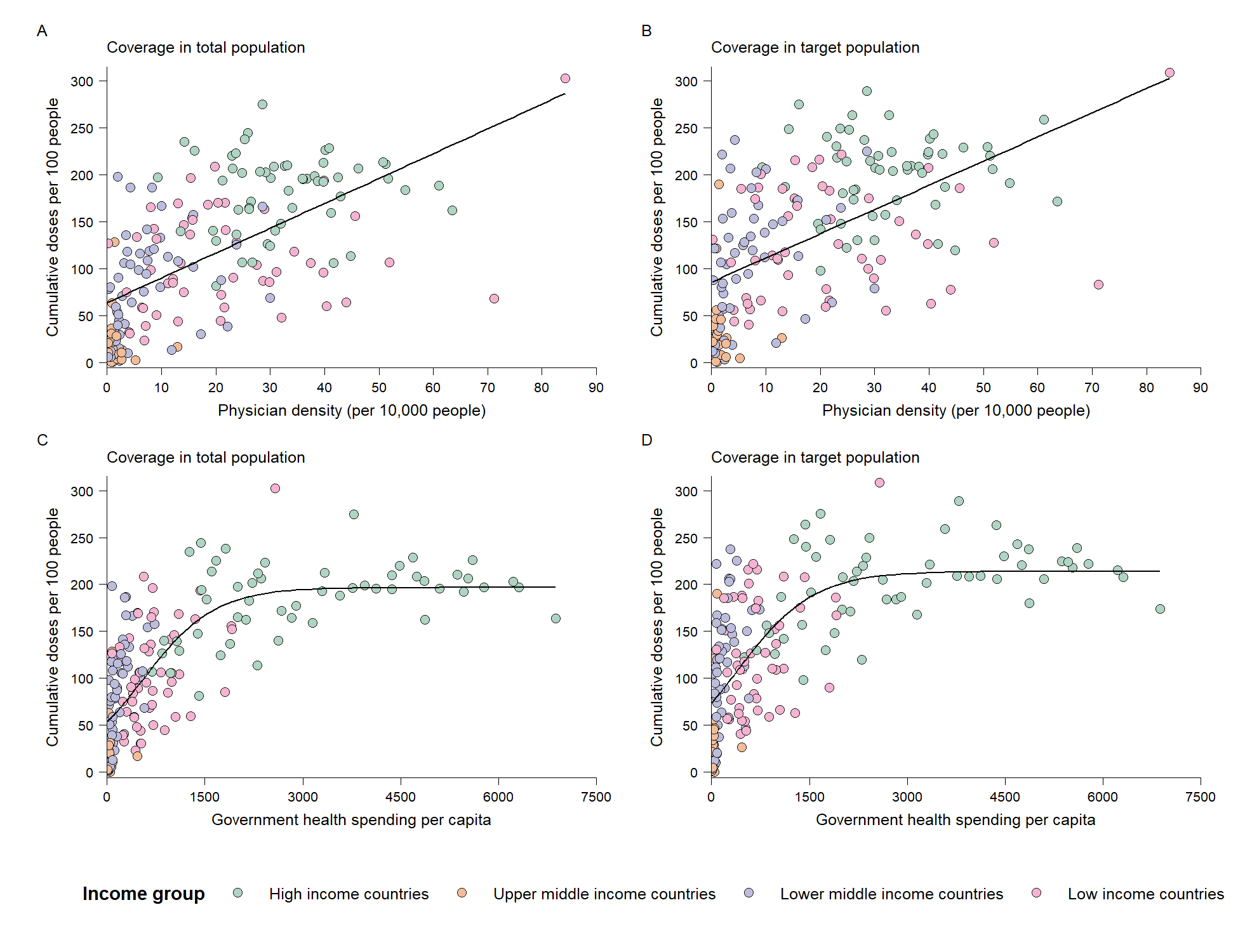


### Figure S9. Corrections between vaccine coverage and country-level vaccine acceptance.

Data on country-specific vaccine acceptance rates among general populations was summarized through a rapid review by selecting one representative study for each country, based on comprehensive evaluation about the study period, sampling methods and representativeness of study participants.


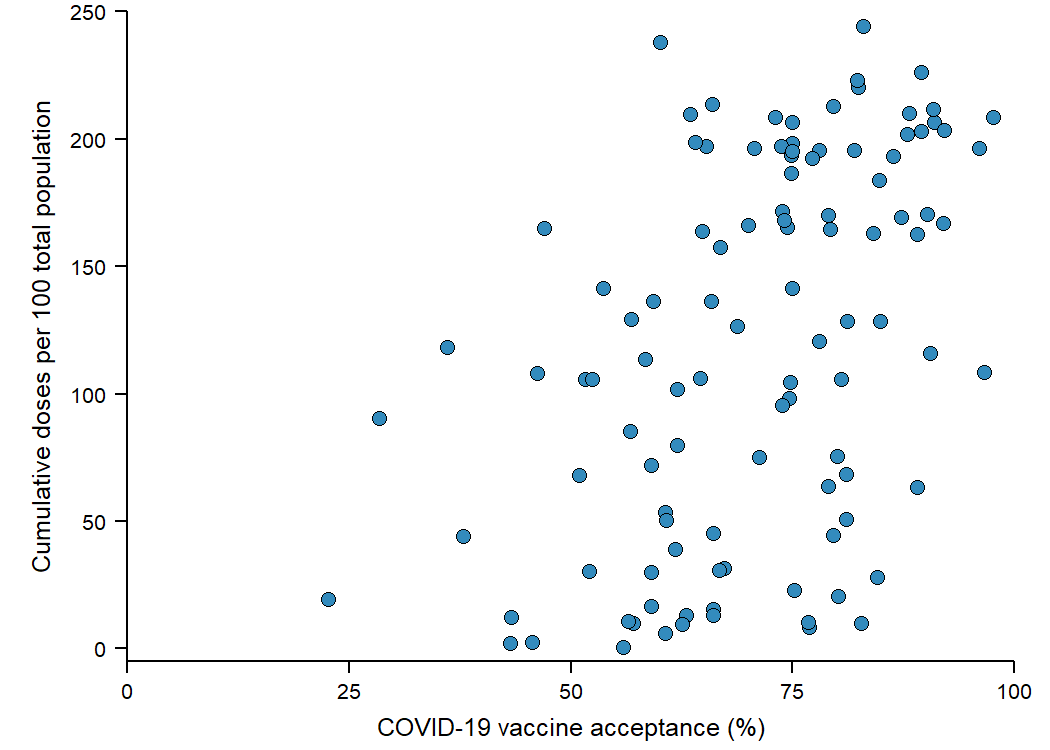


# Demand of COVID-19 vaccine doses

## *Method to calculate the* *demand of COVID-19 vaccine doses*

In primary immunization, we assumed a two-dose schedule to get the total doses required for primary immunization of target population. With an exception of previous infected COVID-19 patients, if countries recommended 1 dose for those groups, one-dose schedule was used instead. The target population for primary immunization was presented in Table S2.

In additional/booster immunization programs, we assumed a one-dose schedule for those target population recommended by each country, respectively (Table S3). Generally, the target groups for an additional dose mainly contained some specific population groups, e.g., people undergoing immunosuppression, and/or elderly, and/or specific population groups at high risk of infection (such as international travelers, front-line workers). While the target groups for a booster dose may include people with specific age, and/or people undergoing immunosuppression, and/or frontline workers, and/or people receiving Sinovac/Sinopharm vaccine.

By multiplying the number of specific target population and required doses, we obtained the doses required for primary and additional/booster immunization programs, respectively. Finally, we subtracted cumulative primary doses and additional/booster doses administered in each country to get the demand of COVID-19 vaccine doses for primary and additional/booster immunization programs, respectively (Table S7).

### Table S9. Global, regional, and national demand of vaccine dose.

| **Locations** | **Doses required for vaccination (millions)** | | | **Administered doses for vaccination (millions)** | | | **Current demand doses (millions)** | | |
| --- | --- | --- | --- | --- | --- | --- | --- | --- | --- |
|  | **Total** | **Primary doses** | **Additional/**  **booster doses** | **Total** | **Primary doses** | **Additional/**  **booster doses** | **Total** | **Primary doses** | **Additional/**  **Booster doses** |
| Global |  |  |  |  |  |  |  |  |  |
| Total | 16582.4 | 12398 | 4184.4 | 9986.6 | 8913.5 | 1073.1 | 6396.2 | 3284.8 | 3111.4 |
| WHO region |  |  |  |  |  |  |  |  |  |
| AFR | 1542.3 | 1300.7 | 241.6 | 193.9 | 192.3 | 1.6 | 1156.9 | 916.9 | 240 |
| AMR | 2474.9 | 1788.8 | 686.1 | 1615.2 | 1405.4 | 209.8 | 883.7 | 407.5 | 476.2 |
| EMR | 1407.3 | 1045.7 | 361.6 | 585.7 | 551.1 | 34.6 | 784.9 | 458 | 326.9 |
| EUR | 2295.9 | 1550.8 | 745.1 | 1441.3 | 1150.1 | 291.1 | 856.4 | 402.2 | 454.1 |
| SEAR | 3700.2 | 3049.1 | 651.2 | 2334.7 | 2301.2 | 33.5 | 1365.8 | 748.1 | 617.7 |
| WPR | 5161.9 | 3663 | 1498.9 | 3815.8 | 3313.3 | 502.5 | 1348.5 | 352.1 | 996.4 |
| Countries |  |  |  |  |  |  |  |  |  |
| Afghanistan | 39.6 | 39.6 | - | 5.1 | 5.1 | - | 34.5 | 34.5 | - |
| Albania | 7 | 4.7 | 2.3 | 2.6 | 2.4 | 0.2 | 4.4 | 2.3 | 2.1 |
| Algeria | 57.1 | 57.1 | - | - | - | - | - | - | - |
| Andorra | 0.2 | 0.1 | 0.1 | 0.1 | 0.1 | - | 0.1 | 0 | 0.1 |
| Angola | 55.7 | 40.3 | 15.4 | 14.9 | 14.9 | - | 40.8 | 25.4 | 15.4 |
| Antigua and Barbuda | 0.2 | 0.2 | 0.1 | 0.1 | 0.1 | - | 0.1 | 0 | 0.1 |
| Argentina | 116.4 | 84.4 | 32 | 87.6 | 74.7 | 12.9 | 28.8 | 9.7 | 19.2 |
| Armenia | 7.2 | 4.9 | 2.2 | 1.9 | 1.9 | 0 | 5.3 | 3 | 2.2 |
| Australia | 68 | 47.7 | 20.4 | 49.9 | 41.9 | 8 | 18.2 | 5.8 | 12.4 |
| Austria | 25.7 | 17.1 | 8.5 | 17.6 | 13.2 | 4.4 | 8 | 4 | 4.1 |
| Azerbaijan | 23.3 | 15.9 | 7.4 | 12 | 10 | 2 | 11.3 | 5.9 | 5.3 |
| Bahrain | 4.7 | 3.2 | 1.4 | 3.4 | 2.4 | 0.9 | 1.3 | 0.8 | 0.5 |
| Bangladesh | 288.8 | 259.5 | 29.3 | 156.4 | 155.2 | 1.3 | 132.4 | 104.3 | 28.1 |
| Barbados | 0.7 | 0.5 | 0.2 | 0.3 | 0.3 | - | 0.4 | 0.2 | 0.2 |
| Belarus | 23.3 | 15.8 | 7.5 | 10 | 10 | - | 13.3 | 5.8 | 7.5 |
| Belgium | 31.1 | 21.9 | 9.2 | 24.2 | 17.6 | 6.6 | 7 | 4.3 | 2.7 |
| Belize | 0.7 | 0.6 | 0.1 | 0.4 | 0.4 | - | 0.2 | 0.2 | 0.1 |
| Benin | 15.8 | 15.8 | - | 2.3 | 2.3 | - | 13.5 | 13.5 | - |
| Bhutan | 1.3 | 1.2 | 0.1 | 1.4 | 1.4 | - | 0.1 | 0 | 0.1 |
| Bolivia | 28.4 | 21 | 7.5 | 11.9 | 11 | 0.9 | 16.6 | 10 | 6.6 |
| Bosnia and Herzegovina | 5.8 | 5.8 | - | 1.9 | 1.8 | 0.1 | 4 | 4 | - |
| Botswana | 4.9 | 3.4 | 1.4 | - | - | - | 1.4 | - | 1.4 |
| Brazil | 555.1 | 396.2 | 159 | 362.5 | 315.2 | 47.3 | 192.6 | 80.9 | 111.7 |
| Brunei | 1 | 0.7 | 0.3 | 1 | 1 | - | 0.3 | 0 | 0.3 |
| Bulgaria | 19.4 | 13.3 | 6.2 | 4.2 | 3.6 | 0.6 | 15.3 | 9.7 | 5.5 |
| Burkina Faso | 20.4 | 20.4 | - | - | - | - | - | - | - |
| Burundi | 11.5 | 11.5 | - | 0 | 0 | - | 11.5 | 11.5 | - |
| Cambodia | 40.5 | 29.9 | 10.7 | 33.1 | 27.1 | 6.1 | 7.4 | 2.8 | 4.6 |
| Cameroon | 34.6 | 34.6 | - | - | - | - | - | - | - |
| Canada | 102.1 | 71.5 | 30.6 | 78 | 62.5 | 15.5 | 24.1 | 9 | 15.1 |
| Cabo Verde | 1.1 | 0.9 | 0.3 | 0.7 | 0.7 | - | 0.5 | 0.2 | 0.3 |
| Central African Republic | 4.7 | 4.7 | - | 0.7 | 0.7 | - | 4.1 | 4.1 | - |
| Chad | 15.3 | 15.3 | - | 0.4 | 0.4 | - | 14.9 | 14.9 | - |
| Chile | 51.6 | 35.4 | 16.2 | 46.7 | 34 | 12.7 | 4.9 | 1.4 | 3.5 |
| China | 3914.7 | 2779.5 | 1135.2 | 3000.5 | 2568.9 | 431.7 | 914.1 | 210.6 | 703.5 |
| Colombia | 136.3 | 94.3 | 42 | 71.9 | 66.2 | 5.7 | 64.4 | 28.1 | 36.2 |
| Comoros | 1.2 | 1.2 | - | 0.6 | 0.6 | - | 0.6 | 0.6 | - |
| Congo | 5.8 | 5.8 | - | - | - | - | - | - | - |
| Cook Islands | 0 | 0 | 0 | 0 | 0 | - | 0 | 0 | 0 |
| Costa Rica | 10.5 | 9.5 | 1 | 8.3 | 7.7 | 0.6 | 2.1 | 1.8 | 0.4 |
| Cote d'Ivoire | 27.3 | 27.3 | - | 7.6 | 7.6 | - | 19.7 | 19.7 | - |
| Croatia | 7.8 | 7.8 | - | 5.1 | 5.1 | - | 2.7 | 2.7 | - |
| Cuba | 32.3 | 21.2 | 11.1 | 34.3 | 28.9 | 5.4 | 5.8 | 0 | 5.8 |
| Cyprus | 3.2 | 2.3 | 1 | 1.7 | 1.3 | 0.4 | 1.6 | 1 | 0.6 |
| Czechia | 29.7 | 20.3 | 9.4 | 17.1 | 13.3 | 3.8 | 12.6 | 7 | 5.6 |
| Democratic Republic of the Congo | 85.3 | 85.3 | - | 0.5 | 0.5 | - | 84.8 | 84.8 | - |
| Denmark | 15.6 | 11 | 4.6 | 13.1 | 9.5 | 3.6 | 2.5 | 1.4 | 1.1 |
| Djibouti | 1.3 | 1.3 | - | 0.2 | 0.2 | - | 1.1 | 1.1 | - |
| Dominica | 0.1 | 0.1 | 0 | 0.1 | 0.1 | - | 0.1 | 0 | 0 |
| Dominican Republic | 24.2 | 16.9 | 7.3 | 14.8 | 12.8 | 1.9 | 9.4 | 4.1 | 5.3 |
| Ecuador | 43.8 | 32 | 11.9 | 30 | 27.4 | 2.6 | 13.8 | 4.5 | 9.3 |
| Egypt | 219.5 | 146.3 | 73.2 | 65.1 | 65.1 | - | 154.4 | 81.3 | 73.2 |
| El Salvador | 16 | 11.6 | 4.4 | 10 | 8.7 | 1.2 | 6 | 2.9 | 3.2 |
| Equatorial Guinea | 1.6 | 1.6 | - | 0.5 | 0.5 | - | 1.2 | 1.2 | - |
| Eritrea | - | - | - | - | - | - | - | - | - |
| Estonia | 3.2 | 2.2 | 1.1 | 1.5 | 1.5 | - | 1.7 | 0.7 | 1.1 |
| Eswatini | 2.3 | 1.6 | 0.6 | 0.5 | 0.5 | - | 1.8 | 1.2 | 0.6 |
| Ethiopia | 154.5 | 154.5 | - | 11 | 11 | - | 143.5 | 143.5 | - |
| Fiji | 0.3 | 0.2 | 0.1 | - | - | - | 0.1 | - | 0.1 |
| Finland | 2 | 1.4 | 0.6 | 1.3 | 1.3 | - | 0.7 | 0.1 | 0.6 |
| France | 14.5 | 10 | 4.5 | 11 | 8.5 | 2.5 | 3.5 | 1.5 | 2 |
| Gabon | 141.4 | 85.3 | 56.2 | 136.8 | 104.2 | 32.6 | 23.6 | 0 | 23.6 |
| Georgia | 2.6 | 2.6 | - | 0.5 | 0.5 | - | 2 | 2 | - |
| Germany | 9.6 | 6.6 | 3.1 | 2.7 | 2.7 | - | 6.9 | 3.8 | 3.1 |
| Ghana | 213.4 | 139 | 74.4 | 165.2 | 121 | 44.2 | 48.2 | 17.9 | 30.3 |
| Greece | 44.9 | 39.1 | 5.9 | 10 | 10 | - | 34.9 | 29.1 | 5.9 |
| Grenada | 28.7 | 20 | 8.7 | 19.2 | 14.3 | 4.8 | 9.6 | 5.7 | 3.9 |
| Guatemala | 0.3 | 0.2 | 0.1 | 0.1 | 0.1 | - | 0.2 | 0.1 | 0.1 |
| Guinea | 38.4 | 25.3 | 13.1 | 13.5 | 12.2 | 1.2 | 25 | 13.1 | 11.9 |
| Guinea-Bissau | 16.9 | 16.9 | - | 4.7 | 4.7 | - | 12.2 | 12.2 | - |
| Guyana | 3.6 | 2.6 | 1 | 0.4 | 0.4 | - | 3.2 | 2.1 | 1 |
| Haiti | 1.9 | 1.4 | 0.5 | 0.8 | 0.7 | 0 | 1.2 | 0.7 | 0.5 |
| Honduras | 14 | 14 | - | 0.2 | 0.2 | - | 13.8 | 13.8 | - |
| Hungary | 22 | 15.8 | 6.3 | 10.5 | 9.5 | 1 | 11.6 | 6.3 | 5.3 |
| Iceland | 27 | 18.4 | 8.6 | 15.9 | 12.2 | 3.7 | 11 | 6.2 | 4.9 |
| India | 0.9 | 0.6 | 0.3 | 0.8 | 0.5 | 0.2 | 0.1 | 0.1 | 0 |
| Indonesia | 2347.9 | 1988.7 | 359.2 | 1664.8 | 1653 | 11.8 | 683.2 | 335.8 | 347.4 |
| Iran | 678.5 | 489.9 | 188.6 | 286.1 | 286.1 | - | 392.4 | 203.8 | 188.6 |
| Iraq | 212.6 | 152.7 | 59.8 | 132.2 | 114.9 | 17.3 | 80.4 | 37.8 | 42.5 |
| Ireland | 65.5 | 55.4 | 10.1 | 15.7 | 15.7 | - | 49.8 | 39.7 | 10.1 |
| Israel | 13.1 | 9.2 | 3.8 | 10.4 | 7.7 | 2.7 | 2.7 | 1.6 | 1.1 |
| Italy | 22.3 | 15.6 | 6.7 | 17.9 | 12.8 | 5.1 | 4.4 | 2.8 | 1.6 |
| Jamaica | 170.6 | 116.3 | 54.3 | 128.7 | 95.1 | 33.6 | 42 | 21.2 | 20.8 |
| Japan | 6.9 | 4.8 | 2.1 | 1.3 | 1.3 | 0 | 5.6 | 3.5 | 2.1 |
| Jordan | 335.5 | 228.2 | 107.3 | 205.7 | 201.3 | 4.5 | 129.8 | 26.9 | 102.9 |
| Kazakhstan | 20.8 | 14.6 | 6.2 | 9.2 | 9.2 | - | 11.6 | 5.4 | 6.2 |
| Kenya | 41 | 28.4 | 12.6 | 17.9 | 17.9 | - | 23.1 | 10.5 | 12.6 |
| Kiribati | 95.4 | 66 | 29.3 | 12.3 | 12.1 | 0.1 | 83.1 | 53.9 | 29.2 |
| Kuwait | 0.1 | 0.1 | - | 0.1 | 0.1 | - | 0 | 0 | - |
| Kyrgyzstan | 11.3 | 8 | 3.3 | 7.3 | 7.3 | - | 3.9 | 0.6 | 3.3 |
| Laos | 11.9 | 7.8 | 4.1 | 2.5 | 2.5 | - | 9.4 | 5.3 | 4.1 |
| Latvia | 10.8 | 10.8 | 0 | 8.6 | 8.6 | - | 2.2 | 2.2 | 0 |
| Lebanon | 4.7 | 3.2 | 1.5 | 2.8 | 2.3 | 0.4 | 1.9 | 0.8 | 1.1 |
| Lesotho | 18 | 12.5 | 5.4 | 4.9 | 4.5 | 0.4 | 13.1 | 8.1 | 5 |
| Liberia | 3.2 | 3.2 | - | - | - | - | - | - | - |
| Libya | 5.3 | 5.3 | - | - | - | - | - | - | - |
| Lithuania | 12.5 | 10.4 | 2.1 | 3.1 | 3.1 | - | 9.5 | 7.3 | 2.1 |
| Luxembourg | 7.6 | 5.2 | 2.5 | 4.4 | 3.5 | 0.9 | 3.2 | 1.6 | 1.6 |
| Macedonia | 1.7 | 1.2 | 0.5 | 1.2 | 0.9 | 0.3 | 0.5 | 0.3 | 0.2 |
| Madagascar | 5.3 | 3.6 | 1.7 | 1.8 | 1.7 | 0.1 | 3.5 | 1.9 | 1.5 |
| Malawi | 29.5 | 29.5 | - | 1.1 | 1.1 | - | 28.4 | 28.4 | - |
| Malaysia | 24.8 | 24.8 | - | 1.9 | 1.9 | - | 22.9 | 22.9 | - |
| Maldives | 82.2 | 59 | 23.2 | 63.6 | 51.6 | 12 | 18.6 | 7.4 | 11.2 |
| Mali | 1.3 | 0.9 | 0.4 | 0.8 | 0.8 | 0.1 | 0.5 | 0.1 | 0.3 |
| Malta | 18.6 | 18.6 | - | 1.7 | 1.7 | - | 17 | 17 | - |
| Marshall Islands | 1.2 | 0.8 | 0.4 | 1.2 | 0.9 | 0.3 | 0 | 0 | 0 |
| Mauritania | 0.1 | 0.1 | - | - | - | - | - | - | - |
| Mauritius | 6.9 | 6.2 | 0.7 | 2.5 | 2.5 | - | 4.4 | 3.7 | 0.7 |
| Mexico | 3 | 2.2 | 0.8 | - | - | - | 0.8 | - | 0.8 |
| Micronesia | 163.9 | 149.5 | 14.5 | 165.8 | 165.8 | - | 14.5 | 0 | 14.5 |
| Moldova | 10.3 | 7 | 3.3 | 1.9 | 1.8 | 0.2 | 8.4 | 5.3 | 3.1 |
| Monaco | 0.1 | 0.1 | 0 | - | - | - | 0 | - | 0 |
| Mongolia | 7 | 4.8 | 2.1 | 5.4 | 4.4 | 1 | 1.5 | 0.4 | 1.1 |
| Montenegro | 1.7 | 1.2 | 0.5 | 0.7 | 0.6 | 0.1 | 1 | 0.6 | 0.4 |
| Morocco | 81.6 | 56.3 | 25.2 | 52.3 | 52.3 | - | 29.3 | 4.1 | 25.2 |
| Mozambique | 32.6 | 32.6 | - | 19.8 | 19.8 | - | 12.8 | 12.8 | - |
| Myanmar | 87 | 87 | - | 41 | 41 | - | 46 | 46 | - |
| Namibia | 5 | 3.5 | 1.5 | 0.8 | 0.8 | - | 4.2 | 2.8 | 1.5 |
| Nauru | 0 | 0 | - | 0 | 0 | - | 0 | 0 | - |
| Nepal | 55.7 | 52.9 | 2.8 | 31.6 | 31.6 | - | 24 | 21.2 | 2.8 |
| Netherlands | 42.1 | 28.2 | 13.8 | 33.4 | 24.9 | 8.6 | 8.6 | 3.4 | 5.3 |
| New Zealand | 12.7 | 9 | 3.7 | 9.5 | 8.1 | 1.4 | 3.3 | 0.9 | 2.3 |
| Nicaragua | 17 | 12.7 | 4.3 | 8.8 | 8.8 | - | 8.2 | 3.9 | 4.3 |
| Niger | 21.1 | 21.1 | - | 1.8 | 1.8 | - | 19.3 | 19.3 | - |
| Nigeria | 326.5 | 223.6 | 102.9 | 20.6 | 20.6 | - | 305.9 | 203 | 102.9 |
| Niue | 0 | 0 | 0 | - | - | - | 0 | - | 0 |
| North Korea | - | - | - | - | - | - | - | - | - |
| Norway | 14.5 | 10.2 | 4.3 | 11 | 8.3 | 2.7 | 3.5 | 1.9 | 1.6 |
| Oman | 13.1 | 9.3 | 3.8 | 6.6 | 6.6 | - | 6.5 | 2.7 | 3.8 |
| Pakistan | 446.3 | 315.7 | 130.7 | 176.8 | 174.3 | 2.5 | 269.5 | 141.4 | 128.2 |
| Palau | 0 | 0 | 0 | - | - | - | 0 | - | 0 |
| Panama | 11 | 7.9 | 3.1 | 6.6 | 5.6 | 0.9 | 4.4 | 2.2 | 2.2 |
| Papua New Guinea | 15.7 | 10.5 | 5.2 | 0.5 | 0.5 | - | 15.2 | 10 | 5.2 |
| Paraguay | 18.3 | 12.9 | 5.5 | 7.6 | 6.7 | 0.9 | 10.8 | 6.2 | 4.6 |
| Peru | 83.6 | 60.3 | 23.4 | 55.8 | 47.9 | 7.9 | 27.8 | 12.3 | 15.5 |
| Philippines | 268.3 | 197.9 | 70.3 | 127 | 119.4 | 7.5 | 141.3 | 78.5 | 62.8 |
| Poland | 103.1 | 69.8 | 33.3 | 51.7 | 41.4 | 10.3 | 51.4 | 28.4 | 23 |
| Portugal | 25.5 | 17 | 8.6 | 21.6 | 16.5 | 5.1 | 4 | 0.5 | 3.5 |
| Qatar | 8 | 5.4 | 2.6 | 5.8 | 5.8 | - | 2.6 | 0 | 2.6 |
| Romania | 53.5 | 36.6 | 16.9 | 16.4 | 16.4 | - | 37.1 | 20.2 | 16.9 |
| Russia | 340.9 | 226.2 | 114.8 | 154.4 | 144.3 | 10.1 | 186.6 | 81.9 | 104.7 |
| Rwanda | 26.3 | 17.5 | 8.8 | 16.6 | 15.7 | 0.8 | 9.7 | 1.8 | 7.9 |
| Saint Kitts and Nevis | 0.1 | 0.1 | 0 | 0.1 | 0.1 | 0 | 0 | 0 | 0 |
| Saint Lucia | 0.5 | 0.3 | 0.1 | 0.1 | 0.1 | - | 0.3 | 0.2 | 0.1 |
| Saint Vincent and the Grenadines | 0.3 | 0.2 | 0.1 | 0.1 | 0.1 | 0 | 0.2 | 0.1 | 0.1 |
| Samoa | 0.4 | 0.3 | 0.1 | 0.3 | 0.3 | - | 0.1 | 0 | 0.1 |
| San Marino | 0.1 | 0 | 0 | 0.1 | 0.1 | - | 0 | 0 | 0 |
| Sao Tome and Principe | 0.3 | 0.3 | 0 | 0.2 | 0.2 | - | 0.1 | 0.1 | 0 |
| Saudi Arabia | 87.2 | 62.3 | 24.9 | 57.3 | 49.3 | 8.1 | 29.8 | 13 | 16.8 |
| Senegal | 30.2 | 21.7 | 8.5 | 2 | 2 | - | 28.1 | 19.6 | 8.5 |
| Serbia | 22.5 | 15.4 | 7.1 | 8.4 | 6.6 | 1.8 | 14.1 | 8.8 | 5.3 |
| Seychelles | 0.2 | 0.2 | 0.1 | 0.2 | 0.2 | - | 0.1 | 0 | 0.1 |
| Sierra Leone | 8.9 | 8.4 | 0.4 | 1.4 | 1.4 | - | 7.4 | 7 | 0.4 |
| Singapore | 16.1 | 10.8 | 5.3 | 12.9 | 9.7 | 3.2 | 3.2 | 1.1 | 2.1 |
| Slovakia | 14 | 9.6 | 4.5 | - | - | - | 4.5 | - | 4.5 |
| Slovenia | 4.7 | 2.8 | 1.9 | 2.9 | 2.3 | 0.6 | 1.8 | 0.5 | 1.3 |
| Solomon Islands | 0.9 | 0.9 | 0 | 0.3 | 0.3 | - | 0.7 | 0.6 | 0 |
| Somalia | 14.9 | 14.9 | - | - | - | - | - | - | - |
| South Africa | 130.3 | 90.9 | 39.4 | 29.9 | 29.3 | 0.6 | 100.4 | 61.6 | 38.8 |
| South Korea | 135.2 | 91.8 | 43.4 | 114.4 | 87.1 | 27.3 | 20.8 | 4.6 | 16.2 |
| South Sudan | 11.7 | 11.7 | - | 0.3 | 0.3 | - | 11.3 | 11.3 | - |
| Spain | 118.2 | 79.5 | 38.7 | 90.3 | 68.6 | 21.7 | 27.9 | 10.9 | 17 |
| Sri Lanka | 49.4 | 34.7 | 14.7 | 35.8 | 30.6 | 5.2 | 13.7 | 4.2 | 9.5 |
| Sudan | 46.9 | 46.9 | - | 3.7 | 3.7 | - | 43.2 | 43.2 | - |
| Suriname | 1.3 | 0.9 | 0.4 | 0.5 | 0.5 | - | 0.8 | 0.4 | 0.4 |
| Sweden | 25.3 | 17.3 | 8 | 19.4 | 15.5 | 4 | 5.9 | 1.9 | 4 |
| Switzerland | 24 | 16.4 | 7.6 | 15.3 | 11.9 | 3.4 | 8.7 | 4.5 | 4.2 |
| Syria | 22.3 | 22.3 | - | - | - | - | - | - | - |
| Tajikistan | 12.8 | 11 | 1.8 | 8.3 | 8.3 | - | 4.5 | 2.7 | 1.8 |
| Tanzania | 59.4 | 59.4 | - | - | - | - | - | - | - |
| Thailand | 188.1 | 132.4 | 55.7 | 115.5 | 100.4 | 15.1 | 72.6 | 32 | 40.6 |
| The Bahamas | 1 | 0.7 | 0.3 | 0.3 | 0.3 | 0 | 0.7 | 0.4 | 0.3 |
| The Gambia | 2.4 | 2.4 | - | 0.3 | 0.3 | - | 2 | 2 | - |
| Timor-Leste | 2.1 | 1.9 | 0.3 | 1.2 | 1.2 | - | 0.9 | 0.6 | 0.3 |
| Togo | 15.4 | 11 | 4.4 | 2.5 | 2.5 | - | 12.9 | 8.5 | 4.4 |
| Tonga | 0.2 | 0.2 | 0 | 0.1 | 0.1 | - | 0 | 0 | 0 |
| Trinidad and Tobago | 3.4 | 2.4 | 1.1 | 1.5 | 1.4 | 0.1 | 1.9 | 1 | 1 |
| Tunisia | 21.3 | 17 | 4.4 | 12.8 | 11.7 | 1 | 8.6 | 5.2 | 3.3 |
| Turkey | 196.5 | 136.4 | 60.1 | 142 | 109.8 | 32.2 | 54.5 | 26.6 | 27.9 |
| Turkmenistan | 7.8 | 7.8 | - | - | - | - | - | - | - |
| Tuvalu | 0 | 0 | - | - | - | - | - | - | - |
| Uganda | 60.3 | 56.6 | 3.7 | 12.9 | 12.9 | - | 47.4 | 43.7 | 3.7 |
| Ukraine | 111.8 | 76.3 | 35.5 | 30 | 30 | - | 81.8 | 46.3 | 35.5 |
| United Arab Emirates | 27.3 | 19 | 8.3 | 23.5 | 19.2 | 4.3 | 4 | 0 | 4 |
| United Kingdom | 172.2 | 116.5 | 55.7 | 138.1 | 100.8 | 37.3 | 34.1 | 15.7 | 18.4 |
| United States | 905.7 | 622.7 | 283 | 541.5 | 452.6 | 88.9 | 364.2 | 170.1 | 194.1 |
| Uruguay | 9.1 | 6.5 | 2.6 | 7.4 | 5.6 | 1.8 | 1.7 | 0.9 | 0.8 |
| Uzbekistan | 76.6 | 51.1 | 25.5 | 42 | 42 | - | 34.6 | 9 | 25.5 |
| Vanuatu | 0.3 | 0.3 | - | 0.2 | 0.2 | - | 0.2 | 0.2 | - |
| Venezuela | 57.4 | 55.2 | 2.3 | 36 | 35.8 | 0.3 | 21.4 | 19.4 | 2 |
| Vietnam | 249.7 | 178.9 | 70.9 | 181.4 | 181.4 | - | 70.9 | 0 | 70.9 |
| Yemen | 32.6 | 32.6 | - | 0.7 | 0.7 | - | 31.8 | 31.8 | - |
| Zambia | 32.4 | 23.4 | 9 | 2.4 | 2.4 | - | 30 | 21 | 9 |
| Zimbabwe | 25.6 | 18 | 7.6 | 7.6 | 7.6 | - | 18 | 10.4 | 7.6 |

Abbreviation: AFR, African Region; AMR, Region of Americas; EMR, Eastern Mediterranean Region; EUR, European Region; SEAR, South-East Asia Region; WPR, Western Pacific Region.
